# Video-based marker-free tracking and multi-scale analysis of mouse locomotor activity and behavioral aspects in an open field arena: A perspective approach to the quantification of complex gait disturbances associated with Alzheimer's disease

**DOI:** 10.3389/fninf.2023.1101112

**Published:** 2023-02-02

**Authors:** Mikhail Bogachev, Aleksandr Sinitca, Konstantin Grigarevichius, Nikita Pyko, Asya Lyanova, Margarita Tsygankova, Eldar Davletshin, Konstantin Petrov, Tatyana Ageeva, Svetlana Pyko, Dmitrii Kaplun, Airat Kayumov, Yana Mukhamedshina

**Affiliations:** ^1^Centre for Digital Telecommunication Technologies, St. Petersburg Electrotechnical University “LETI”, St. Petersburg, Russia; ^2^Institute for Fundamental Medicine and Biology, Kazan Federal University, Kazan, Russia; ^3^FRC Kazan Scientific Center of RAS, Arbuzov Institute of Organic and Physical Chemistry, Kazan, Russia; ^4^Department of Histology, Cytology and Embryology, Kazan State Medical University, Kazan, Russia

**Keywords:** open field-test, animal behavior analysis, detrended partial cross-correlation analysis (DPCCA), Alzheimer's disease, fluctuation functions, video analysis, gait disturbance, DeepLabCut

## Abstract

**Introduction:**

Complex gait disturbances represent one of the prominent manifestations of various neurophysiological conditions, including widespread neurodegenerative disorders such as Alzheimer's and Parkinson's diseases. Therefore, instrumental measurement techniques and automatic computerized analysis appears essential for the differential diagnostics, as well as for the assessment of treatment effectiveness from experimental animal models to clinical settings.

**Methods:**

Here we present a marker-free instrumental approach to the analysis of gait disturbances in animal models. Our approach is based on the analysis of video recordings obtained with a camera placed underneath an open field arena with transparent floor using the DeeperCut algorithm capable of online tracking of individual animal body parts, such as the snout, the paws and the tail. The extracted trajectories of animal body parts are next analyzed using an original computerized methodology that relies upon a generalized scalable model based on fractional Brownian motion with parameters identified by detrended partial cross-correlation analysis.

**Results:**

We have shown that in a mouse model representative movement patterns are characterized by two asymptotic regimes characterized by integrated 1/f noise at small scales and nearly random displacements at large scales separated by a single crossover. More detailed analysis of gait disturbances revealed that the detrended cross-correlations between the movements of the snout, paws and tail relative to the animal body midpoint exhibit statistically significant discrepancies in the Alzheimer's disease mouse model compared to the control group at scales around the location of the crossover.

**Discussion:**

We expect that the proposed approach, due to its universality, robustness and clear physical interpretation, is a promising direction for the design of applied analysis tools for the diagnostics of various gait disturbances and behavioral aspects in animal models. We further believe that the suggested mathematical models could be relevant as a complementary tool in clinical diagnostics of various neurophysiological conditions associated with movement disorders.

## 1. Introduction

Gait disturbances appear one of the prominent indicators of various neurophysiological disorders including Alzheimer's and Parkinson's diseases where characteristic alterations of posture are widely used as both primary and differential diagnostic markers (O'keeffe et al., 1996; Muir et al., 2012; Cedervall et al., 2014). In recent years, several computerized solutions based on the instrumental assessment of gait and posture disturbances facilitating both diagnostic and treatment effectiveness assessment procedures have been proposed (Bachlin et al., 2009; Bächlin et al., 2010; Maquet et al., 2010; Chung et al., 2012; Margiotta et al., 2016; Mc Ardle et al., 2017; de Oliveira Silva et al., 2020). In animal model studies, computer-aided quantitative assessment of locomotor activity and associated behavioral aspects also attracted increasing attention (Li et al., 2013; Nyúl-Tóth et al., 2020; Nyul-Toth et al., 2021) (for a recent methodological review, see also Klein et al. (2022) and references therein).

Conventionally, animal behavioral and movement patterns have been measured and analyzed by direct observation and manual coding of behavioral categories (Anderson and Perona, 2014). In turn, using expert observation imposes severe limitations on the acquisition and analysis of behavioral data. First and foremost, it is a laborious and tedious task that severely limits the amount of data processed and the number of behaviors or behavioral variables analyzed. But, even more importantly, human analysis of behavior is prone to subjectivity. Behavior measurement strongly depends on human perceptual abilities, leaving a lot of room for human error and facilitating efficient tacit knowledge transfer in training. Furthermore, human understanding and interpretation of behavior is subjective and sometimes inconsistent.

Recent advancements in computer vision technologies lead to increasing availability of video analysis based information for animal behavioral tracking that finds extensive applications from fundamental animal biology and ecology to applied biomedical and pharmacological studies, respectively (Codling et al., 2008; Hooten et al., 2017), see also Reynolds (2009), Bearup et al. (2016), Hooten and Johnson (2017), and Torney et al. (2021). The purpose of the development of tools that promote more objective and quantifiable assessment and measurement of behavior (cf. Miklósi, 2014; Overall, 2014; Hall and Roshier, 2016) has long been acknowledged, recognizing the potential of technology not only to empower the human observer in terms of accuracy and volumes of processed data, but also to lead to discoveries of new characteristics of behavior which are inaccessible for human observation. Accordingly, computer-aided analysis of open field test data using fast, accurate, and cost-effective computer vision algorithms is essential for their objective and systematic classification and interpretation.

These considerations give rise to the emerging field of computational animal behavior analysis (CABA) (Anderson and Perona, 2014; Egnor and Branson, 2016), which aims to apply techniques from computer science and engineering to facilitate an accurate and objective analysis of behavior. Existing CABA tools mainly perform automatic tracking of animals; some of them can identify basic behavioral states and measure some behavioral parameters. Some well-established commercial systems, such as Ethovision, are highly costly and not always sufficiently flexible.

Examples of free access tools and platforms include platforms that allow the user to train or use existing machine learning models, such as the DeepLabCut Framework (Mathis et al., 2018) or the JAABA system (Kabra et al., 2013) for trajectory estimation, and systems that perform basic tracking and provide a limited set of parameters related to tracked trajectories, such as EZtrack (Pennington et al., 2019) and Pathfinder (Cooke et al., 2019). The first requires advanced programming skills and provides only the ability to track an animal or its body parts, while the latter is simpler to use. However, it analyzed specific behaviors (e.g., freezing) or behavioral tests only appropriate for some species (e.g., water maze, light-dark box).

Over several decades, the open field test remains the most widely used test for the quantitative characterization of behavioral patterns in experimental animal rodent models (Stanford, 2007; Gould et al., 2009; Perals et al., 2017; Sturman et al., 2018; Kraeuter et al., 2019). Over more than half a century, the open field test paradigm has developed into a powerful tool for the evaluation of animal locomotion and exploratory activity, as well as risk assessment and anxiety behavior, with a number of established quantitative markers for each of the behavioral characteristic studied. In the common behavioral test analysis procedure, once the animal movement trajectories have been extracted, their further analysis is commonly reduced to a number of predefined characteristics, in most cases represented by a set of scalar metrics. For example, in the open field rodent test locomotion characteristics include the total distance traveled and the total zone entries, vertical activity is characterized by the rear frequency, the rear duration and grooming, while the risk assessment is justified from the total stretch attend posture and the total sniffing events count, and the decision making patterns are typically interpreted based on the properties of the periphery zone return and corner zone return events.

Although the above characteristics typically have a clear underlying physical interpretation, being analyzed as single variables, they are often insufficient to characterize the whole complexity of the animal movement patterns. In turn, more sophisticated multiparametric models are required to extract further significant information partially hidden in the interactions between these characteristics. Very recently, we have shown that in the context of the fBm based models typical animal movement patterns are characterized by two asymptotic scaling regimes separated by a single crossover, and the position of this crossover depends explicitly on the neurophysiological condition of the experimental animal. Moreover, we have also shown that the identified animal movement model is explicitly associated with the conventional parameters such as the level crossing statistics characterizing zone transitions events, thus making the scalar metrics that are used in the conventional characterization of the open field test results reproducible from the model based perspective thus making the results clearly interpretable from the conventional point of view (Bogachev et al., 2023).

In this work, we report the results of an early validation of an in-house developed animal tracking tool and advanced model-based gait analysis characterized by high flexibility and low-level automation using DeepLabCut based video processing. To analyze animal movement trajectories and gait patterns, we employ several effective methods originating from statistical physics. The proposed methodology relies upon random walk class models (Jeanson et al., 2003; Patterson et al., 2008; Smouse et al., 2010; Langrock et al., 2014) including the (fractional) Brownian motion (fBm) and its modifications that in recent years attracted increasing attention in the context of animal tracking and behavioral analysis (Reynolds, 2009; Bearup et al., 2016; Hooten and Johnson, 2017; Torney et al., 2021). In marked contrast to multiple scalar movement parameters or models build upon their artificial combinations, fBm based models have only few free parameters that are easily physically interpretable (for further details, we refer to Codling et al., 2008 and references therein).

## 2. Materials and methods

### 2.1. Animals and experimental protocol

Double-transgenic mice used in this study express a chimeric mouse/human amyloid precursor protein (Mo/HuAPP695swe) and a mutant human presenilin 1 (PS1-dE9). Both mutations are associated with an early-onset Alzheimer's disease. The animals were 5- (*n* = 3) and 12-month (*n* = 9) old males or females which were previously purchased from the Laboratory Animal Breeding Facility (Branch of Shemyakin-Ovchinnikov Institute of Bioorganic Chemistry, Puschino, Moscow Region, Russia). The control group consisted of wild-type 5- (*n* = 3) and 12-month (*n* = 6) old mice. All animals were housed in plastic cages (3/4 mice/cage) with free access to food and water and were maintained under controlled conditions of humidity (50 ± 10), light (12/12 h light/dark cycle), and temperature (23°C ± 1). All experiments involving animals were performed in accordance with the guidelines set forth by the European Communities Council Directive 86/609/EEC. The experimental protocols were approved by the Animal Care and Use Committee of the Federal Research Center “Kazan Scientific Center of the Russian Academy of Sciences” (protocol No. 2, dated 09.06.2022).

### 2.2. Open field arena

The open field arena used as a test bed in this study was 33 × 22.3 × 25.8 cm in size. The floor was made of transparent PVC plastic. For spatial calibration, 2.5 cm Aruco codes have been added to all edges of the arena. Two light-saving sources located opposite to each other were used.

### 2.3. Data acquisition

**Figure 1:**
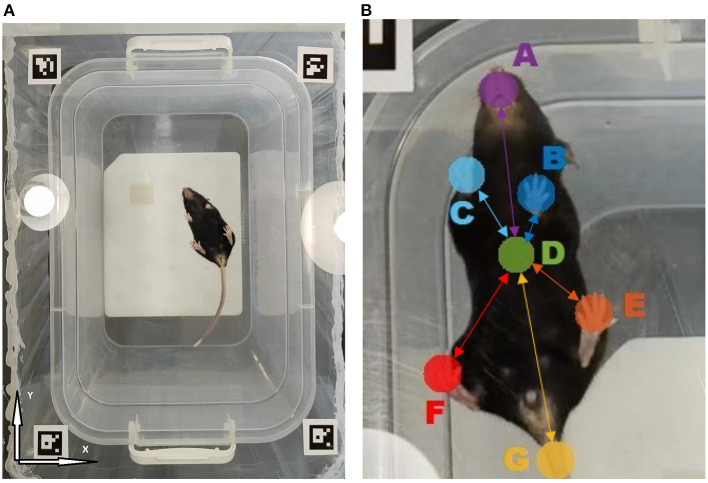
**(A)** The overall view of the open field arena with transparent floor as observed by the camera located underneath. **(B)** Tracked animal body parts: A- Snout, B- Left front paw, C- Right front paw, D- Body midpoint, E- Left hind paw, F- Right hind paw, G- Tail. Connections represent the animal movement network model, where nodes are associated with animal body parts and edges characterize their mutual dynamics. Highlighted edges form the network backbone consisting of links between various body parts and animal body midpoint, while secondary links denote all pairwise connections (not shown). Each link is characterized by some mutual movement metric, such as the detrended cross-correlation coefficient *R*_*ij*_ or partial correlation *P*_*ij*_, respectively.

For an explicit marker-free gait characterization, our approach implied the analysis of the animal video recorded from underneath. For that, a video camera Sony (Model: IMX766, 50 (12.5) Mpix, f/1.8, 1/1.56,” PDAF, OIS, 23.6 mm) providing 30 fps video sequence have been placed underneath the transparent floor of the open field arena. The continuous self-tuning feature of the camera was disabled. Video recordings have been obtained under similar conditions under normal ambient lighting (see Figure 1A). Altogether *n* = 23 recordings (*n* = 14 in the experimental group and *n* = 9 in the control group) of 15.2 min ± 20 s (median ± interquartile range) duration have been obtained, with one single recording per each animal.

### 2.4. Trajectory estimation

Acquired video frames have been initially downscaled to 720p for faster processing by DeepLabCut, an open source application widely adopted for tracking movement trajectories of various biological objects such as bacteria, various animals (leeches, fishes, rats, cheetahs, horses, humans) and their body parts (e.g., fingers, toes and even whiskers), as well as various artificial objects (e.g., robots). DeepLabCut utilized the feature detectors (ResNets + readout layers) of one of the state-of-the-art algorithms for human pose estimation by Insafutdinov et al. known as DeeperCut (Insafutdinov et al., 2016; Mathis et al., 2018; Nath et al., 2019). In this paper, we used a deep convolutional neural network ResNet50 (He et al., 2016). These networks utilizing the common weights concept are highly efficient in solving trajectory tracking tasks. The convolutional neural network consists of different types of layers: convolutional layers, subsampling layers and layers of a “normal” neural network, also known as a perceptron. The 50-layer ResNet uses a bottleneck design for the building block. A bottleneck residual block uses 1 × 1 convolutions, known as a “bottleneck,” which reduces the number of parameters and matrix multiplications. This enables much faster training of each layer. It uses a stack of three layers rather than two layers.

To form a dataset for the neural network training, the k-means algorithm was used. Using this algorithm, we extracted 50–100 frames from each video, depending on the complexity of the trajectory and poses of the objects under study. In the next step, seven different points for separate tracking, including the snout, all four paws and the base of the tail, as well as the animal body midpoint have been selected for separate tracking, as shown in the Figure 1. For each animal, the results of computer vision based analysis have been summarized in a CSV file containing trajectories for each of the tracked body parts in a table form. If the object could not be detected, then the gap was filled either with the previous value or with another nearest neighbor. Since the losses of the initial trajectories are around 10–20% of the entire trajectory duration, we next applied a median filter over five frames in order to reduce the effect of anomalies, including those induced by high-frequency jitter noise and short-time markers swapping, to enhance the overall noise robustness of the algorithm. Thus, the output CSV file did not contain any unfilled gaps in the trajectory.

### 2.5. Identification of the movement model

For the identification of the animal movement trajectory model and the estimation of its parameters from empirical trajectories, we employed the detrended partial cross-correlation analysis (DPCCA) originally introduced by Yuan et al. (2015). In the DPCCA procedure, for each of the data series $x_{i}^{1}$, $x_{i}^{2}$, …, $x_{i}^{m}$ the so-called “profiles” are obtained as cumulative sums $X_{k}^{j}\equiv \overset{i}{\underset{k=1}{\sum }}x_{k}$, where *j* = 1, …, *m* is the series number, while *i* = 1, …, *N* is the data sample, and *N* is the length of the data. Next, the profiles are split into *K*_*s*_ windows of length *s*, and in each window the least mean squares polynomial fits $p_{\nu ,i}^{j}$ are calculated. By subtracting the polynomial fits $Y_{\left(i-1\right)\left(s+1\right)+k-i+1}^{j}=X_{k}^{j}-p_{k,i}^{j}$ the residual series $Y_{l}^{j}$, *l* = 1, …, (*N*−*s*)(*s*+1) are calculated. By calculating pairwise covariance between the residuals


(1) $$F_{j_{1},j_{2}}^{2}(s)\equiv \frac{\sum_{l=1}^{\left(N-s)(s+1\right)}Y_{l}^{j_{1}}Y_{l}^{j_{2}}}{\left(N-s)(s+1\right)}$$

for all *j*_1_, *j*_2_ = 1, …, *m* one obtains the covariance matrix


(2) $$\begin{array}{l} F^{2}\left(s\right)=\left[\begin{array}{cccc} F_{1,1}^{2}\left(s\right) & F_{1,2}^{2}\left(s\right) & \ldots & F_{1,m}^{2}\left(s\right)\\ F_{2,1}^{2}\left(s\right) & F_{2,2}^{2}\left(s\right) & \ldots & F_{2,m}^{2}\left(s\right)\\ \ldots & \ldots & \ldots & \ldots \\ F_{m,1}^{2}\left(s\right) & F_{m,2}^{2}\left(s\right) & \ldots & F_{m,m}^{2}\left(s\right) \end{array}\right]. \end{array}$$

Of note, the diagonal elements of the matrix *F*^2^(*s*) are simple variances and thus correspond to the fluctuation functions *F*(*s*) in the conventional detrended fluctuation analysis (DFA) proposed by Peng et al. (1994). It is known that for long-term correlated data the DFA fluctuation functions increase by a power law *F*(*s*)∝*s*^*H*^, where *H* is the Hurst exponent, irrespective of the order of the detrending polynomial. In the simple case of fully random (“white noise”) increments *H* = 1/2, while *H*>1/2 correspond to positively and *H* < 1/2 to negatively correlated increments, respectively.

In the following, *F*^2^(*s*) are normalized as


(3) $$\begin{array}{l} R_{j_{1},j_{2}}\left(s\right)\equiv \frac{F_{j_{1},j_{2}}^{2}\left(s\right)}{F_{j_{1},j_{1}}^{2}\left(s\right)\cdot F_{j_{2},j_{2}}^{2}\left(s\right)} \end{array}$$

to obtain the matrix of cross-correlation coefficients


(4) $$\begin{array}{l} R\left(s\right)=\left[\begin{array}{cccc} R_{1,1}\left(s\right) & R_{1,2}\left(s\right) & \ldots & R_{1,m}\left(s\right)\\ R_{2,1}\left(s\right) & R_{2,2}\left(s\right) & \ldots & R_{2,m}\left(s\right)\\ \ldots & \ldots & \ldots & \ldots \\ R_{m,1}\left(s\right) & R_{m,2}\left(s\right) & \ldots & R_{m,m}\left(s\right) \end{array}\right], \end{array}$$

where *R*_*j*_1_, *j*_2__ = 1, for all *j*_1_ = *j*_2_.

Next to exclude spurious correlations induced by cross-modulation of data series, one can also obtain partial correlation coefficients by calculating the inverse of the cross-correlation coefficient matrix


(5) $$\begin{array}{l} C\left(s\right)=R^{-1}\left(s\right)=\left[\begin{array}{cccc} C_{1,1}\left(s\right) & C_{1,2}\left(s\right) & \ldots & C_{1,m}\left(s\right)\\ C_{2,1}\left(s\right) & C_{2,2}\left(s\right) & \ldots & C_{2,m}\left(s\right)\\ \ldots & \ldots & \ldots & \ldots \\ C_{m,1}\left(s\right) & C_{m,2}\left(s\right) & \ldots & C_{m,m}\left(s\right) \end{array}\right] \end{array}$$

followed by its normalization as


(6) $$\begin{array}{l} P_{j_{1},j_{2}}\left(s\right)=\frac{-C_{j_{1},j_{2}}\left(s\right)}{\sqrt{C_{j_{1},j_{1}}\left(s\right)\cdot C_{j_{2},j_{2}}\left(s\right)}}, \end{array}$$

where the latter coefficients characterize intrinsic correlations between data series *j*_1_ and *j*_2_ (Baba et al., 2004; Yuan et al., 2015).

For an overall characterization of the animal movement model, we additionally modified the above method by replacement of the covariance coefficient in Equation (1) by the cross-covariance function calculated as


(7) $$\begin{array}{l} F_{j_{1},j_{2}}^{2}(s,k)\equiv \frac{\sum_{l=1}^{\left(N-s)(s+1\right)}Y_{l-k}^{j_{2}}Y_{l}^{j_{2}}}{\left(N-s)(s+1\right)}\text{or}\\ F_{j_{1},j_{2}}^{2}(s,k)\equiv \frac{\sum_{l=1}^{\left(N-s)(s+1\right)}Y_{l}^{j_{2}}Y_{l-k}^{j_{2}}}{\left(N-s)(s+1\right)}, \end{array}$$

where the additional index *k* ≤ *s*/2 implies the delay of either $Y^{j_{1}}$ or $Y^{j_{2}}$ by *k* samples, respectively. Algorithmically, the synchronously recorded data in the numerator of Equation (1) has been substituted by a series of relatively shifted copies followed by finding the position where the maximum cross-covariance can be observed. In addition, the total squared displacement was calculated as the sum of the squared displacements of projections on the X- and Y-axes, respectively. Finally, the value of the maximum of the cross-covariance function characterizes the coupling strength, while the position of the maximum determines the relative delay between $Y^{j_{1}}$ or $Y^{j_{2}}$ for each considered scale *s*. The above results are presented in the form of a directed graph, where directions of the edges are determined by the positions of the maxima, and further quantified by the coupling strength *P*_*ij*_ and delay *T*_*ij*_.

### 2.6. Statistical analysis

For the outliers removal, we employed the Tukey fence method based on the analysis of the interquartile range which is known for its resistance to the presence of extreme values and applicability to both normal and slightly skewed distributions. Distributions of movement metrics within the groups in many cases differed significantly from Gaussian distributions, as indicated by Kolmogorov-Smirnov and Shapiro-Wilk tests. Thus, for the assessment of the statistical significance of our results, we employed Mann-Whitney *U*-test for pairwise comparisons.

## 3. Results

**Figure 2:**
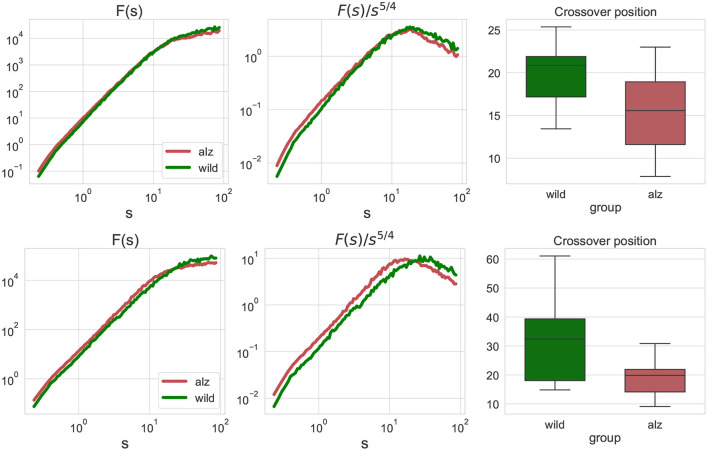
**Left:** fluctuation functions *F*_*j, j*_(*s*) for the movement trajectories along the X-axis **(upper)** and Y-axis **(lower)** for all animal body parts *j* as denoted in Figure 1 obtained by detrended fluctuation analysis (DFA). The green curve denotes wild type mice (control group), while the red curve denote the Alzheimer mice cohort (test group). Fluctuation functions scale asymptotically as *F*(*s*)∝*s*^2^ at small scales, and as *F*(*s*)∝*s*^1/2^ at large scales. **Middle:** show the same fluctuation functions *F*(*s*) divided by *s*^5/4^ used to determine the crossover position at the maximum of the rotated fluctuation function. **Right:** boxplots characterizing the crossover position statistics for all individual movement trajectories of each body part of each animal. Scales and crossover positions are expressed in seconds.

First, we performed the fluctuation analysis as indicated in Equations (1), (2) and obtained the fluctuation functions summarized in Figure 2 for the animal body parts movement projections on the X and Y axes, respectively (only group averages are shown). The figures indicate that fluctuation functions are characterized by two asymptotic regimes. At small scales, the fluctuation function after *s* steps increases algebraically approximately as *F*(*s*)∝*s*^2^, that is equivalent to the observation of 1/*f* noise in the movement increments. In contrast, at large scales the fluctuation function increases approximately as *F*(*s*)∝*s*^1/2^, indicating nearly random displacements (although strongly anti-persistent increments).

To determine the position of the maxima, fluctuation functions *F*(*s*) have been divided by *s*^5/4^ to achieve the same rate of decay on both sides of the crossover, in order to determine the position of the crossover at the maximum of the rotated fluctuation function. While the figure shows only the group averages, similar transformations have been applied to the fluctuations functions for all individual movement trajectories of each body part of each animal, and statistical significance of the observed shift in the crossover position has been explicitly validated using the Mann-Whitney *U*-test (*p* = 2.4 × 10^−7^ for the movements along the X-axis, and *p* = 4.8 × 10^−9^ for the movements along the Y-axis, respectively, that is well below the null hypothesis rejection threshold at 0.95 confidence level, also with multiple testing correction).

**Figure 3:**
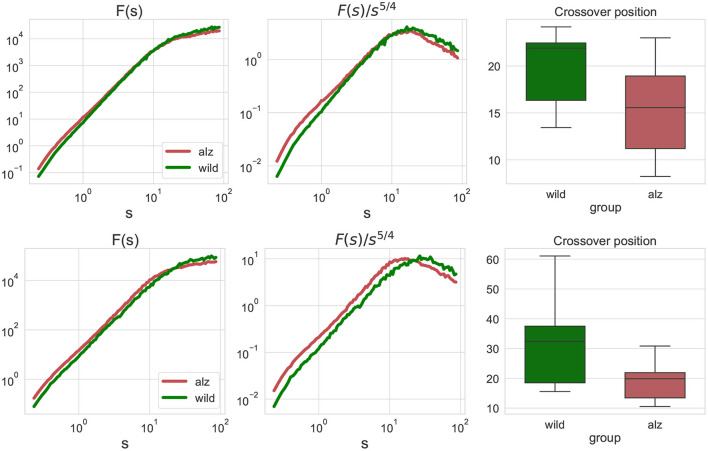
Similar fluctuation functions *F*_*j*_1_, *j*_2__(*s*) as Figure 2 but for all pairwise combinations of movement trajectories for different body parts *j*_1_≠*j*_2_ obtained by detrended cross-correlation analysis (DCCA). Scales and crossover positions are expressed in seconds.

The conventional fluctuation functions shown in Figure 3 characterize the dynamics of individual body parts, similar to those recently used to characterized the whole-animal movements of rats *R. norvegicus* in an open field test (Lyanova et al., 2022) and of fishes *D. rerio* in a novel tank test (Bogachev et al., 2023), respectively. In this work, we additionally considered joint fluctuation functions that characterize mutual interactions of different body parts obtained by detrended cross-correlation analysis (DCCA), indicated in Figure 3. Similarly, significant discrepancies between the crossover locations could be observed and explicitly supported by the Mann-Whitney *U*-test (*p* = 1.4 × 10^−7^ for the movements along the X-axis, and *p* = 2 × 10^−10^ for the movements along the Y-axis, respectively, that is well below the null hypothesis rejection threshold at 0.95 confidence level, also with multiple testing correction).

**Figure 4:**
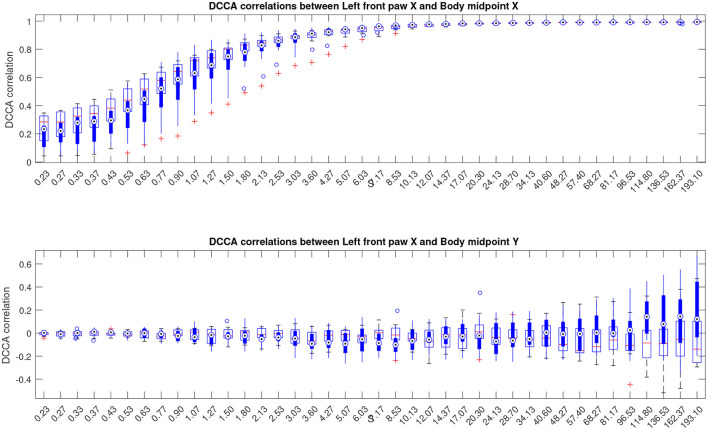
Boxplots indicating correlation dynamics of the left front paw (acting as a representative example) along the X-axis *relative to the animal body midpoint* as a function of scale *S* obtained by detrended cross-correlation analysis (DCCA). The upper panel shows correlations with midbody movements along the same X-axis, representing specific correlations, while the lower panel shows correlations with midbody movements along the orthogonal Y-axis that one could expect to be unrelated, at least to a certain approximation, and thus indicating the level of unspecific correlations. More similar figures for other body parts are presented in the Supplementary material. Results for animals with Alzheimer's disease are provided by filled boxes and blue circles for outliers; results for wild type animals (control group) are provided with open boxes and red plus signs for outliers.

Next, for a more detailed pairwise comparisons between the movement patterns, in addition to the comparison of the fluctuation functions, we calculated the cross-correlation matrices as indicated in Equations (3), (4) for each animal at different scales *s* and performed pairwise comparisons of the respective cross-correlation coefficients between the Alzheimer test group and the control group of animals summarized in Figure 4, see also Supplementary material for similar results for other animal body parts.

**Figure 5:**
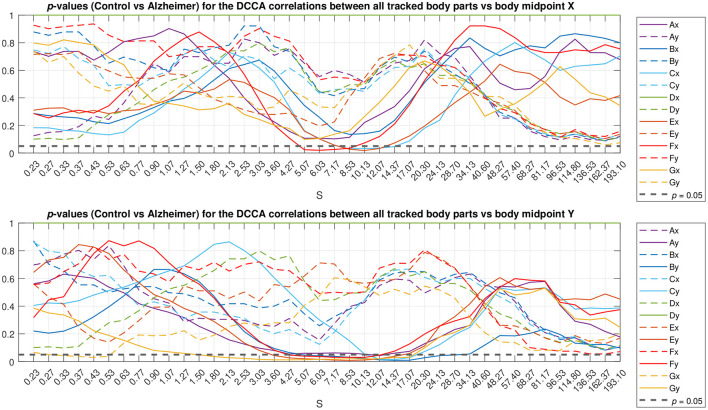
Scale localization of discrepancies between the correlation dynamics of the same animal body parts as denoted in Figure 1 *relative to the animal body midpoint* as a function of scale *S*, expressed in terms of *p*-values. Full lines denote specific relations corresponding to the same Cartesian coordinate (movements either along the X-axis in the upper panel or along the Y-axis in the lower panel), while dashed lines denote unspecific relations (either respective body part movements along the X-axis vs. the animal midpoint movements along the Y-axis, or vice versa). The horizontal dashed black line represents *p* < 0.05 level.

To localize typical scales where discrepancies between the gait patterns in the studied animal groups can be observed, we applied the Mann-Whitney *U*-test. The resulting *p*-values are summarized in Figure 5 as a function of scale *s*. The figure shows that most pronounced discrepancies can be observed typically at intermediate scales *s*≈5…10 s where they remain at *p* < 0.05 for the overwhelming majority of the tracked body parts. Remarkably, these discrepancies also appear most pronounced at scales nearing the position of the crossover, and thus could be likely associated with the shift in the crossover locations (that in turn are known to be shifted toward larger scales several-fold for both DFA and DCCA methods) for the respective animal body part movements between animals from the test and control groups, respectively.

**Figure 6:**
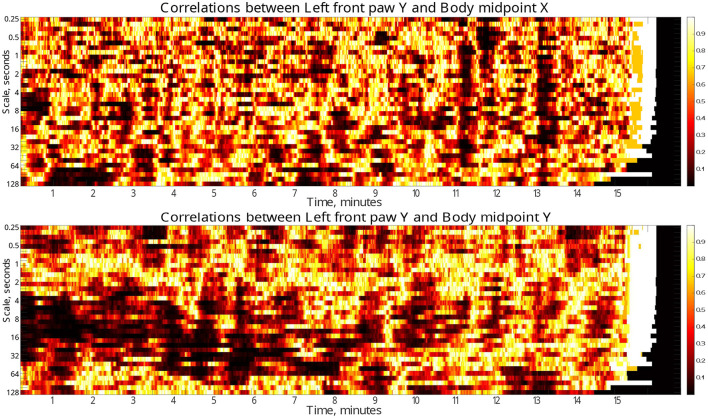
Time-scale localization of discrepancies (*p*-values) in the correlation dynamics of the left front paw (acting as a representative example) along the Y-axis *relative to the animal body midpoint* as a function of scale *S* between the test and the control animal groups. Compact dark areas indicate typical localization in the time-scale space where discrepancies can be observed. Discrepancies in the correlations with the animal body midpoint movements along the same Y-axis indicate presumable localization of discrepancies due to specific correlation patterns, while correlations with animal body midpoint movements along the orthogonal X-axis that one could expect to be unrelated, at least to a certain approximation, indicate the level of discrepancies caused by unspecific correlations.

Finally, to localize the respective discrepancies not only in the scale range, but also in time, we performed similar analysis in a gliding window of 30 s duration. Figure 6 (see also similar results for other body parts in the Supplementary material) indicate the dynamical evolution of the respective *p*-values by heatmaps presented in time-scale coordinates. The figures show that the majority of pronounced discrepancies exhibit two typical localizations.

In the first scenario, they localize around characteristic scales attributable to the respective fluctuation function crossover positions, while covering significant time spans (for a prominent example see Figure 5, see also Supplementary material for additional examples for other animal body parts). These discrepancies likely make the decisive contribution to the overall discrepancies reported in Figure 6. Remarkably, the above scenario can be observed predominantly in the first half of the test duration, and thus could be presumably associated with different adaptability to the environment in test and control animals, respectively.

In the second scenario, there are also discrepancies that localize in short time fragments only, while spanning over broad scale range, being observed predominantly in the second half of the test duration, that could be presumably associated with certain exploratory activities that are differently represented in the test and control animals, respectively.

Next in order to eliminate systematic correlation effects associated with the whole animal body movements from the interactions between individual animal body part movement patterns, we calculate partial correlations according to Equations (5), (6). In contrast to the conventional correlation metrics, partial correlations reveal the intrinsic correlations in each pairwise combination, by excluding contributions from other body parts. For example, at large scales the major contributor to the conventional correlation metrics is the animal walking trajectory, since all body parts follow the animal on its way. This can be observed explicitly in the gradual enhancement of the correlation coefficients with increasing scale, eventually converging to one at very large scales, where individual body movements relative to the animal body midpoint are very small compared to the total distance traveled by the animal over long time spans. Importantly, this effect is neither eliminated nor reduced by detrending procedures that compensate only trends represented by walking trajectories of particular animals, while do not help to “detach” the relative body part movements from the overall walking trajectory.

The latter could be potentially resolved by considering a two-level cascade model, with the first level representing the animal walking trajectory (e.g., at the body midpoint), and the second level representing animal body part movements relative to the body midpoint. The above scenario can be represented by the so-called superstatistical models (Beck and Cohen, 2003; Beck et al., 2005) that have been recently applied in the context of various natural complex systems ranging from climate and weather to information flow dynamics, as well as DNA and protein structures (Bogachev et al., 2016, 2017; Tamazian et al., 2016; Markelov et al., 2017; Itto and Beck, 2021; Schäfer et al., 2021).

**Figure 7:**
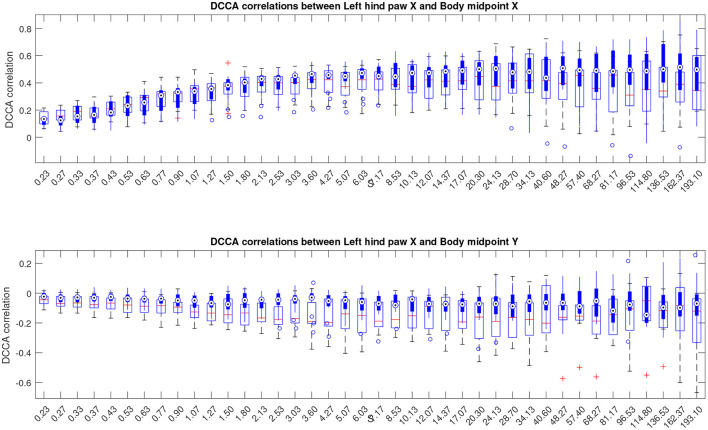
Boxplots indicating partial cross-correlations of the left hind paw along the X-axis *relative to the animal body midpoint* as a function of scale *S* obtained by detrended partial cross-correlation analysis (DPCCA). The upper panel shows correlations with midbody movements along the same X-axis, representing specific correlations, while the lower panel shows correlations with midbody movements along the orthogonal Y-axis that one could expect to be unrelated, at least to a certain approximation, and thus indicating the level of unspecific correlations. More similar figures for other body parts are presented in the Supplementary material. Results for animals with Alzheimer's disease are provided by filled boxes and blue circles for outliers; results for wild type animals (control group) are provided with open boxes and red plus signs for outliers.

In this work, we follow a slightly different route, and consider partial correlations calculated as indicated in Equations (5), (6). The results are depicted in Figure 7, see also similar results for other animal body parts in the Supplementary material. The figures show that the above mentioned convergence to nearly unit correlations at large scales can no longer be observed, and thus the scale dependent trend has been eliminated from the correlation pattern by separation of the overall contributions into pairwise correlations that appear complementary to each other.

**Figure 8:**
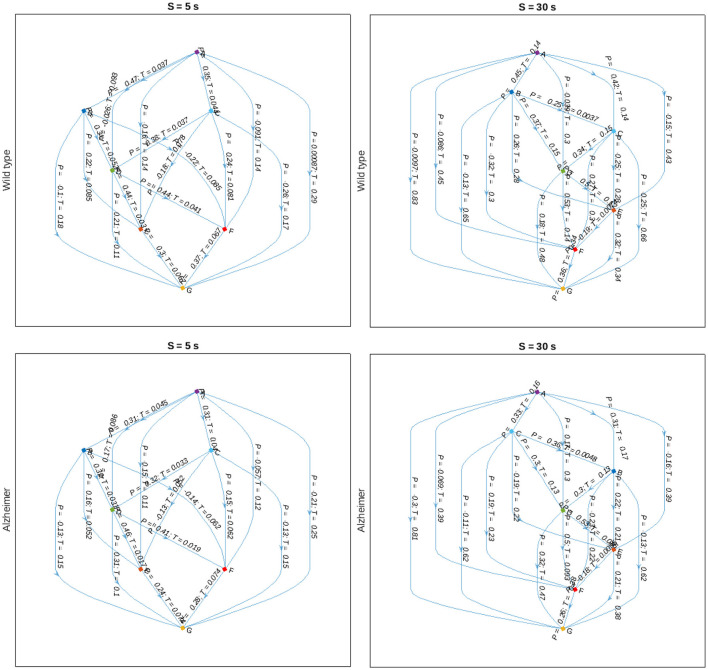
Graph-based representations of maximum partial correlations *P*_*ij*_ between animal body part movements observed at scales *S* = 5 and 30 s **(left, right)** and delays *T*_*ij*_ corresponding to the positions of the respective cross-covariance maxima for the control and Alzheimer's disease animal groups **(upper, lower)**, respectively.

Finally, we reconstruct the animal body part interaction network using partial correlation analysis with pairwise alignments to adjust for maximum cross-correlations according to Equation (7), and represent the results in the form of a directed graph indicated in Figure 8. In this graph, nodes are attributed to various animal body parts, while edges characterize partial correlations at the positions of the maximum covariance between them, as well as corresponding delays. The figure indicates that there are typically shorter time delays between body part movements in the animals with Alzheimer's disease compared to the control group, that could be observed at different scales. Although no statistically significant discrepancies could be observed for individual pairwise delays, most likely due to small sample sizes and considerable within-group variations, a certain tendency could be observed at various scales, also beyond the two examples shown in Figure 8.

## 4. Discussion

Statistical analysis of walking patterns has a long history in applied mathematics and especially statistical physics. The most basic concept that dates nearly two centuries back is the simple Brownian motion, that also represents the limiting case for the random walk model. Under the assumption of statistically independent and identically distributed increments at each step, the root-mean square displacement of the random walker from the origin after *s* steps, according to the classic Fick's diffusion law, scales as *F*(*s*)∝*s*^1/2^. Possible generalizations of this concept for correlated increments include the fBm model with *F*(*s*)∝*s*^*H*^, a widely used approach for modeling data series with long-term persistence, including animal movement patterns in their natural environments. The above laws hold asymptotically, making these models completely scale-free, characterized by theoretically unlimited long-term correlations.

In contrast, experimental animal walking patterns, including the open field test, premise that the motions are limited to a certain confined space, making asymptotically scale-free models hardly adequate, due to a breakdown of persistence above a certain scale. Moreover, the alternating movement patterns that are inevitable in confined space due to imminent reversals also assume the emergence of anti-persistence at large scales. Accordingly, the resulting animal movement model is no longer expected to exhibit scale-free properties, but rather consists of at least two characteristic regimes, represented by a persistent random walk at small scales only, and substituted by more random or even anti-persistent patterns with increasing scale.

In a recent study of the whole-animal movements we have characterized the behavioral patterns of *R. norvegicus* in a rodent open field test (Lyanova et al., 2022) and for *D. rerio* in a novel tank test (Bogachev et al., 2023). In the above studies, we have already observed two asymptotic scaling regimes separated by a characteristic crossover *s*_×_ that also appeared a single characteristic scale parameter. We have also noted that the crossover location that represents the only free parameter in the animal movement model explicitly reflected behavioral alterations in the presence of various stimulative and sedative pharmacological stimuli. Our current results are closely reminiscent to those previously observed, with similar correlation exponents *H*≈2 observed at small scales, although smaller correlation exponents close or slightly above *H*≈1/2 observed at large scales, in contrast to *H*≈1 estimated in previous studies (Lyanova et al., 2022; Bogachev et al., 2023). While there is no obvious reason for the latter discrepancy, there could be potentially multiple contributing effects, ranging from finite size effects associated with the test recording duration to differences in video analysis and data pre-processing (e.g., smoothing for background noise reduction) algorithms.

In general, oscillatory dynamics are known to be reflected by localized pulses in the DFA fluctuation functions, as it has been shown earlier, for example, by Ludescher et al. (2011), Hardstone et al. (2012), and Govindan et al. (2017) and other studies. In the case of animal walking patterns, there are at least two characteristic oscillations, including (i) short-term locomotor dynamics localized around a single step scale, and (ii) much slower and often behaviorally driven alterations in the locomotor activity patterns, such as changes of walking direction, start/stop events, rearing etc. According to a recent model based study by Ludescher et al. (2011), the observed crossover in the second-order DFA fluctuation functions is typically located close to the oscillation period or slightly shifted toward large scales (see Figures 3C, 8C, 9C in Ludescher et al., 2011). In our study the typical crossover position has been observed at scales of 10 s and above that, thus more likely attributable to the component (ii).

There are several other indicators supporting contribution of behavioral aspects to the emergence and location of the crossover, in particular, (a) observation of a similar crossover not only in body part movements, but also in the animal midpoint dynamics; (b) observation of a similar crossover in a recent work characterizing walking patterns in rats *R.norvegicus* where the open field have been observed from above, and thus no tracking of individual body parts have been performed (Lyanova et al., 2022), as well as (c) observation of a similar crossover in a recent work characterizing movement patterns in fishes *D.rerio* in a novel tank test unrelated to walking (although swimming patterns also contain oscillatory patterns) (Bogachev et al., 2023).

We believe that the shift in the crossover position observed in Figures 2, 3 reflects lower cadence, speed, and stride length altogether with other impairments in mice with Alzheimer's disease compared to the control group. In addition to the reduction of stride lengths, gait in the presence of Alzheimer's disease is characterized by shorter stride times, as manifested in the observed tendency of shorter time delays corresponding to maximum cross-covariances between animal body part movements in Figure 8, in general agreement with recent data obtained in animal experiments (Nyul-Toth et al., 2021). Moreover, an early crossover indicating less persistence could be interpreted as an indicator of generally more erratic movement patterns, presumably associated with higher gait variability, that has been previously observed in patients with Alzheimer's disease reported in recent literature (Cedervall et al., 2014; Pieruccini-Faria et al., 2021).

As a side methodological remark, it is also interesting to note that rather universal scaling laws in the multi-scale analysis of oscillatory dynamics in physiological systems could often be observed when considering scales not in the units of time, but rather in periods of a reference rhythm, such as the units of heartbeat intervals in Bogachev et al. (2009), although these laws exhibit a breakdown when rescaled in the units of time, indicating that multiple regulatory oscillations increased and reduced their characteristic rates together with the reference (e.g., heartbeat) rhythm. In the view of the above, we cannot exclude that the discrepancies between groups may become less significant if rescaled in the units of single steps (which in turn would require extraction of time stamps of every single animal step, and thus not easy to verify).

Here we suggested an extension of the above DFA-based methodology to the joint analysis of animal body parts movement patterns. For that, we first replaced the previously employed detrended fluctuation analysis (DFA) that is consistent with Equation (1) at *j*_1_ = *j*_2_, thus providing the diagonal of the fluctuation matrix in Equation (2), by the detrended cross-correlation analysis (DCCA) as indicated in Equations (1)–(4). Accordingly, the between group discrepancies in the gait variability patterns are explicitly reflected in the cross-correlation matrices indicated in Equation (4). More specifically, while both asymptotic regimes typically exhibit similar correlation patterns, discrepancies at intermediate scales are reflected in the alterations of the cross-correlation patterns, as it can be observed while comparing the control and the test animal groups, leading to significant discrepancies in the cross-correlation coefficients *R*_*i, j*_ in Equation (4).

However, as one can see from Figure 8, the above effect is largely hindered by the overall enhancement of the cross-correlations between animal body parts with increasing scale, originating from movement of animal body parts relative to the body midpoint becoming increasingly small and eventually nearly negligible compared to the total distance traveled by the animal over long time spans. Although the above effect does not prevent the statistical analysis from finding significant discrepancies between the cross-correlation patterns in the control and test groups, as indicated in Figure 2, largely due to the reduction of the respective within-group variances, the above effects are no longer clearly visualized.

To partially overcome the above issue, in the next step we further extend the analysis methodology to the detrended partial cross-correlation analysis (DPCCA) recently proposed by Yuan et al. (2015) and extract partial correlations as indicated in Equations (5), (6). The above transformations imply the extraction of the intrinsic correlations for each pair of animal body parts by the elimination of the contributions of the secondary correlation effects, such as, for example, detachment of the relative body part movements from the overall animal walking trajectory. As a result, the partial correlation matrix contains information on any particular mutual interaction only once. In turn, partial correlations appear complementary to each other, and thus the overall correlations could be potentially reconstructed from the partial correlations.

The latter implies that partial correlations represent a more appropriate quantity for the reconstruction of the complex animal behavior patterns from multi-scale movement analysis data. Indeed, the most intuitive way to simulate the animal movement patterns would follow a somewhat hierarchical algorithm, starting with the reconstruction of the overall animal walking pattern on large scales, further complemented by specific gait characteristics at smaller scales, as it is commonly done in the framework of superstatistical models (Beck and Cohen, 2003; Beck et al., 2005; Bogachev et al., 2016, 2017; Tamazian et al., 2016; Markelov et al., 2017; Itto and Beck, 2021; Schäfer et al., 2021). A somewhat similar approach could be potentially based on the multi-scale partial cross-correlation data, which contains essential information about the relative movement characteristics of the animal body parts, while remaining invariant to particular trajectories traveled by the animals during tests, due to the detrending features of the algorithm.

Finally, in order to reconstruct the animal body part movement model including time delays, we employed an in-house developed modification of detrended partial cross-correlation analysis with pairwise alignments to adjust for maximum cross-correlations according to Equation (7), and represent the results in the form of a directed graph indicated in Figure 8. In this graph, nodes are attributed to various animal body parts, while edges characterize partial correlations at the positions of the maximum covariance between them, as well as corresponding delays. Since all secondary correlations have been already eliminated, the respective graphs represent the “backbone” of the animal body part interaction network. In turn, in order to reconstruct the full correlation patterns, at least in the first approximation, it should be sufficient to generate the correlations between the nodes represented by the “backbone” edges with corresponding time delays, eventually leading to the emergence of the remaining secondary correlations. Accordingly, the above model representation could potentially give rise to a correlation based approach to the animal movement reconstructions and their computer simulations at multiple scales by simply following the algorithm represented by a directed graph.

Remarkably, the above model representations are closely reminiscent to the recently emerged physiological networks representing multiple physiological processes in either animal or human body, typically obtained by different, although complementary measurement techniques, including cardiac, respiratory, locomotor, circadian and other activities and studying their interactions in the integrated physiologic system (Bartsch et al., 2012; Bashan et al., 2012), that very recently found applications in the diagnostics of Parkinson's disease (Asher et al., 2021; Fay-Karmon et al., 2021).

As a potential outlook, we would like to mention that the partial cross-correlations in the animal movement patterns with corresponding delays are capable of revealing the intrinsic interaction patterns, while the sign of the time shift can be used to determine causal relationships. Figure 8 indicates a simple example of such reconstruction, with the directions of the edges not predefined, but rather obtained automatically in a data-driven manner based on the signs of the respective delays reflected in the cross-covariance maxima locations, following the simple logic that the cause can only precede the response, and not vice versa. Based on the directional graph where edges are characterized not only by the measure of the strength of the interactions, but also by corresponding delays, which in turn determine the potential causal relationships, could be useful for the reconstruction of causal physiological networks (Günther et al., 2022). Furthermore, since partial correlations can be used to calculate conditional probabilities under certain constraints (Baba et al., 2004; Baba and Sibuya, 2005), the directed graph obtained this way would lead to a variant of a Bayesian network, a powerful tool widely used in the analysis of interventional studies and clinical investigations (Hanea et al., 2015).

As an obvious limitation of the above approach, we should mention that it captures only the dominant coupling in each pairwise interaction characterized by the maximum covariance, while there could be more local peaks that are simply ignored by this approach. In some cases, this situation can be partially compensated by the additive contribution of direct and indirect effects, for example, when movements of node D exhibits maximum correlation with node A with delay, that is different from the sum of delays in A-B-D or A-C-D chains. One possible approach to overcoming the above limitation could be based on the replacement of the correlation metric by some kind of multiple alignment procedure reminiscent to those widely used in the genetic sequence analysis (with appropriate restrictions taking into account known physical and physiological limitations) leading to a consensus alignment characterized by the maximum of the overall alignment score, like the multivariate variant of the dynamic time warping algorithm (Helwig et al., 2011; Bankó and Abonyi, 2012). In addition, other metrics alternative to linear correlations could be considered as the core of the algorithm, especially for systems with strong nonlinear interactions, with a possible combination of metrics that appear complementary (for a recent comparative study, we refer to Pyko et al. (2018) and references therein).

## Data availability statement

The datasets presented in this study can be found in online repositories. The names of the repository/repositories and accession number(s) can be found at: https://gitlab.com/digiratory/research/-/tree/main/Multiscale_analysis_of_AD_mouse_animal_behavior.

## Ethics statement

The animal study was reviewed and approved by Animal Care and Use Committee of the Federal Research Center Kazan Scientific Center of the Russian Academy of Sciences.

## Author contributions

MB, AS, DK, AK, and YM contributed to conception and design of the study. ED, TA, KP, and YM performed the animal experiments and obtained video recordings. KG and AL processed video recordings and extracted animal movement data. NP and MT performed the statistical analysis. MB, AS, KG, NP, AL, SP, TA, and YM wrote the first draft of the manuscript. All authors contributed to manuscript revision and approved the submitted version.
