# Supplementary material for: Video-based marker-free tracking and multi-scale analysis of mouse locomotor activity and behavioral aspects in an open field arena: A perspective approach to the quantification of complex gait disturbances associated with Alzheimer's disease
Source: Front Neuroinform. 2023 Feb 2;17:1101112. doi: 10.3389/fninf.2023.1101112 (PMC9932053; doi:10.3389/fninf.2023.1101112)
Supplement: Supplementary file 1 [file Data_Sheet_1.pdf]

# Supplementary Material

## SUPPLEMENTARY FIGURES

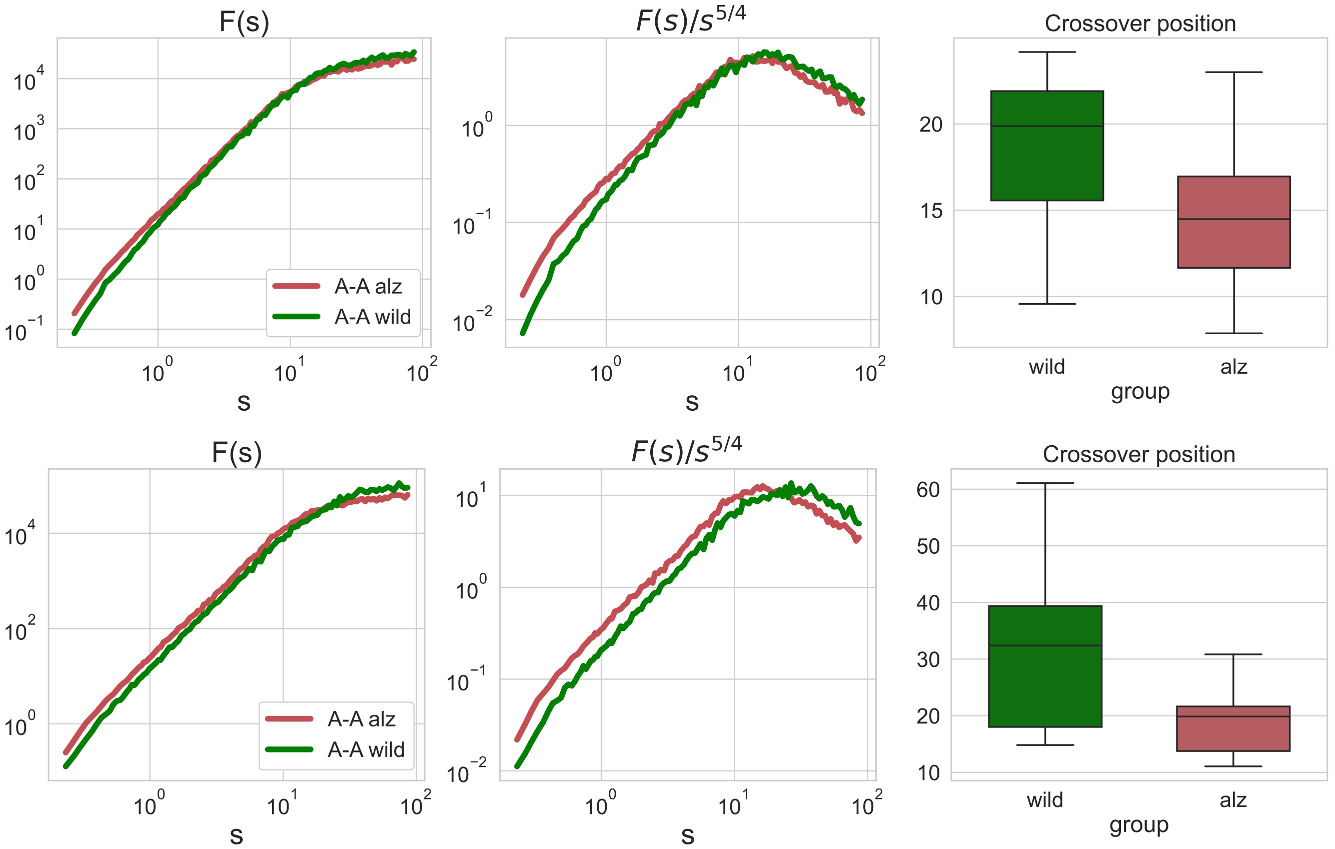


**Figure S1.** Left panels: fluctuation functions $F_{j,j}(s)$ for the movement trajectories of the snout along the X-axis (upper panels) and Y-axis (lower panels) obtained by detrended fluctuation analysis (DFA). The green curve denotes wild type mice (control group), while the red curve denote the Alzheimer mice cohort (test group). Fluctuation functions scale asymptotically as $F(s) \propto s^2$ at small scales, and as $F(s) \propto s^{1/2}$ at large scales. Middle panels show the same fluctuation functions $F(s)$ divided by $s^{5/4}$ used to determine the crossover position at the maximum of the rotated fluctuation function. Right panels: boxplots characterizing the crossover position statistics for all individual movement trajectories of each body part of each animal.

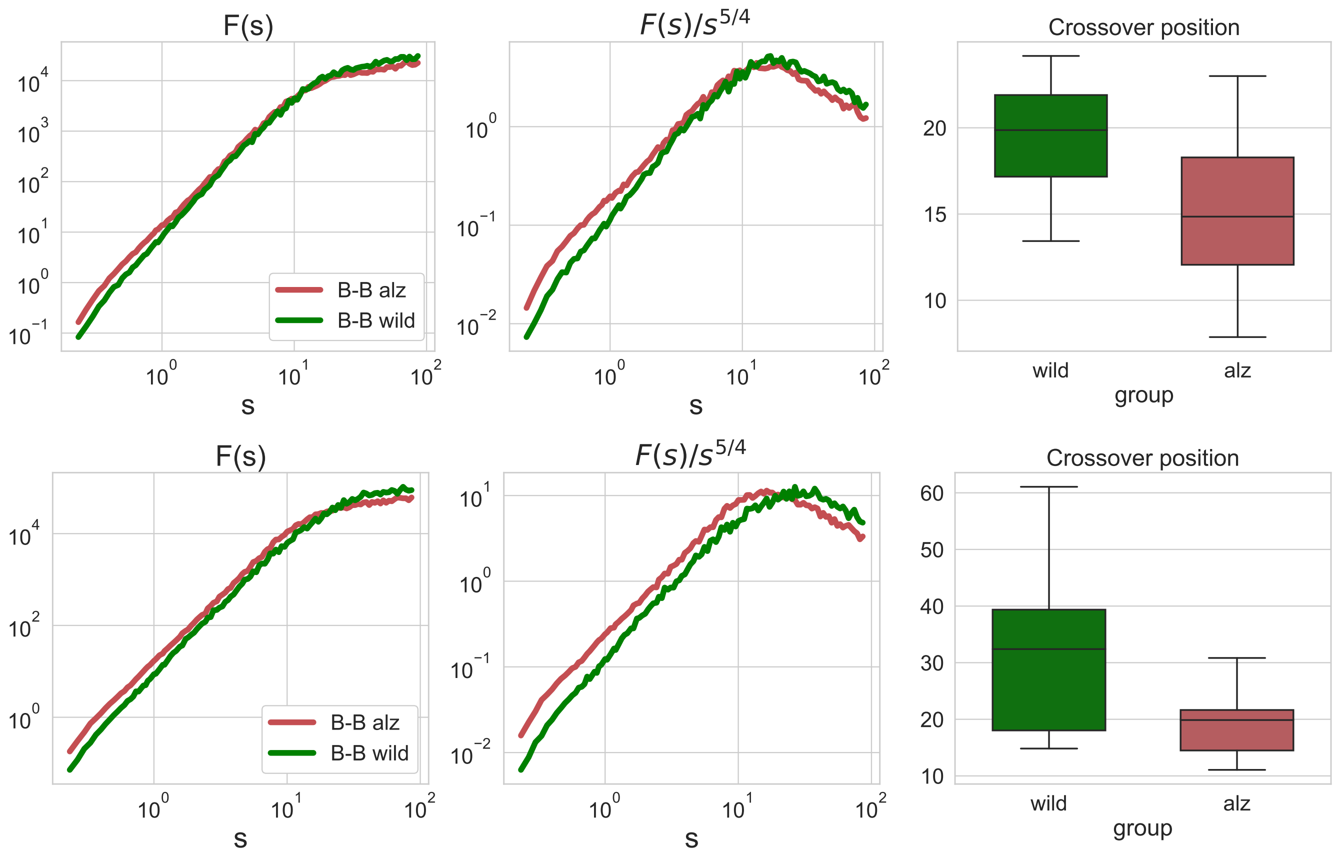


**Figure S2.** Left panels: fluctuation functions $F_{j,j}(s)$ for the movement trajectories of the left front paw along the X-axis (upper panels) and Y-axis (lower panels) obtained by detrended fluctuation analysis (DFA). The green curve denotes wild type mice (control group), while the red curve denote the Alzheimer mice cohort (test group). Fluctuation functions scale asymptotically as $F(s) \propto s^2$ at small scales, and as $F(s) \propto s^{1/2}$ at large scales. Middle panels show the same fluctuation functions $F(s)$ divided by $s^{5/4}$ used to determine the crossover position at the maximum of the rotated fluctuation function. Right panels: boxplots characterizing the crossover position statistics for all individual movement trajectories of each body part of each animal.

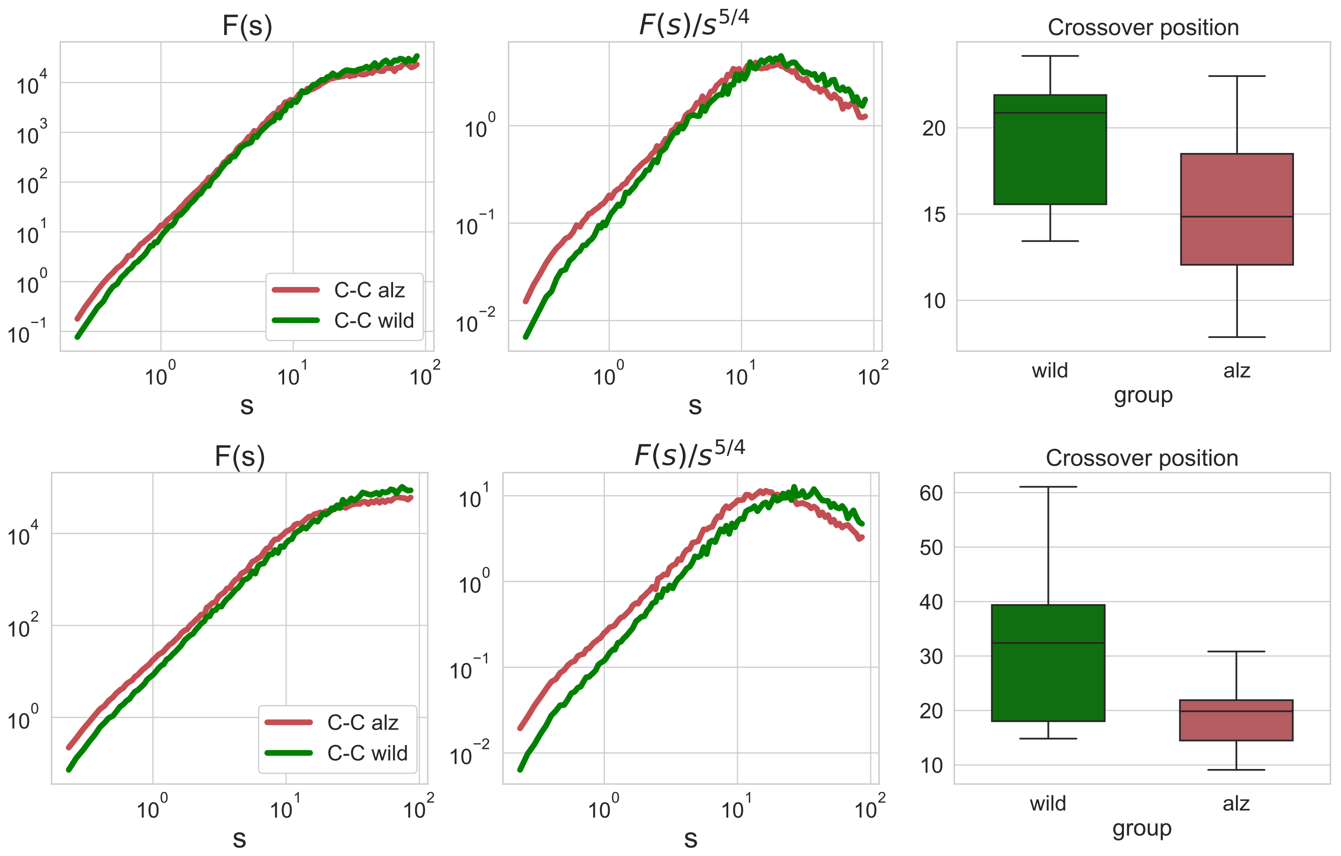


**Figure S3.** Left panels: fluctuation functions $F_{j,j}(s)$ for the movement trajectories of the right front paw along the X-axis (upper panels) and Y-axis (lower panels) obtained by detrended fluctuation analysis (DFA). The green curve denotes wild type mice (control group), while the red curve denote the Alzheimer mice cohort (test group). Fluctuation functions scale asymptotically as $F(s) \propto s^2$ at small scales, and as $F(s) \propto s^{1/2}$ at large scales. Middle panels show the same fluctuation functions $F(s)$ divided by $s^{5/4}$ used to determine the crossover position at the maximum of the rotated fluctuation function. Right panels: boxplots characterizing the crossover position statistics for all individual movement trajectories of each body part of each animal.

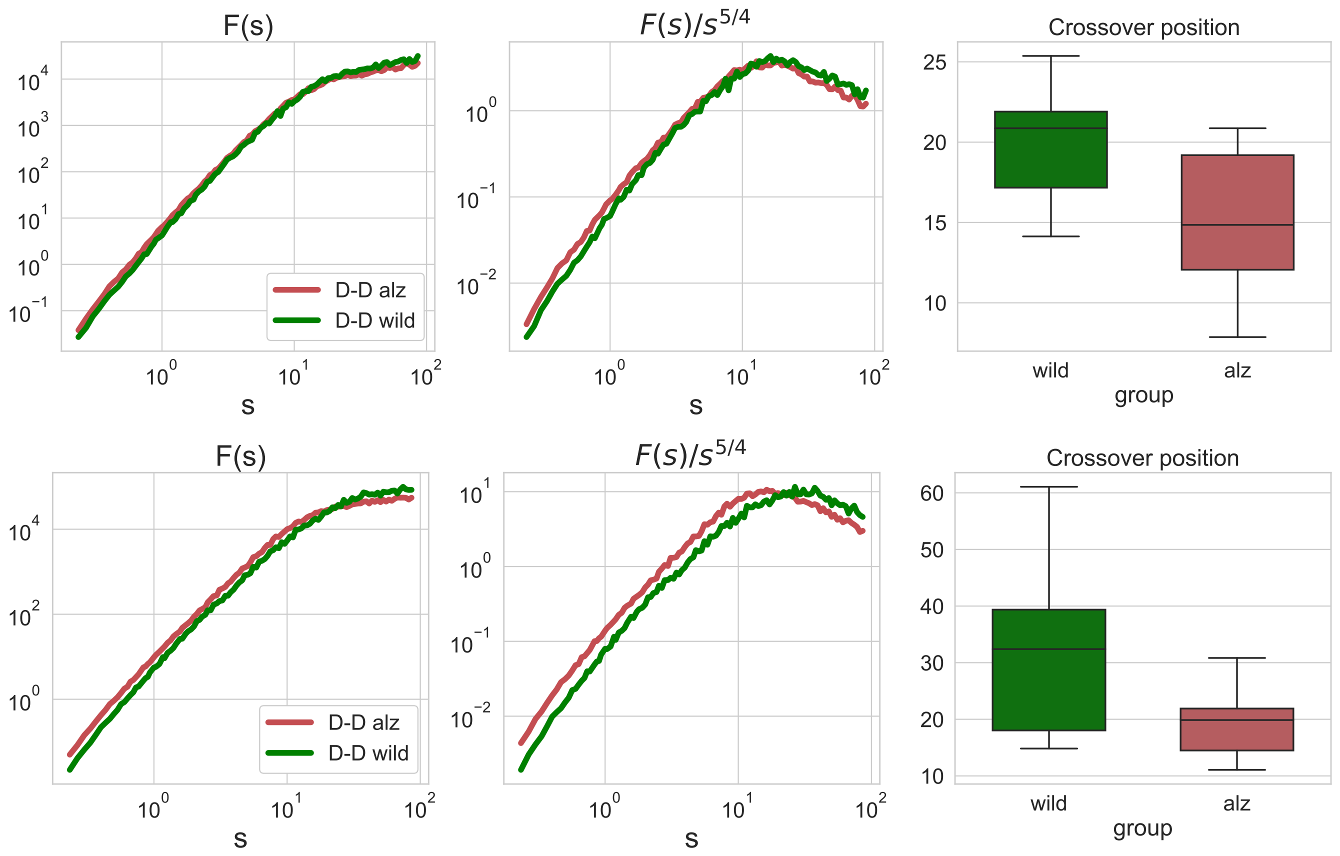


**Figure S4.** Left panels: fluctuation functions $F_{j,j}(s)$ for the movement trajectories of the animal body midpoint along the X-axis (upper panels) and Y-axis (lower panels) obtained by detrended fluctuation analysis (DFA). The green curve denotes wild type mice (control group), while the red curve denote the Alzheimer mice cohort (test group). Fluctuation functions scale asymptotically as $F(s) \propto s^2$ at small scales, and as $F(s) \propto s^{1/2}$ at large scales. Middle panels show the same fluctuation functions $F(s)$ divided by $s^{5/4}$ used to determine the crossover position at the maximum of the rotated fluctuation function. Right panels: boxplots characterizing the crossover position statistics for all individual movement trajectories of each body part of each animal.

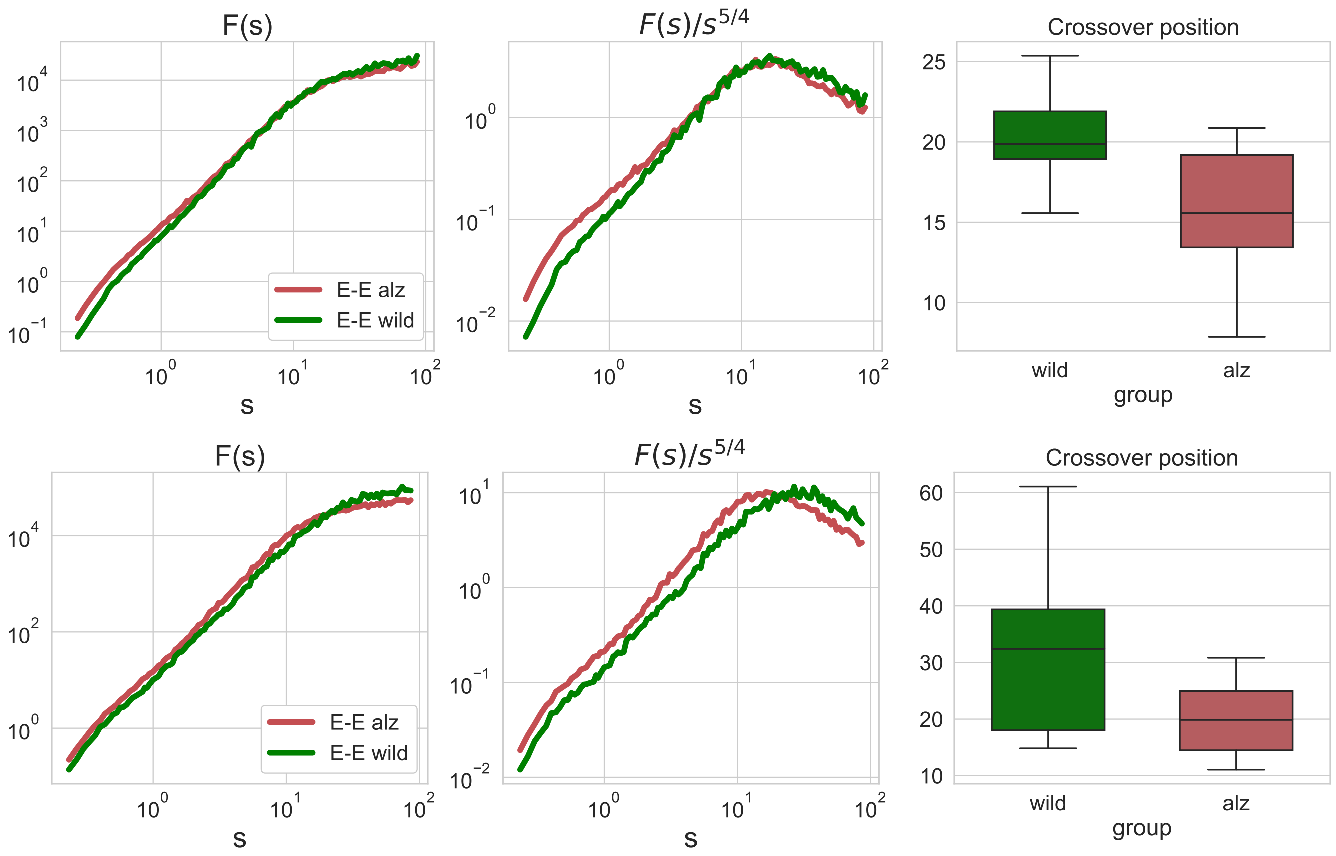


**Figure S5.** Left panels: fluctuation functions $F_{j,j}(s)$ for the movement trajectories of the left hind paw along the X-axis (upper panels) and Y-axis (lower panels) obtained by detrended fluctuation analysis (DFA). The green curve denotes wild type mice (control group), while the red curve denote the Alzheimer mice cohort (test group). Fluctuation functions scale asymptotically as $F(s) \propto s^2$ at small scales, and as $F(s) \propto s^{1/2}$ at large scales. Middle panels show the same fluctuation functions $F(s)$ divided by $s^{5/4}$ used to determine the crossover position at the maximum of the rotated fluctuation function. Right panels: boxplots characterizing the crossover position statistics for all individual movement trajectories of each body part of each animal.

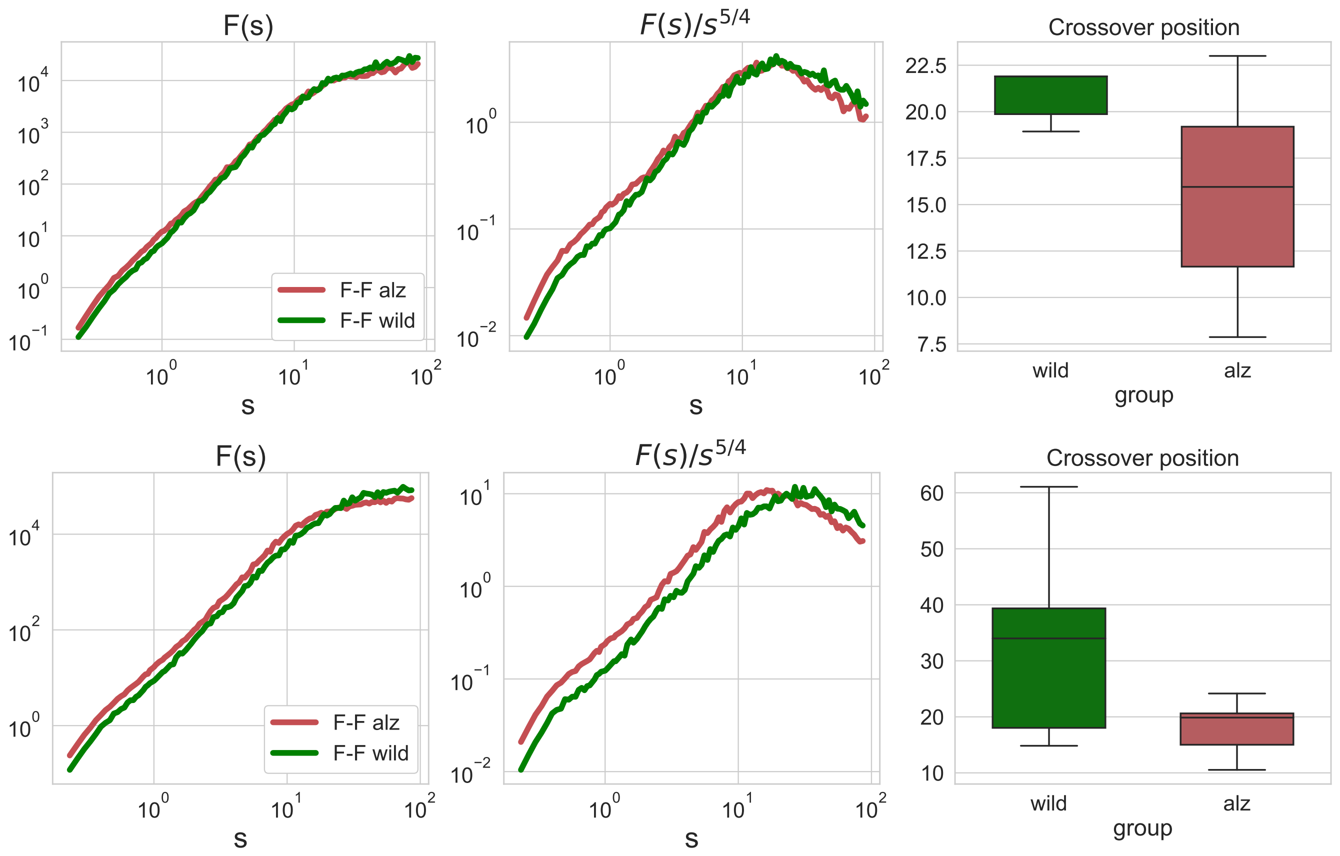


**Figure S6.** Left panels: fluctuation functions $F_{j,j}(s)$ for the movement trajectories of the right hind paw along the X-axis (upper panels) and Y-axis (lower panels) obtained by detrended fluctuation analysis (DFA). The green curve denotes wild type mice (control group), while the red curve denote the Alzheimer mice cohort (test group). Fluctuation functions scale asymptotically as $F(s) \propto s^2$ at small scales, and as $F(s) \propto s^{1/2}$ at large scales. Middle panels show the same fluctuation functions $F(s)$ divided by $s^{5/4}$ used to determine the crossover position at the maximum of the rotated fluctuation function. Right panels: boxplots characterizing the crossover position statistics for all individual movement trajectories of each body part of each animal.

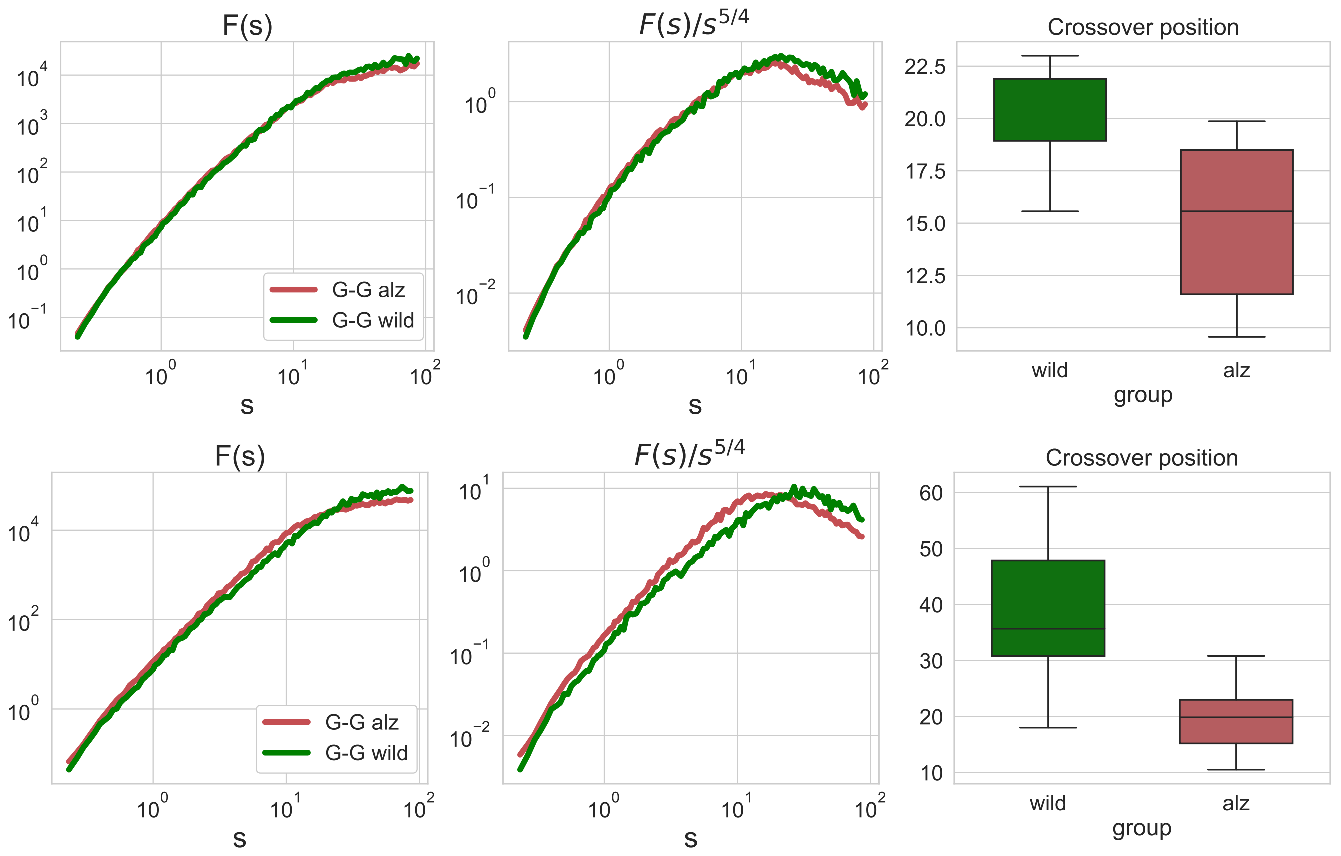


**Figure S7.** Left panels: fluctuation functions $F_{j,j}(s)$ for the movement trajectories of the tail along the X-axis (upper panels) and Y-axis (lower panels) obtained by detrended fluctuation analysis (DFA). The green curve denotes wild type mice (control group), while the red curve denote the Alzheimer mice cohort (test group). Fluctuation functions scale asymptotically as $F(s) \propto s^2$ at small scales, and as $F(s) \propto s^{1/2}$ at large scales. Middle panels show the same fluctuation functions $F(s)$ divided by $s^{5/4}$ used to determine the crossover position at the maximum of the rotated fluctuation function. Right panels: boxplots characterizing the crossover position statistics for all individual movement trajectories of each body part of each animal.

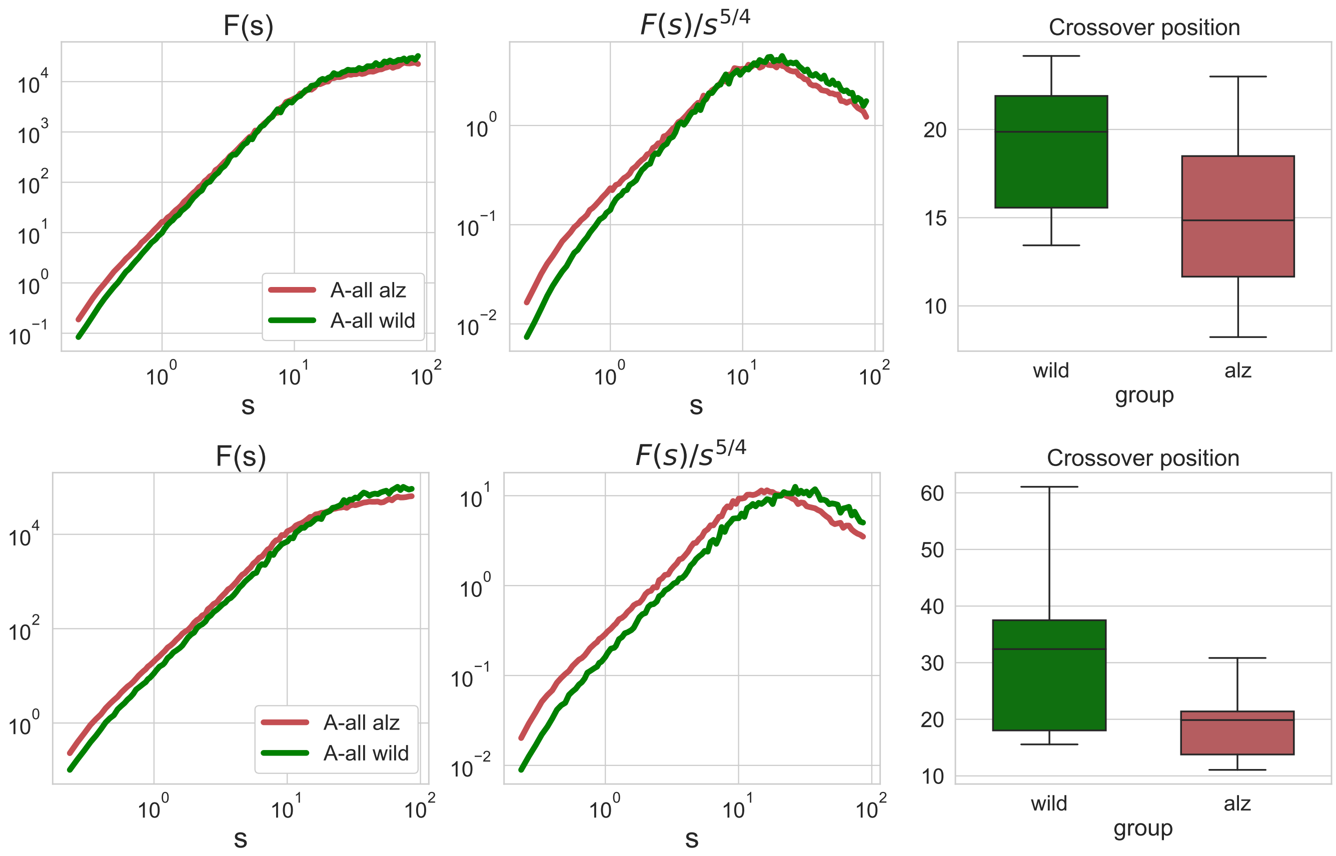


**Figure S8.** Similar fluctuation functions $F_{j_1,j_2}(s)$ as Fig. S1 for the relative movement trajectories between the snout and other body parts $j_1 \neq j_2$ obtained by detrended cross-correlation analysis (DCCA).

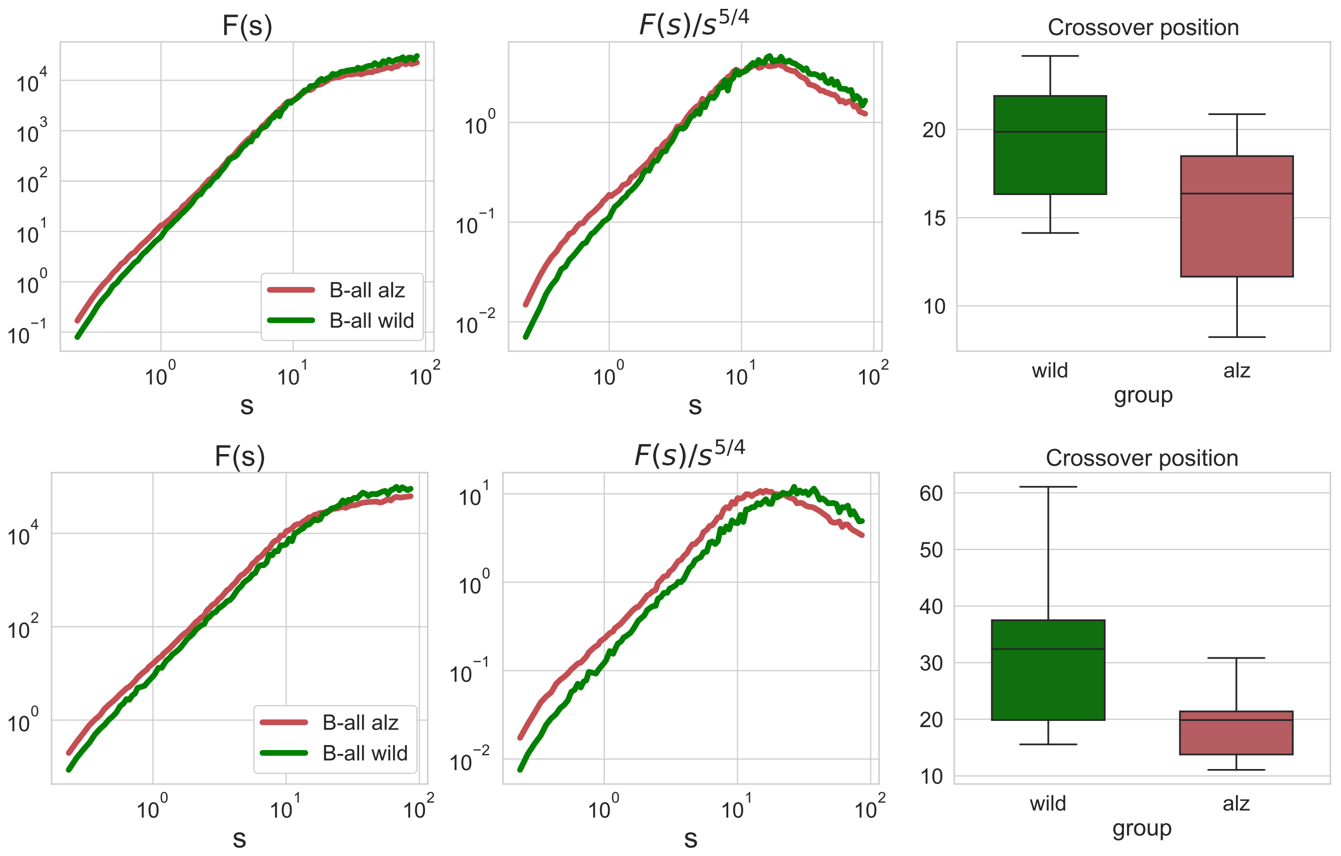


**Figure S9.** Similar fluctuation functions $F_{j_1,j_2}(s)$ as Fig. S2 for the relative movement trajectories between the left front paw and other body parts $j_1 \neq j_2$ obtained by detrended cross-correlation analysis (DCCA).

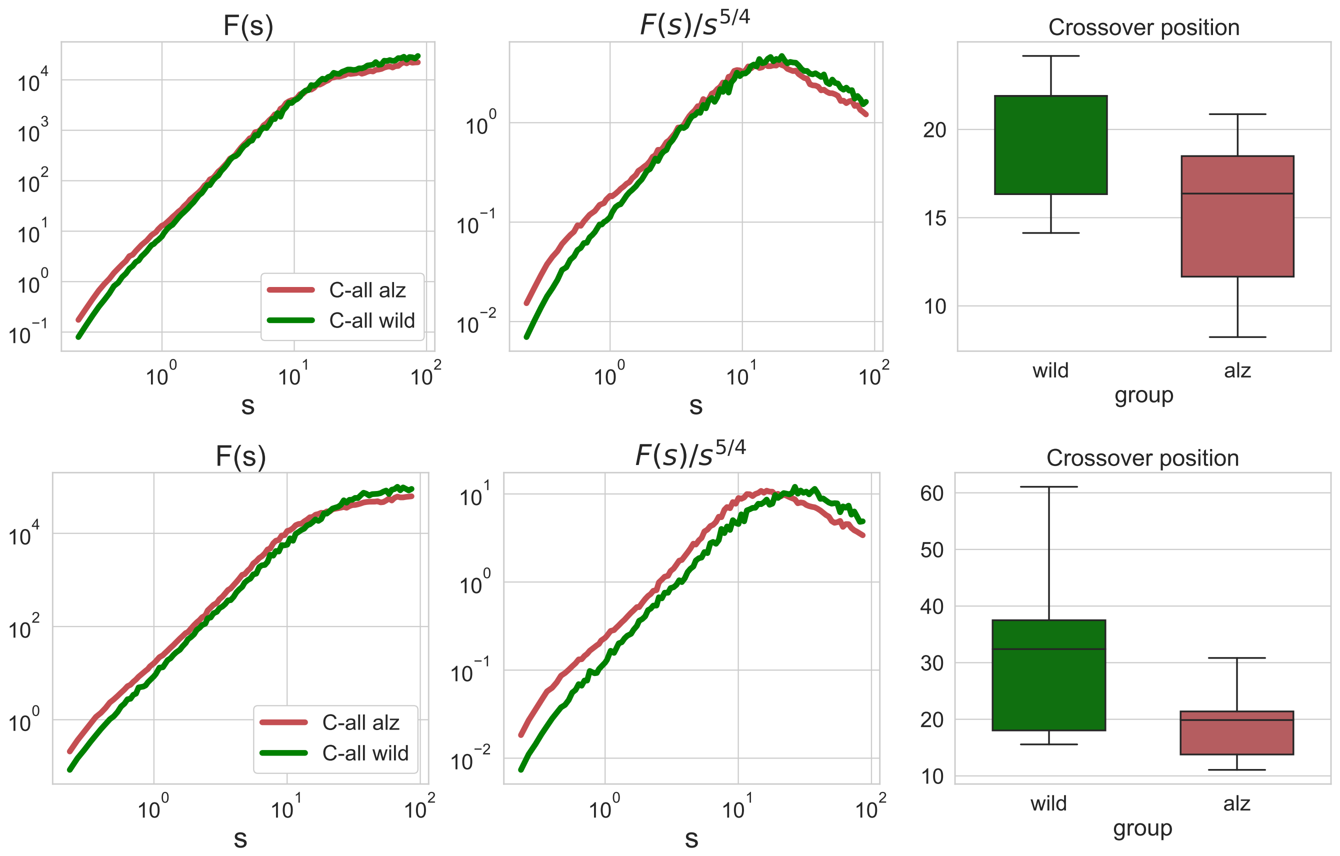


**Figure S10.** Similar fluctuation functions $F_{j_1,j_2}(s)$ as Fig. S3 for the relative movement trajectories between the right front paw and other body parts $j_1 \neq j_2$ obtained by detrended cross-correlation analysis (DCCA).

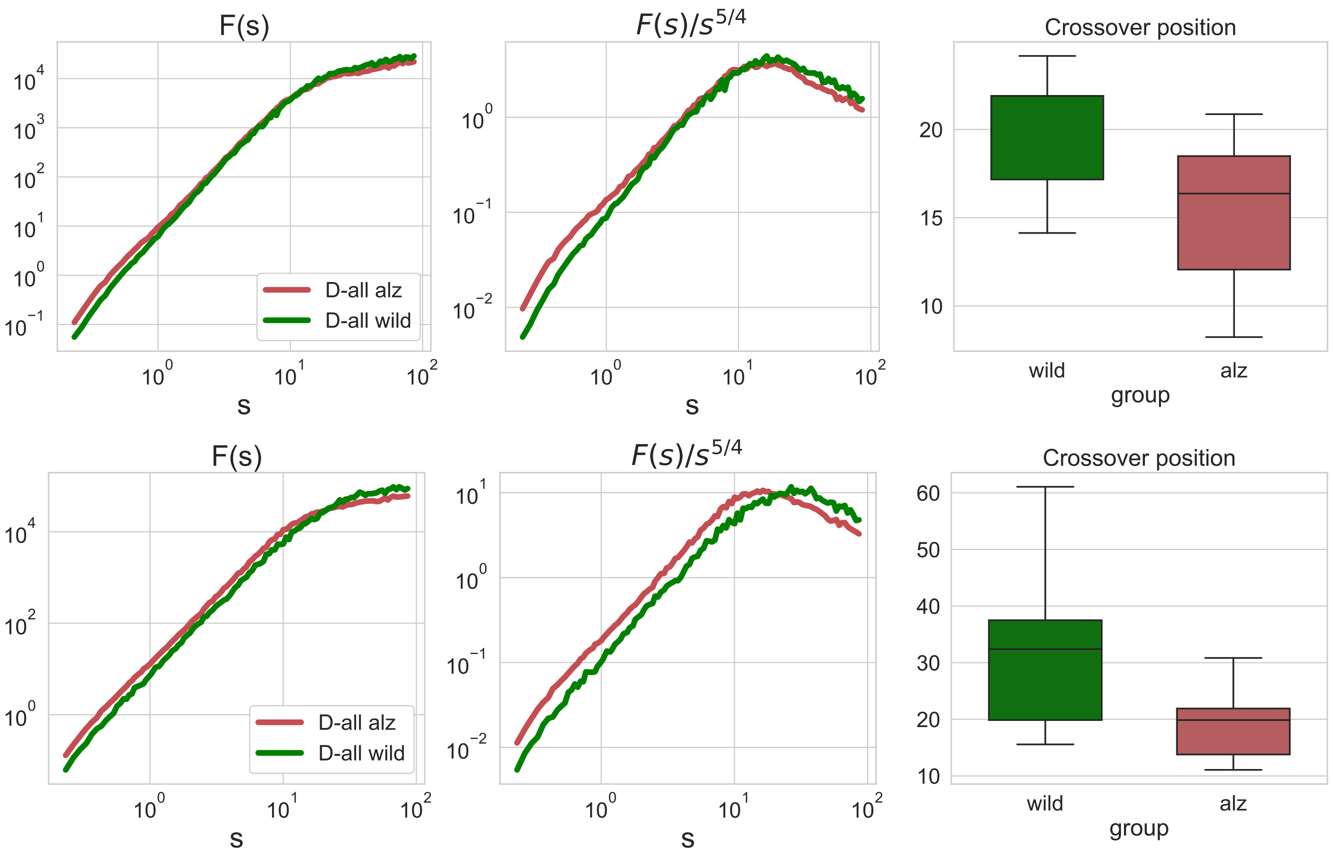


**Figure S11.** Similar fluctuation functions $F_{j_1,j_2}(s)$ as Fig. S4 for the relative movement trajectories between the animal body midpoint and other body parts $j_1 \neq j_2$ obtained by detrended cross-correlation analysis (DCCA).

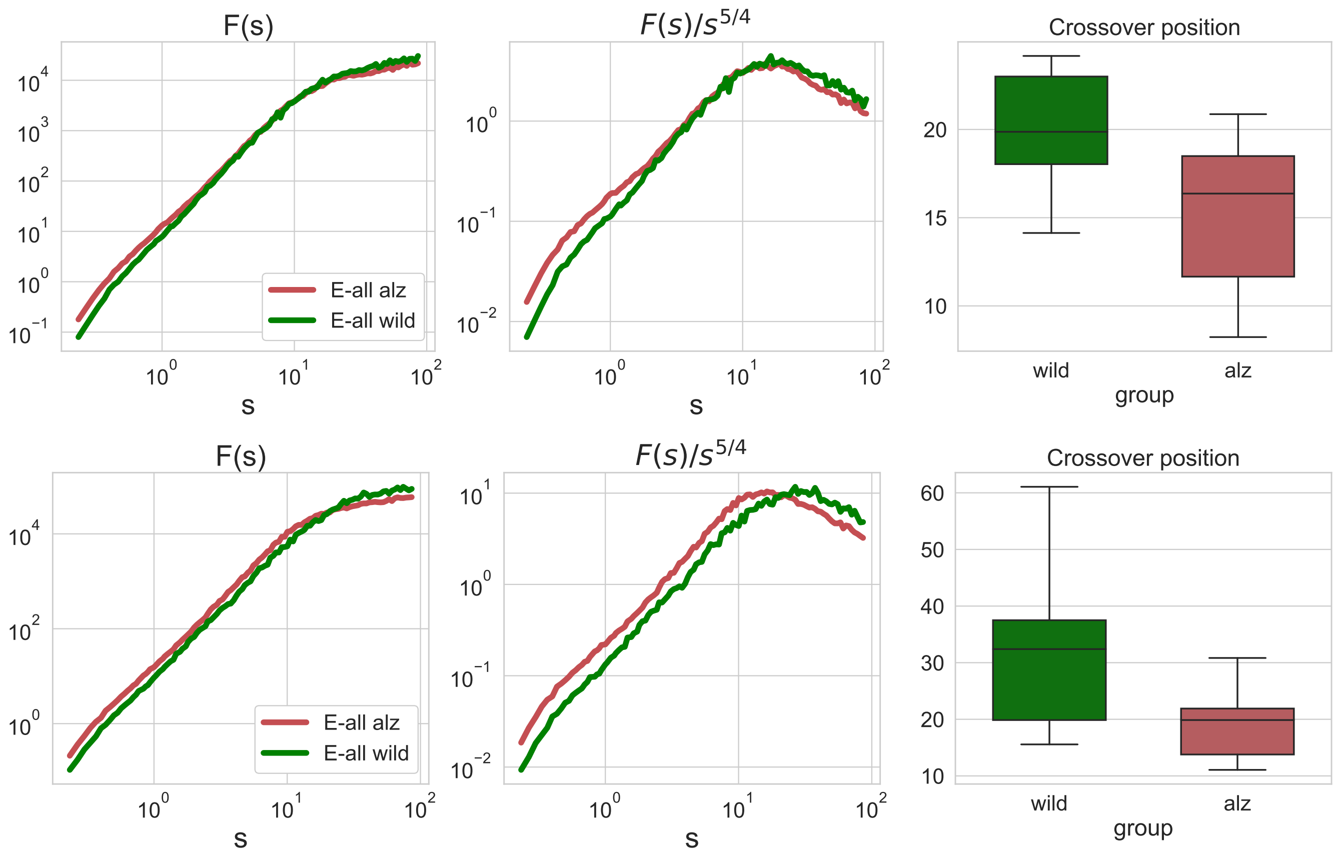


**Figure S12.** Similar fluctuation functions $F_{j_1,j_2}(s)$ as Fig. S5 for the relative movement trajectories between the left hind paw and other body parts $j_1 \neq j_2$ obtained by detrended cross-correlation analysis (DCCA).

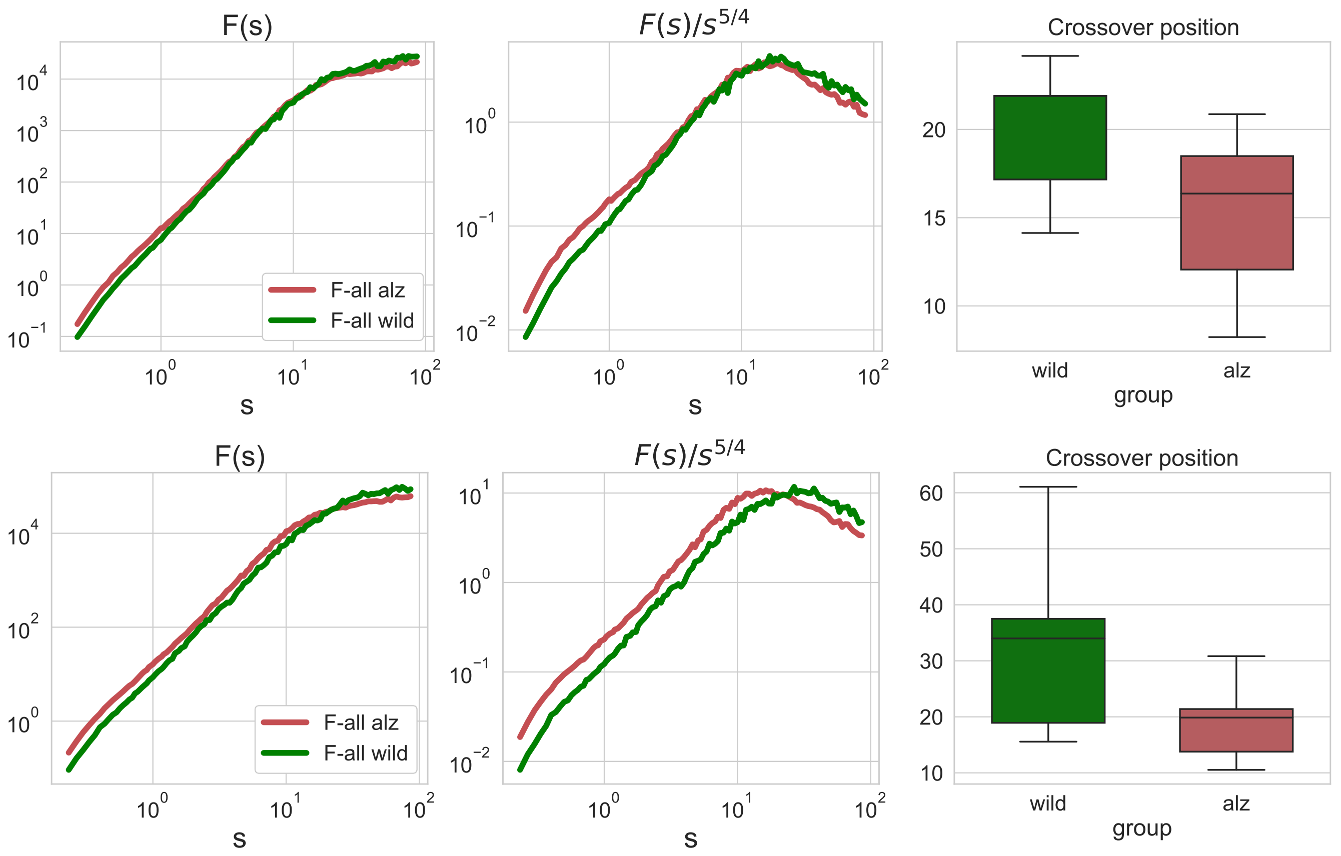


**Figure S13.** Similar fluctuation functions $F_{j_1,j_2}(s)$ as Fig. S6 for the relative movement trajectories between the right hind paw and other body parts $j_1 \neq j_2$ obtained by detrended cross-correlation analysis (DCCA).

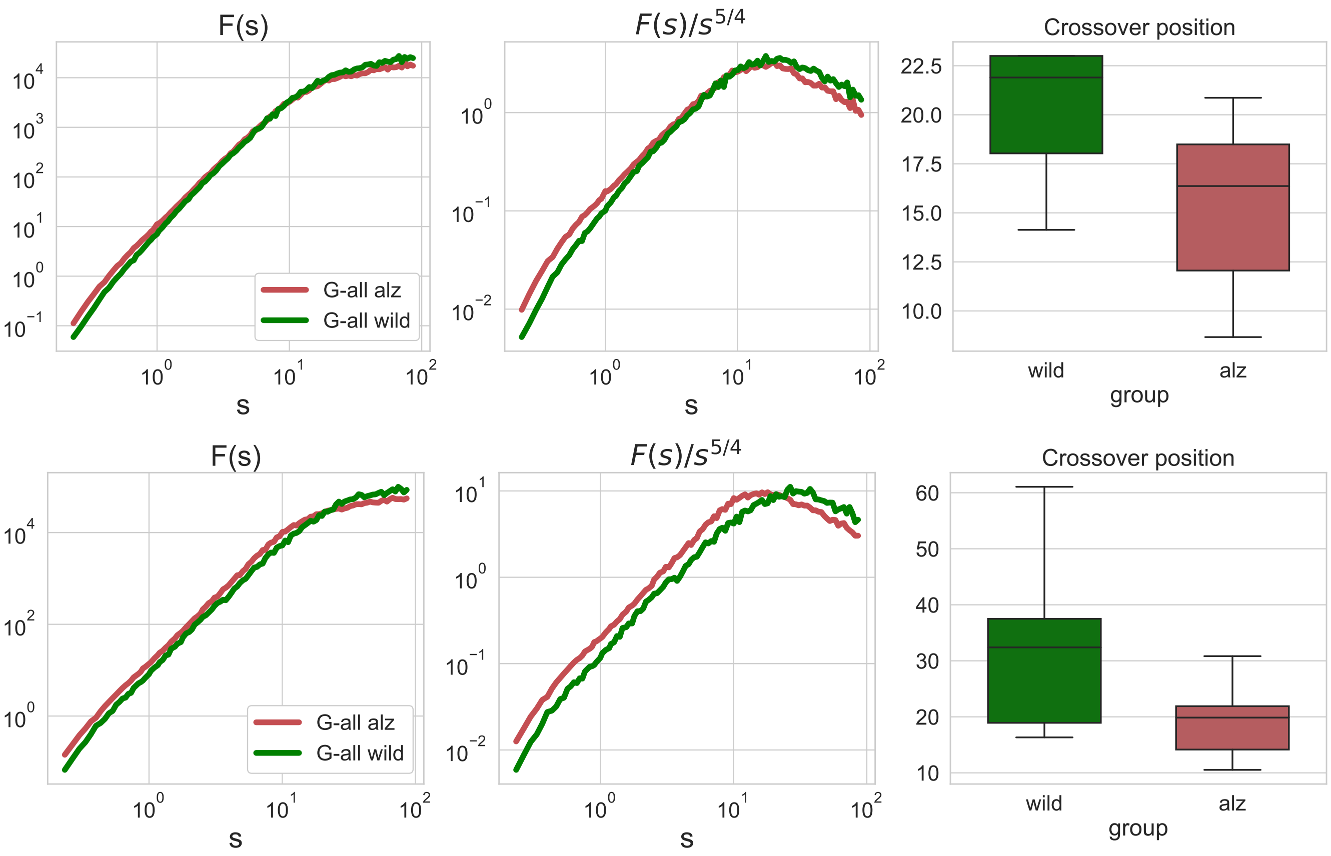


**Figure S14.** Similar fluctuation functions $F_{j_1,j_2}(s)$ as Fig. S1 for the relative movement trajectories between the tail and other body parts $j_1 \neq j_2$ obtained by detrended cross-correlation analysis (DCCA).

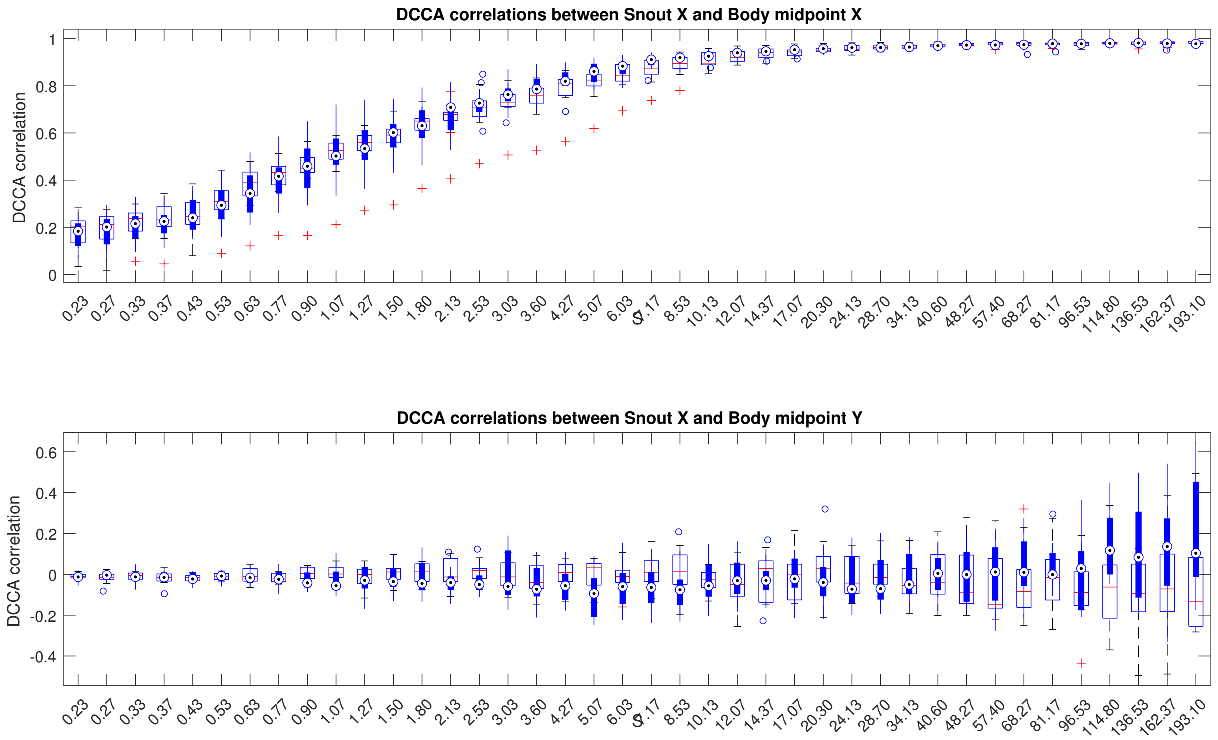


**Figure S15.** Boxplots indicating correlation dynamics of the snout along the X-axis *relative to the animal body midpoint* as a function of scale $S$. Movements along the same Cartesian coordinate axis denote specific correlations, while movements along the other axis indicate the level of unspecific correlations. Results for animals with Alzheimer's disease are provided by filled boxes and open circles for outliers; results for wild type animals (control group) are provided by open boxes with red plus signs for outliers.

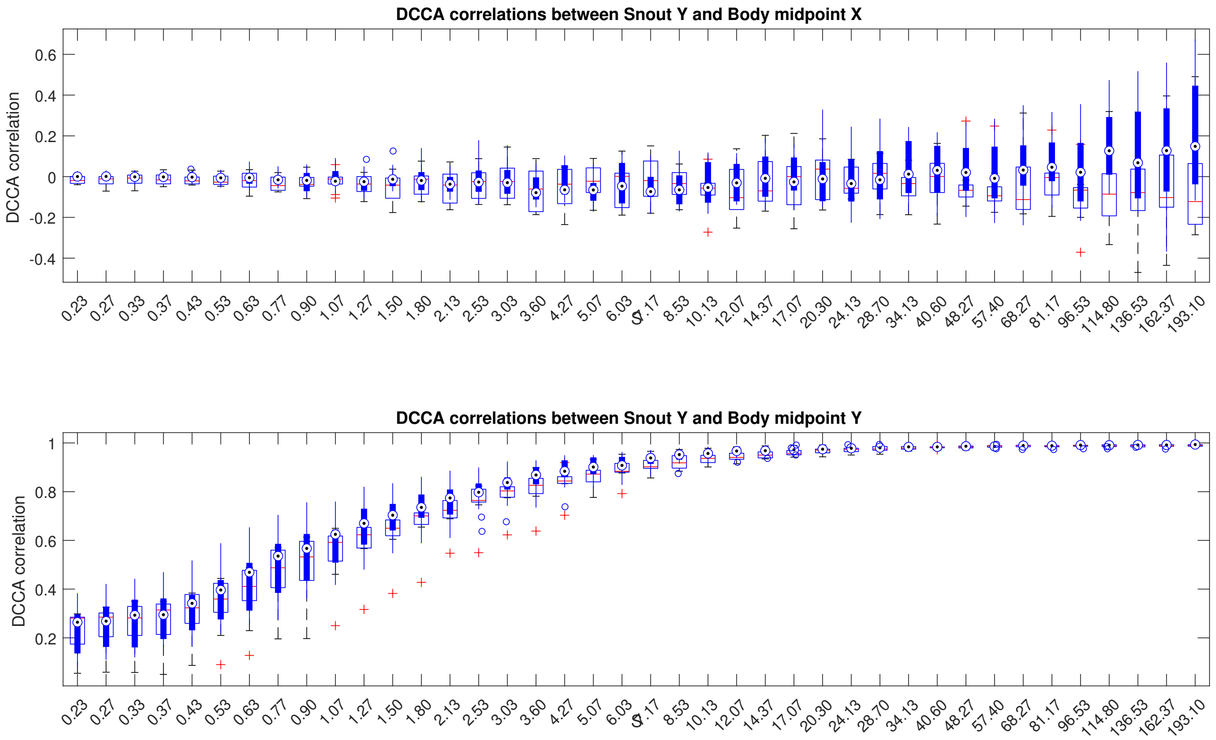


**Figure S16.** Boxplots indicating correlation dynamics of the snout along the Y-axis *relative to the animal body midpoint* as a function of scale $S$. Movements along the same Cartesian coordinate axis denote specific correlations, while movements along the other axis indicate the level of unspecific correlations. Results for animals with Alzheimer's disease are provided by filled boxes and open circles for outliers; results for wild type animals (control group) are provided by open boxes with red plus signs for outliers.

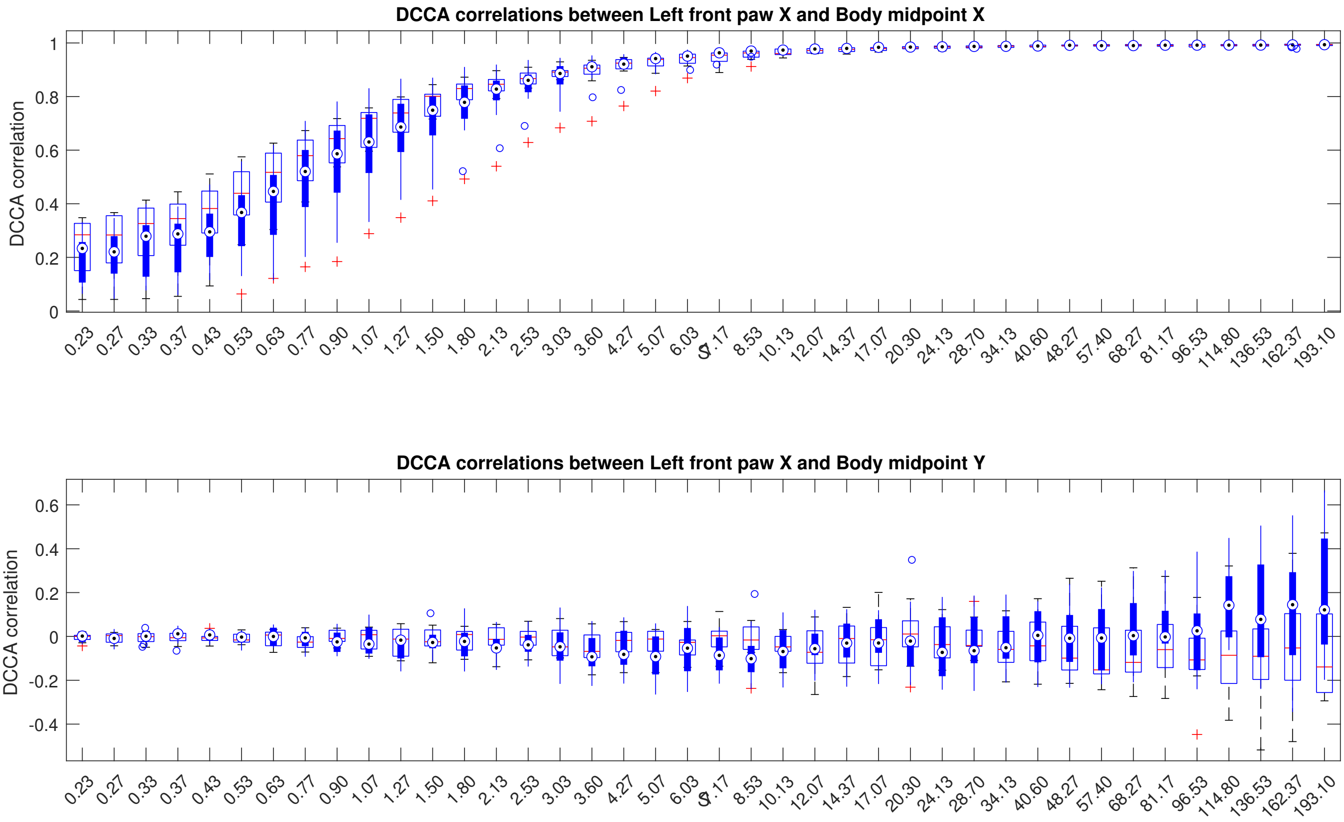


**Figure S17.** Boxplots indicating correlation dynamics of the left front paw along the X-axis *relative to the animal body midpoint* as a function of scale $S$. Movements along the same Cartesian coordinate axis denote specific correlations, while movements along the other axis indicate the level of unspecific correlations. Results for animals with Alzheimer's disease are provided by filled boxes and open circles for outliers; results for wild type animals (control group) are provided by open boxes with red plus signs for outliers.

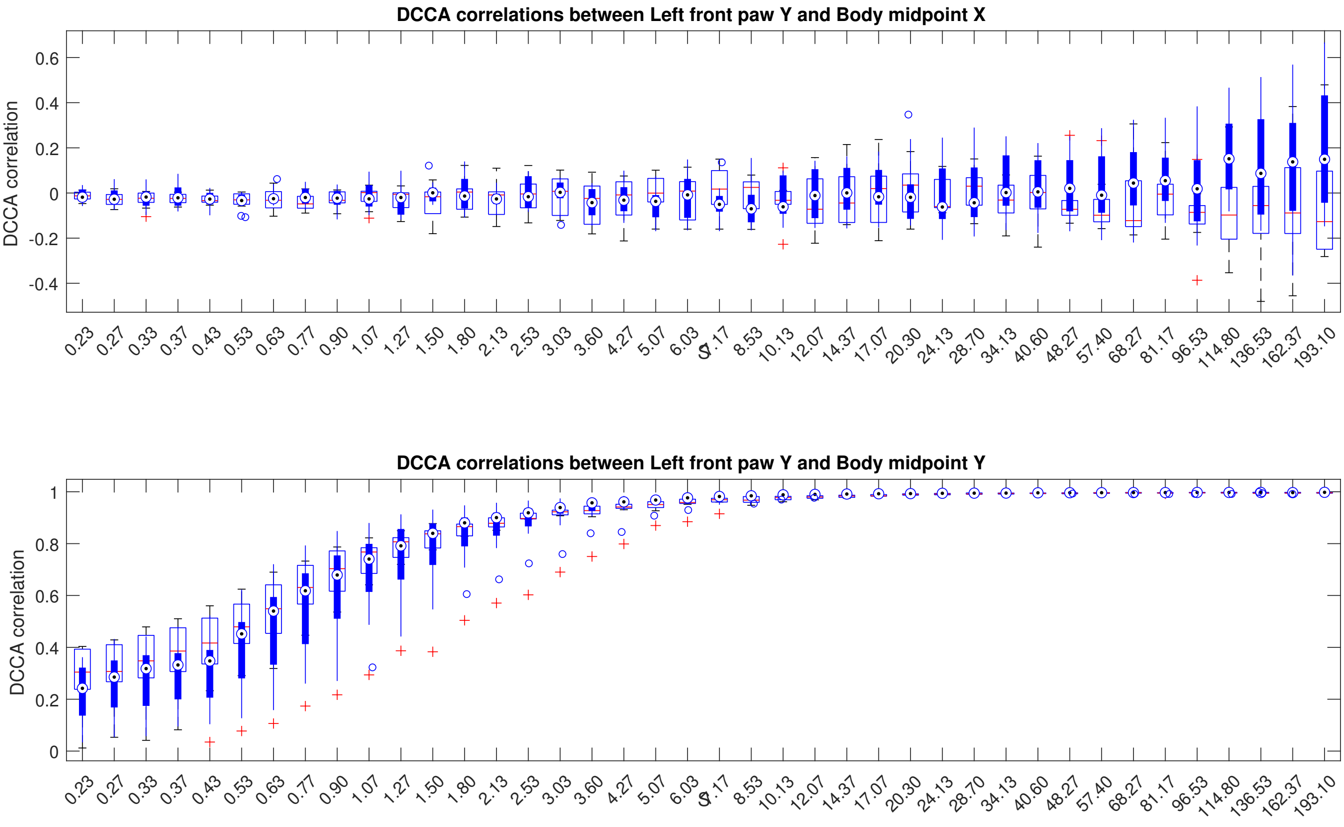


**Figure S18.** Boxplots indicating correlation dynamics of the left front paw along the Y-axis *relative to the animal body midpoint* as a function of scale $S$. Movements along the same Cartesian coordinate axis denote specific correlations, while movements along the other axis indicate the level of unspecific correlations. Results for animals with Alzheimer's disease are provided by filled boxes and open circles for outliers; results for wild type animals (control group) are provided by open boxes with red plus signs for outliers.

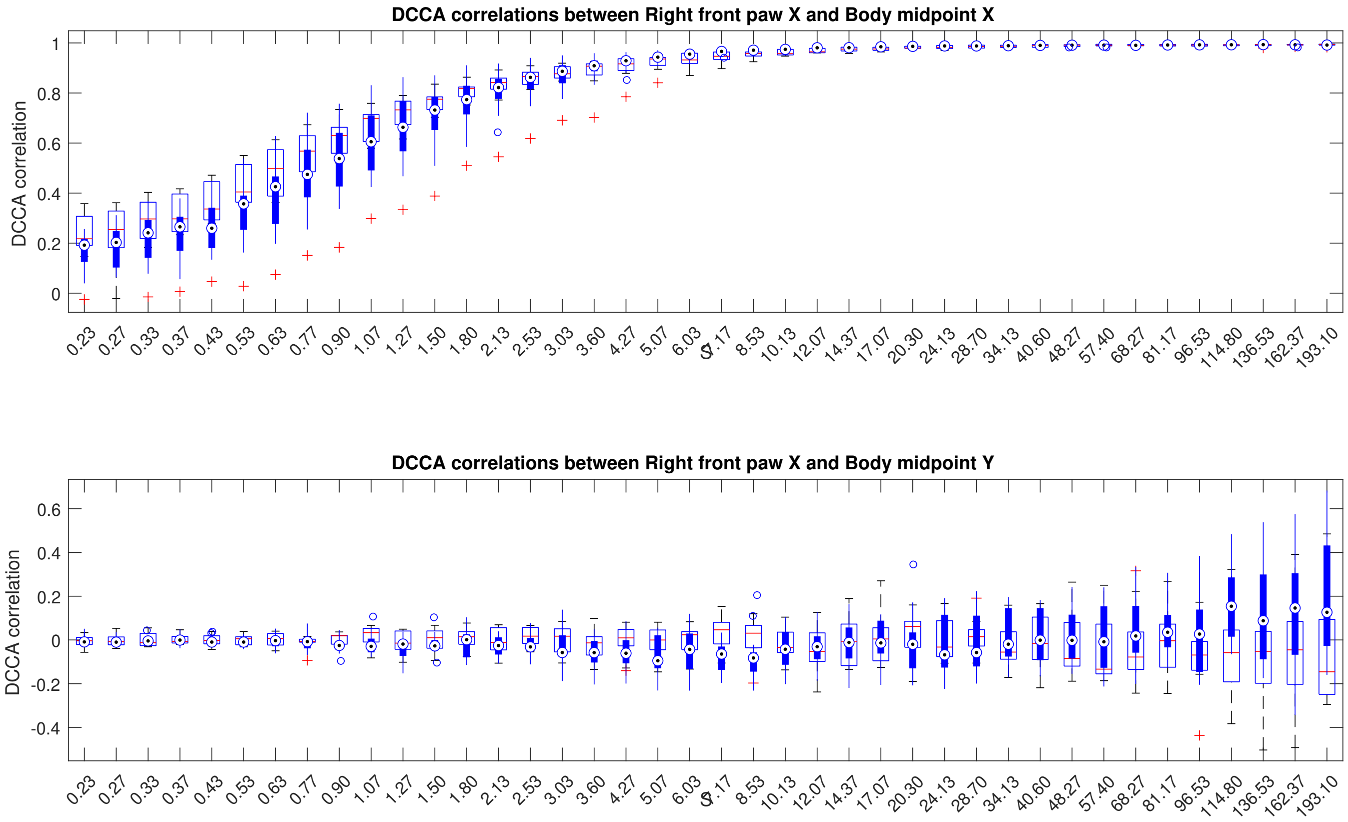


**Figure S19.** Boxplots indicating correlation dynamics of the right front paw along the X-axis *relative to the animal body midpoint* as a function of scale $S$. Movements along the same Cartesian coordinate axis denote specific correlations, while movements along the other axis indicate the level of unspecific correlations. Results for animals with Alzheimer's disease are provided by filled boxes and open circles for outliers; results for wild type animals (control group) are provided by open boxes with red plus signs for outliers.

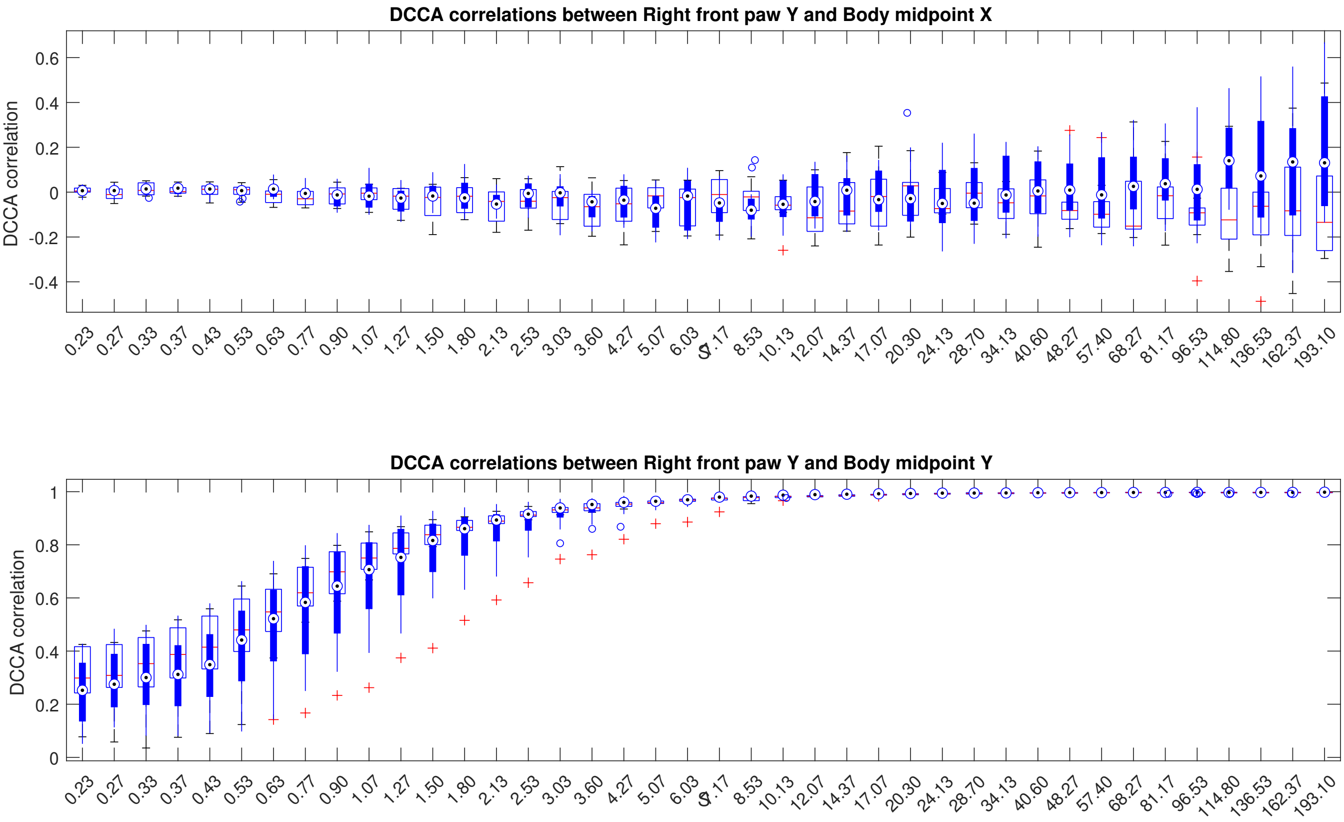


**Figure S20.** Boxplots indicating correlation dynamics of the right front paw along the Y-axis *relative to the animal body midpoint* as a function of scale $S$. Movements along the same Cartesian coordinate axis denote specific correlations, while movements along the other axis indicate the level of unspecific correlations. Results for animals with Alzheimer's disease are provided by filled boxes and open circles for outliers; results for wild type animals (control group) are provided by open boxes with red plus signs for outliers.

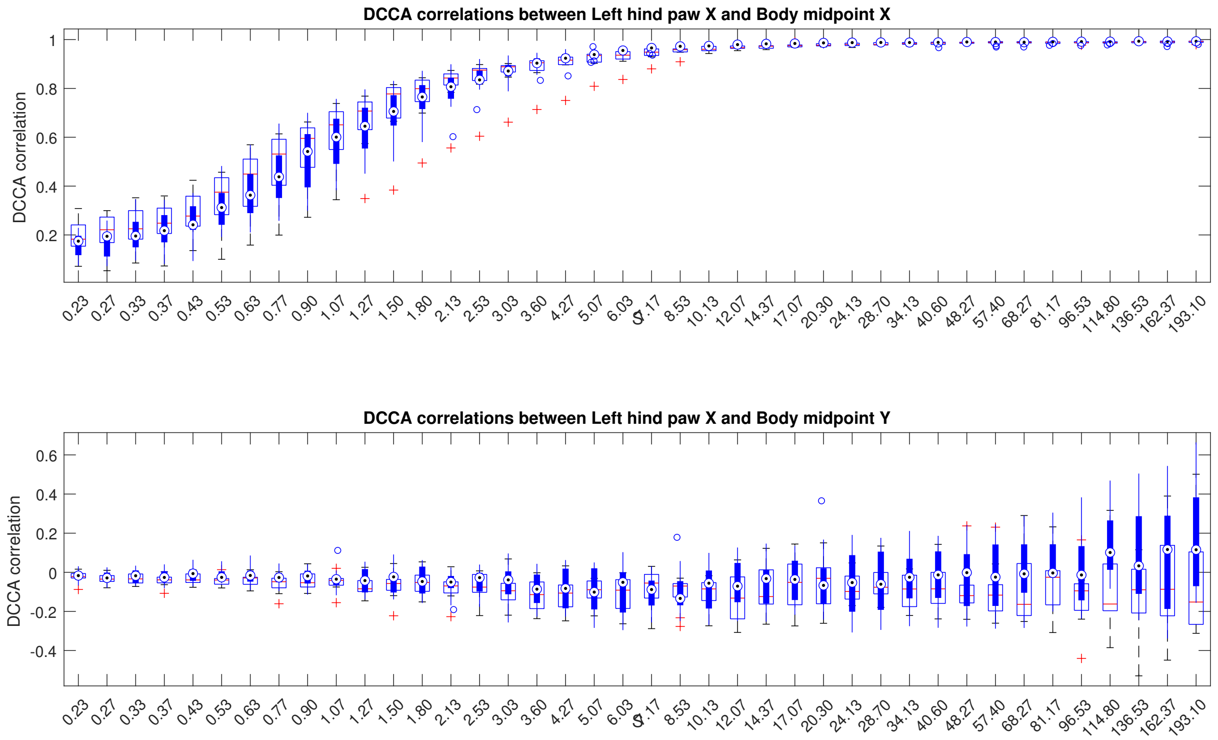


**Figure S21.** Boxplots indicating correlation dynamics of the left hind paw along the X-axis *relative to the animal body midpoint* as a function of scale $S$. Movements along the same Cartesian coordinate axis denote specific correlations, while movements along the other axis indicate the level of unspecific correlations. Results for animals with Alzheimer's disease are provided by filled boxes and open circles for outliers; results for wild type animals (control group) are provided by open boxes with red plus signs for outliers.

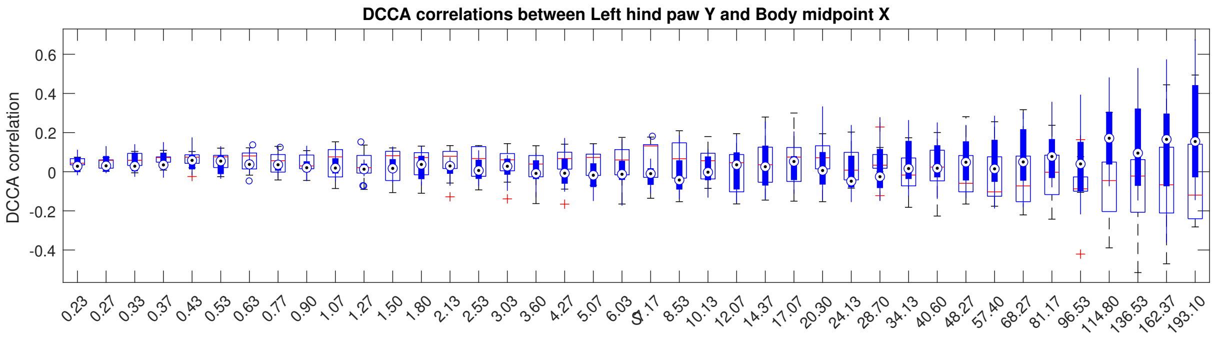


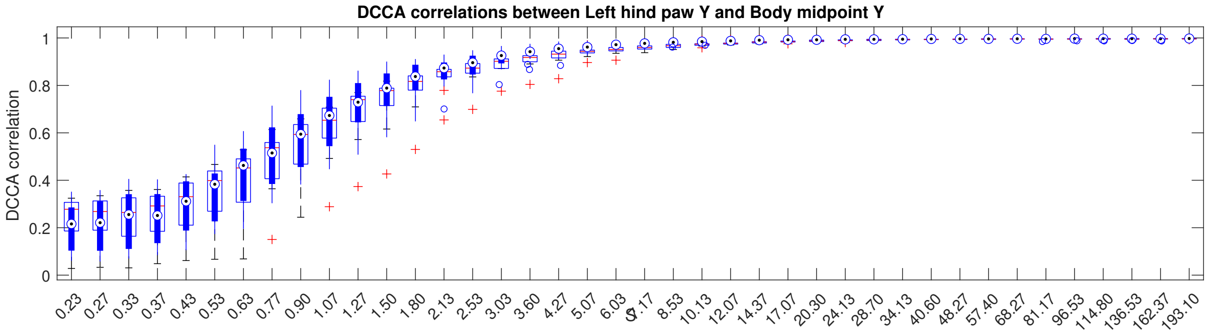


**Figure S22.** Boxplots indicating correlation dynamics of the left hind paw along the Y-axis *relative to the animal body midpoint* as a function of scale $S$. Movements along the same Cartesian coordinate axis denote specific correlations, while movements along the other axis indicate the level of unspecific correlations. Results for animals with Alzheimer's disease are provided by filled boxes and open circles for outliers; results for wild type animals (control group) are provided by open boxes with red plus signs for outliers.

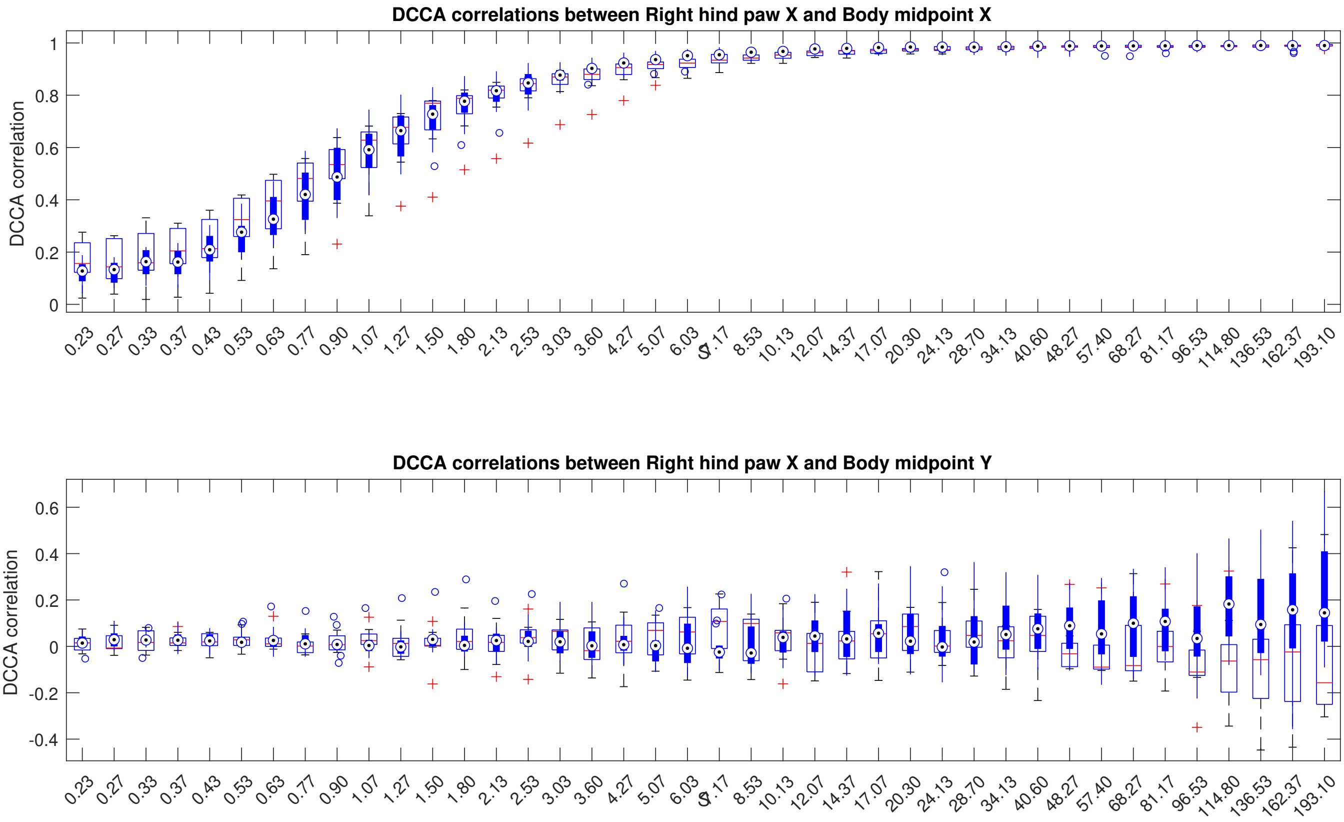


**Figure S23.** Boxplots indicating correlation dynamics of the right hind paw along the X-axis *relative to the animal body midpoint* as a function of scale $S$. Movements along the same Cartesian coordinate axis denote specific correlations, while movements along the other axis indicate the level of unspecific correlations. Results for animals with Alzheimer's disease are provided by filled boxes and open circles for outliers; results for wild type animals (control group) are provided by open boxes with red plus signs for outliers.

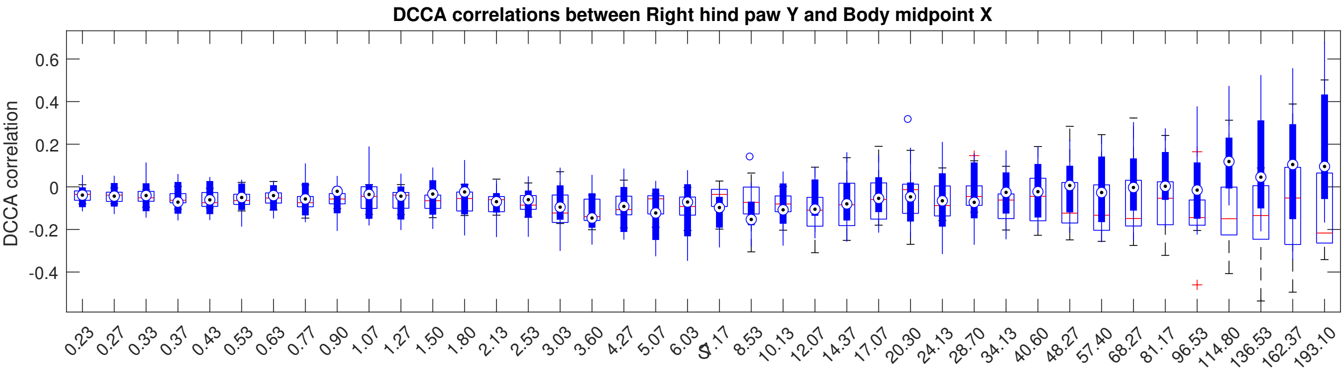


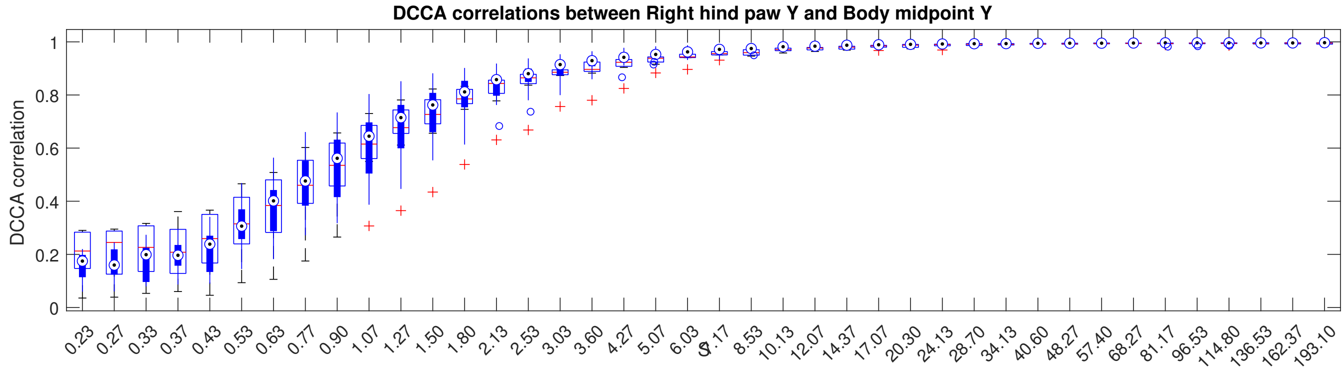


**Figure S24.** Boxplots indicating correlation dynamics of the right hind paw along the Y-axis *relative to the animal body midpoint* as a function of scale $S$. Movements along the same Cartesian coordinate axis denote specific correlations, while movements along the other axis indicate the level of unspecific correlations. Results for animals with Alzheimer's disease are provided by filled boxes and open circles for outliers; results for wild type animals (control group) are provided by open boxes with red plus signs for outliers.

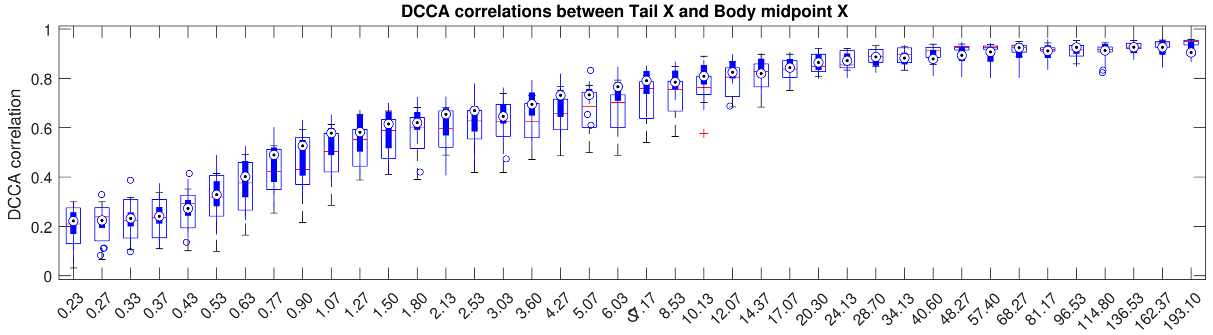


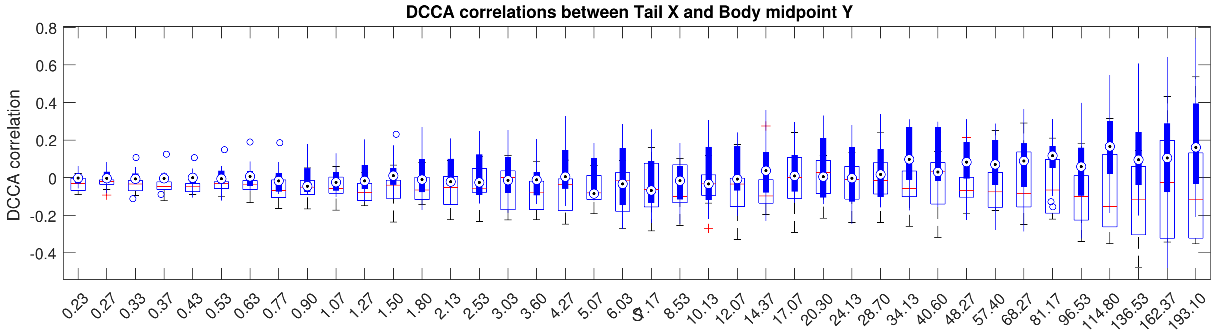


**Figure S25.** Boxplots indicating correlation dynamics of the tail along the X-axis *relative to the animal body midpoint* as a function of scale $S$. Movements along the same Cartesian coordinate axis denote specific correlations, while movements along the other axis indicate the level of unspecific correlations. Results for animals with Alzheimer's disease are provided by filled boxes and open circles for outliers; results for wild type animals (control group) are provided by open boxes with red plus signs for outliers.

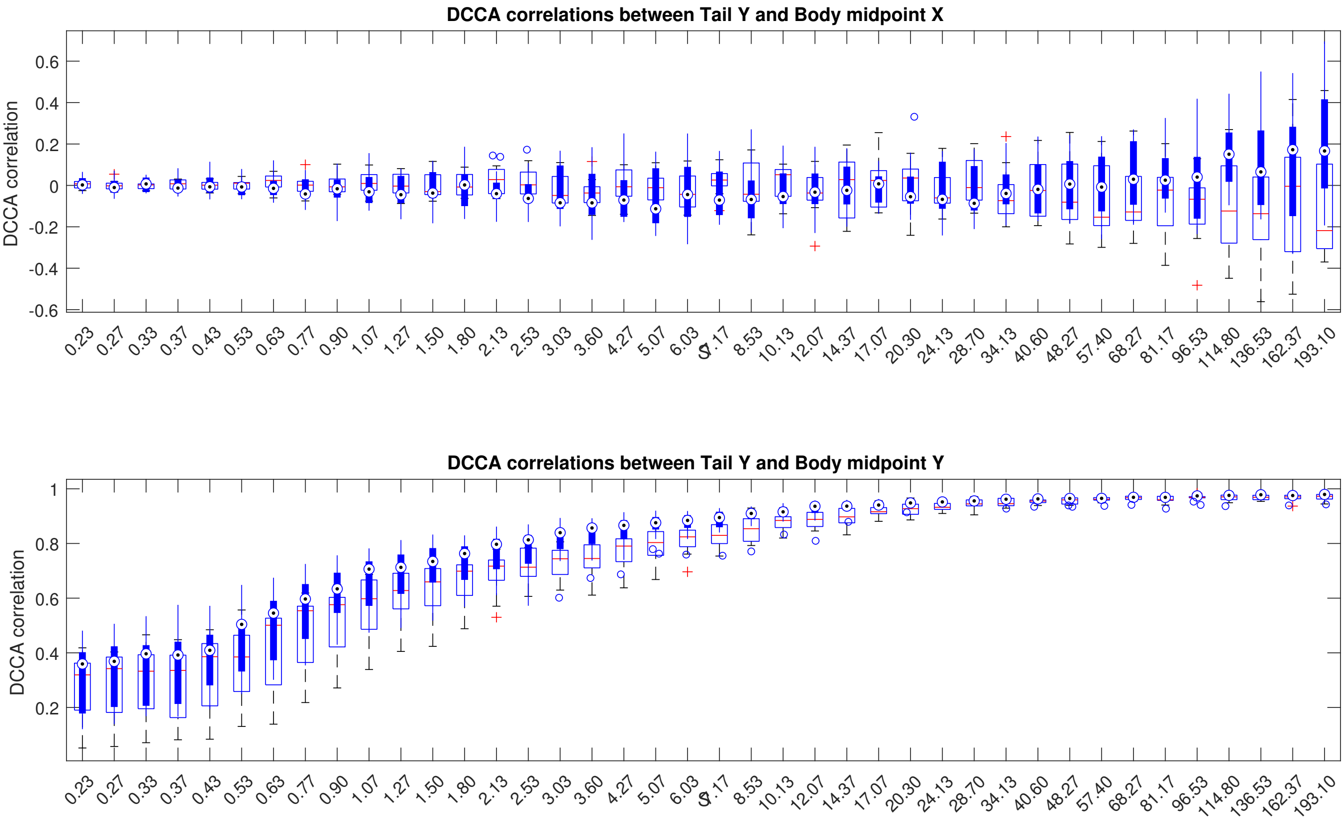


**Figure S26.** Boxplots indicating correlation dynamics of the tail along the Y-axis *relative to the animal body midpoint* as a function of scale $S$. Movements along the same Cartesian coordinate axis denote specific correlations, while movements along the other axis indicate the level of unspecific correlations. Results for animals with Alzheimer's disease are provided by filled boxes and open circles for outliers; results for wild type animals (control group) are provided by open boxes with red plus signs for outliers.

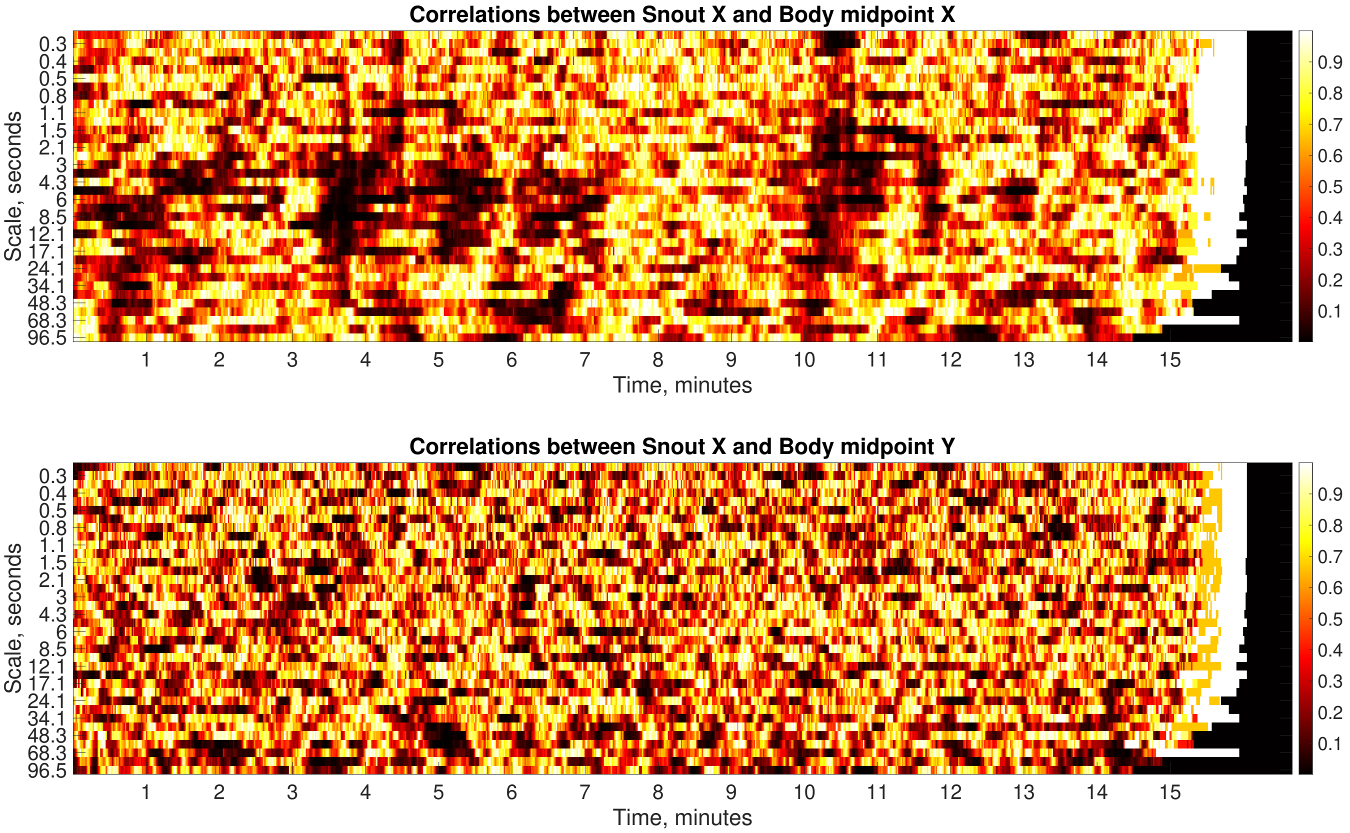


**Figure S27.** Time dependencies of p-values indicating statistical significance of discrepancies between correlation dynamics of the snout along the X-axis *relative to the animal body midpoint* as a function of scale $S$. Movements along the same Cartesian coordinate axis denote specific correlations, while movements along the other axis indicate the level of unspecific correlations. Results for animals with Alzheimer's disease are provided by filled boxes and open circles for outliers; results for wild type animals (control group) are provided by open boxes with red plus signs for outliers.

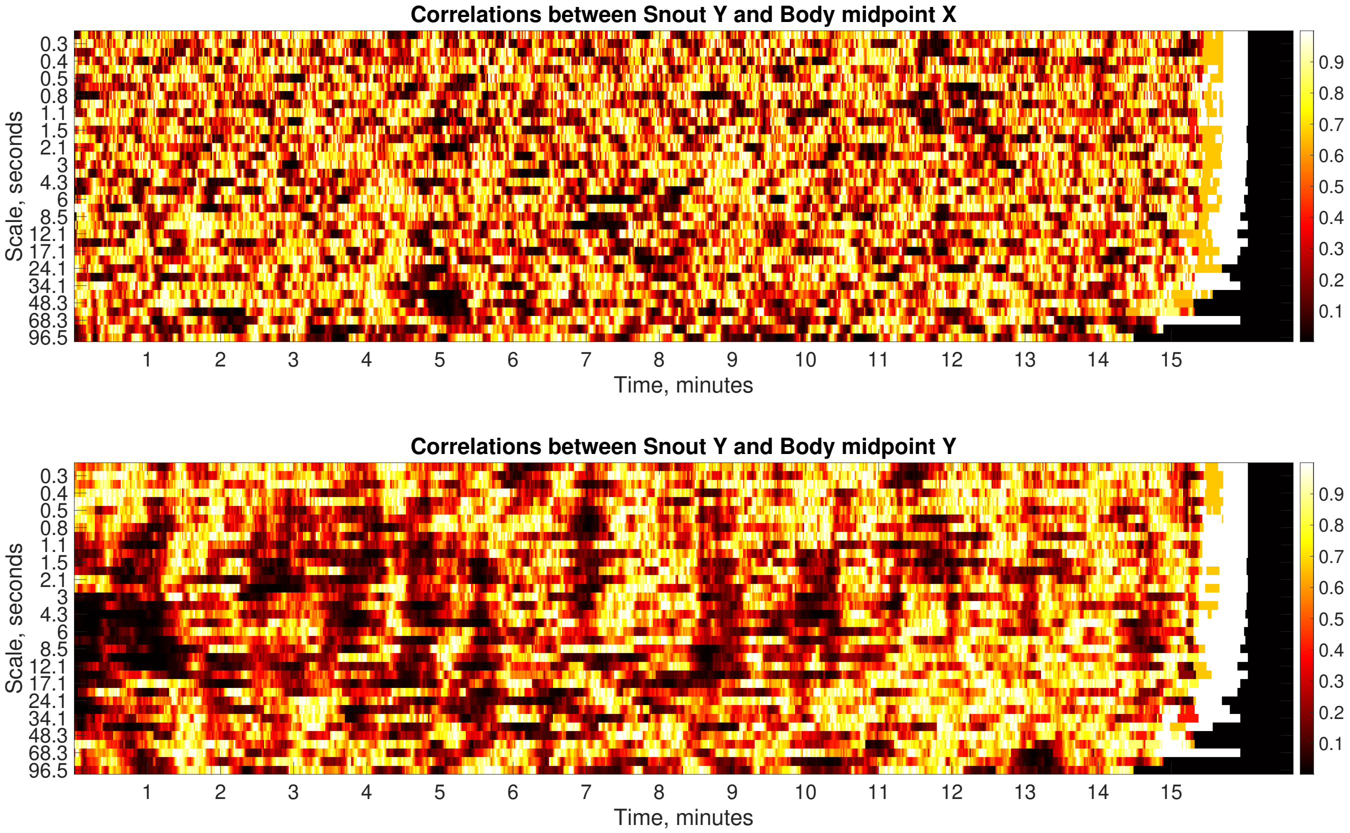


**Figure S28.** Time dependencies of p-values indicating statistical significance of discrepancies between correlation dynamics of the snout along the Y-axis *relative to the animal body midpoint* as a function of scale $S$. Movements along the same Cartesian coordinate axis denote specific correlations, while movements along the other axis indicate the level of unspecific correlations.

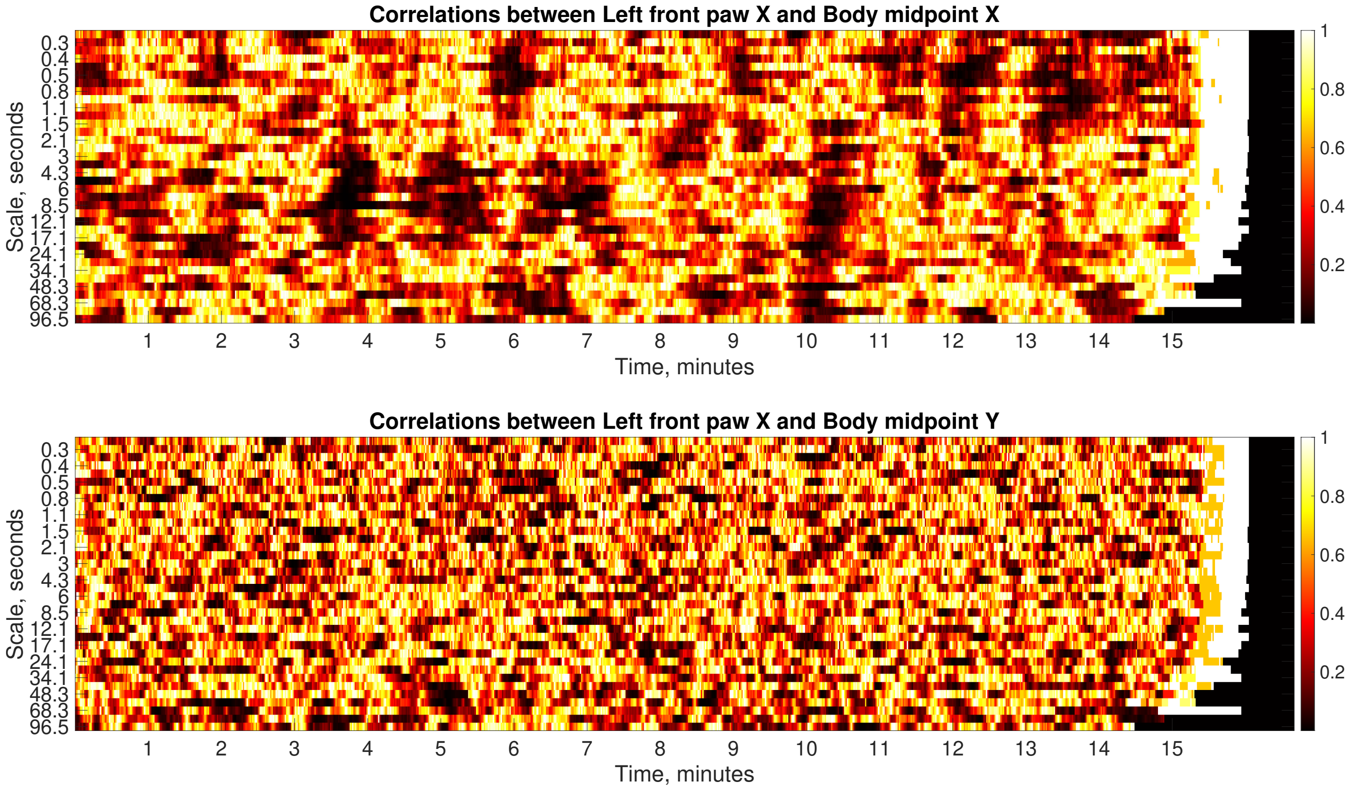


**Figure S29.** Time dependencies of p-values indicating statistical significance of discrepancies between correlation dynamics of the left front paw along the X-axis *relative to the animal body midpoint* as a function of scale $S$. Movements along the same Cartesian coordinate axis denote specific correlations, while movements along the other axis indicate the level of unspecific correlations.

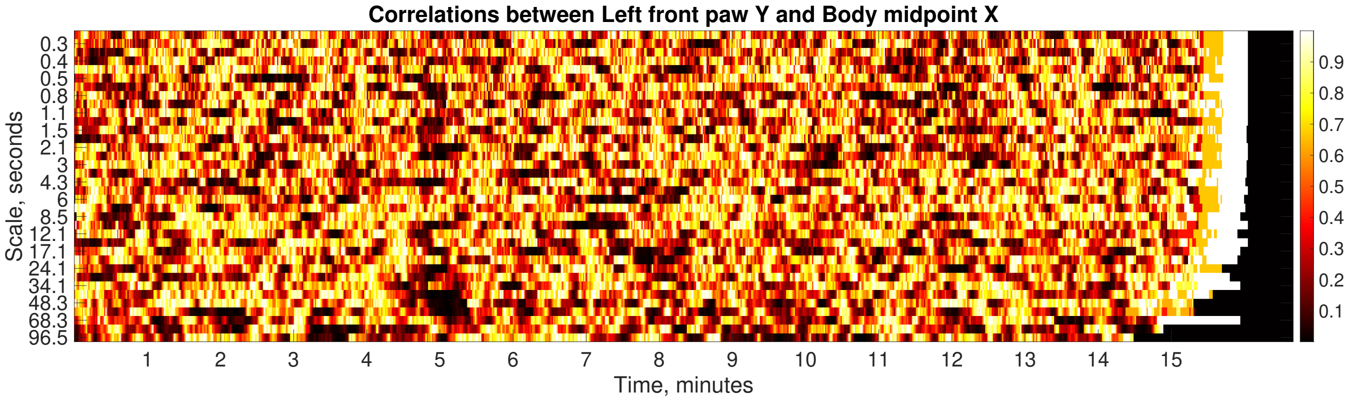


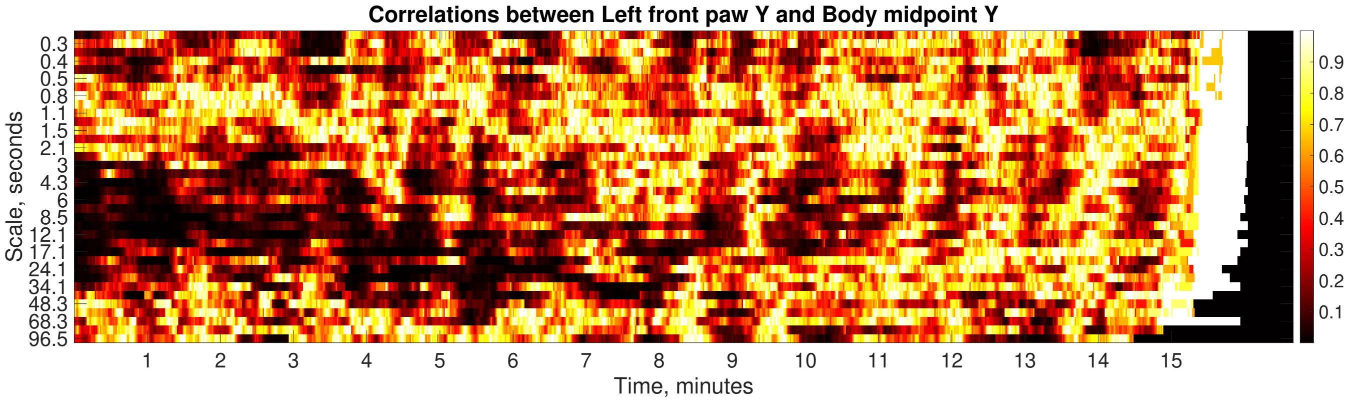


**Figure S30.** Time dependencies of p-values indicating statistical significance of discrepancies between correlation dynamics of the left front paw along the Y-axis *relative to the animal body midpoint* as a function of scale $S$. Movements along the same Cartesian coordinate axis denote specific correlations, while movements along the other axis indicate the level of unspecific correlations.

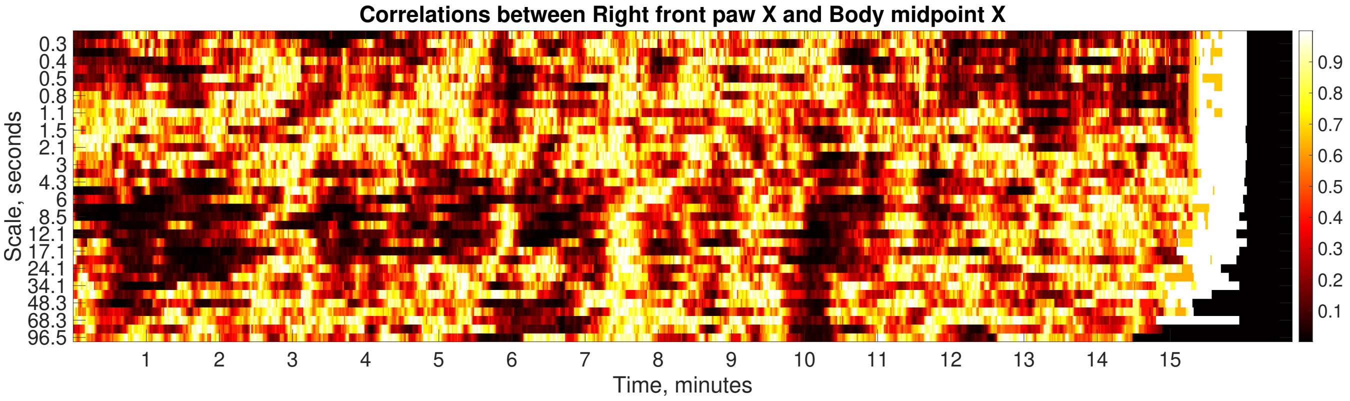


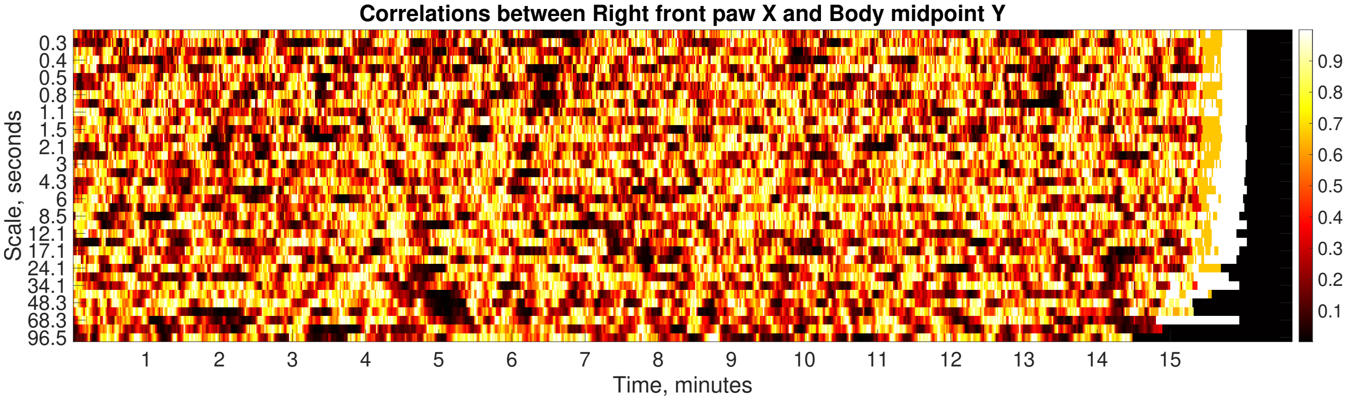


**Figure S31.** Time dependencies of p-values indicating statistical significance of discrepancies between correlation dynamics of the right front paw along the X-axis *relative to the animal body midpoint* as a function of scale $S$. Movements along the same Cartesian coordinate axis denote specific correlations, while movements along the other axis indicate the level of unspecific correlations.

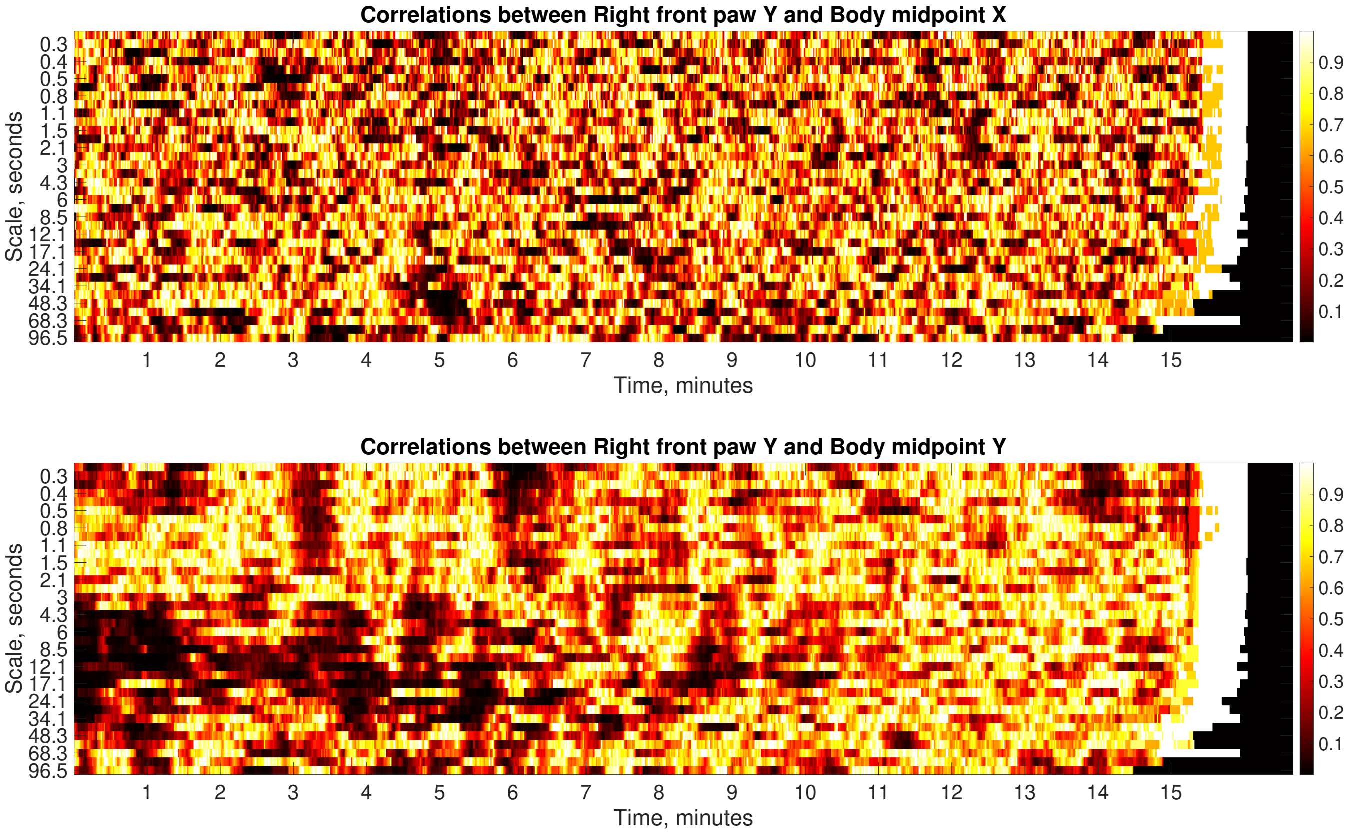


**Figure S32.** Time dependencies of p-values indicating statistical significance of discrepancies between correlation dynamics of the right front paw along the Y-axis *relative to the animal body midpoint* as a function of scale $S$. Movements along the same Cartesian coordinate axis denote specific correlations, while movements along the other axis indicate the level of unspecific correlations.

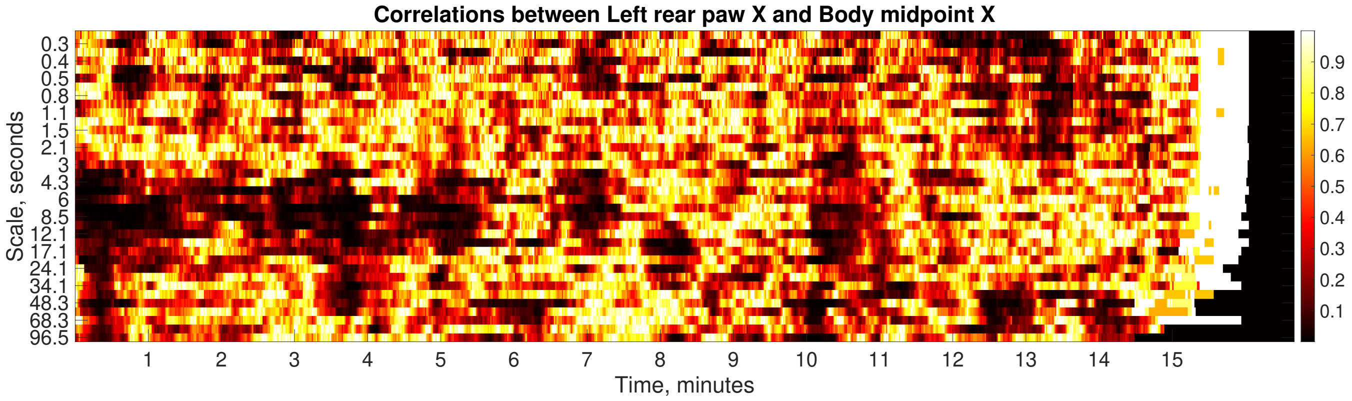


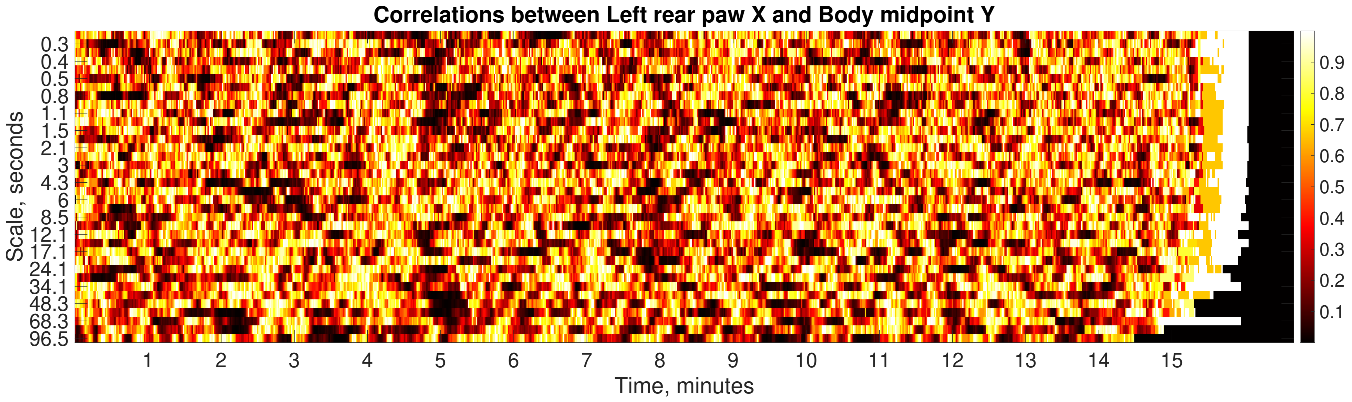


**Figure S33.** Time dependencies of p-values indicating statistical significance of discrepancies between correlation dynamics of the left hind paw along the X-axis *relative to the animal body midpoint* as a function of scale $S$. Movements along the same Cartesian coordinate axis denote specific correlations, while movements along the other axis indicate the level of unspecific correlations.

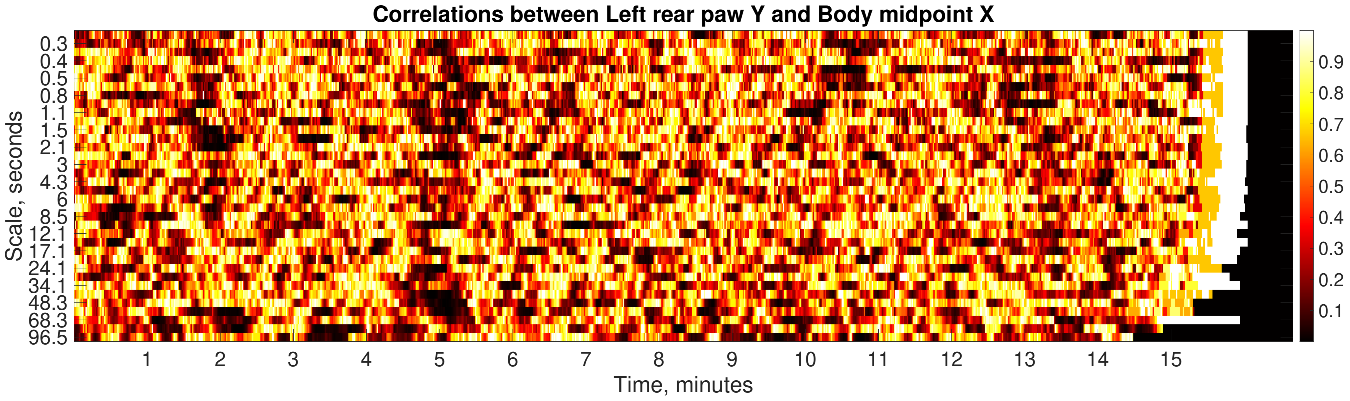


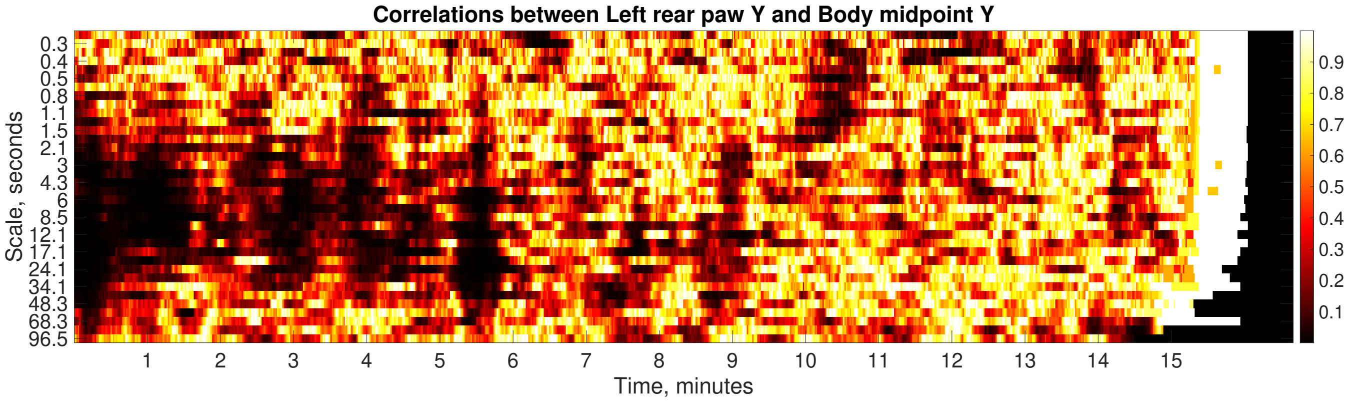


**Figure S34.** Time dependencies of p-values indicating statistical significance of discrepancies between correlation dynamics of the left hind paw along the Y-axis *relative to the animal body midpoint* as a function of scale $S$. Movements along the same Cartesian coordinate axis denote specific correlations, while movements along the other axis indicate the level of unspecific correlations.

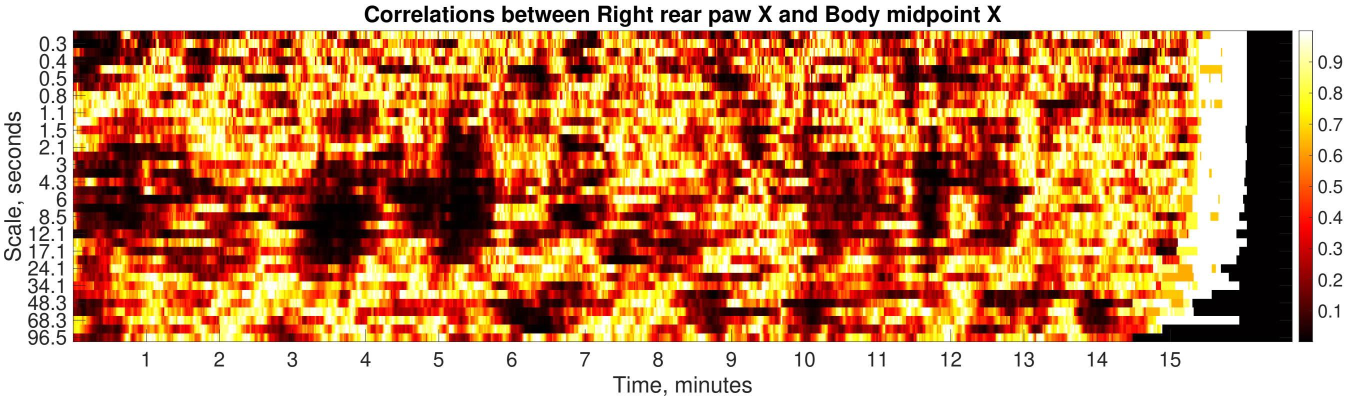


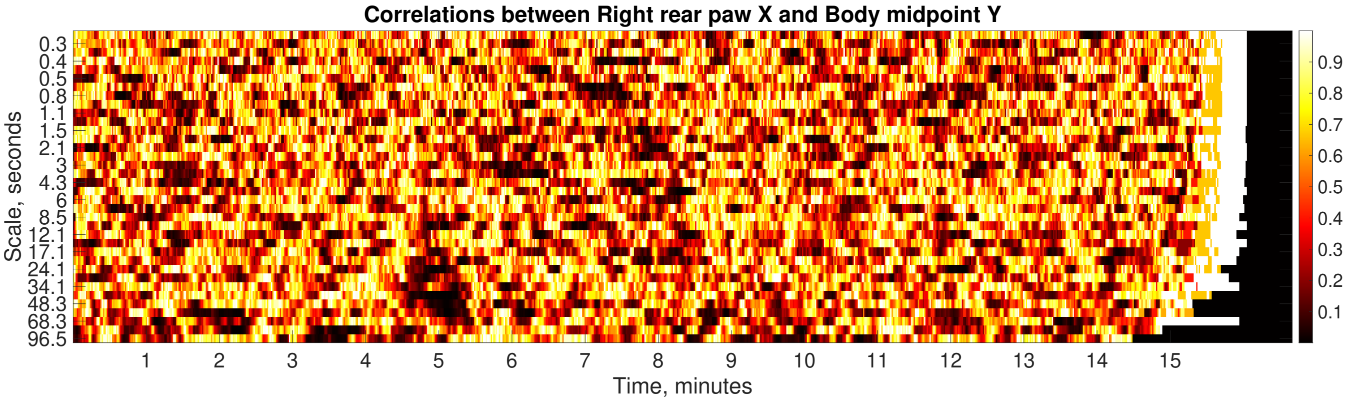


**Figure S35.** Time dependencies of p-values indicating statistical significance of discrepancies between correlation dynamics of the right hind paw along the X-axis *relative to the animal body midpoint* as a function of scale $S$. Movements along the same Cartesian coordinate axis denote specific correlations, while movements along the other axis indicate the level of unspecific correlations.

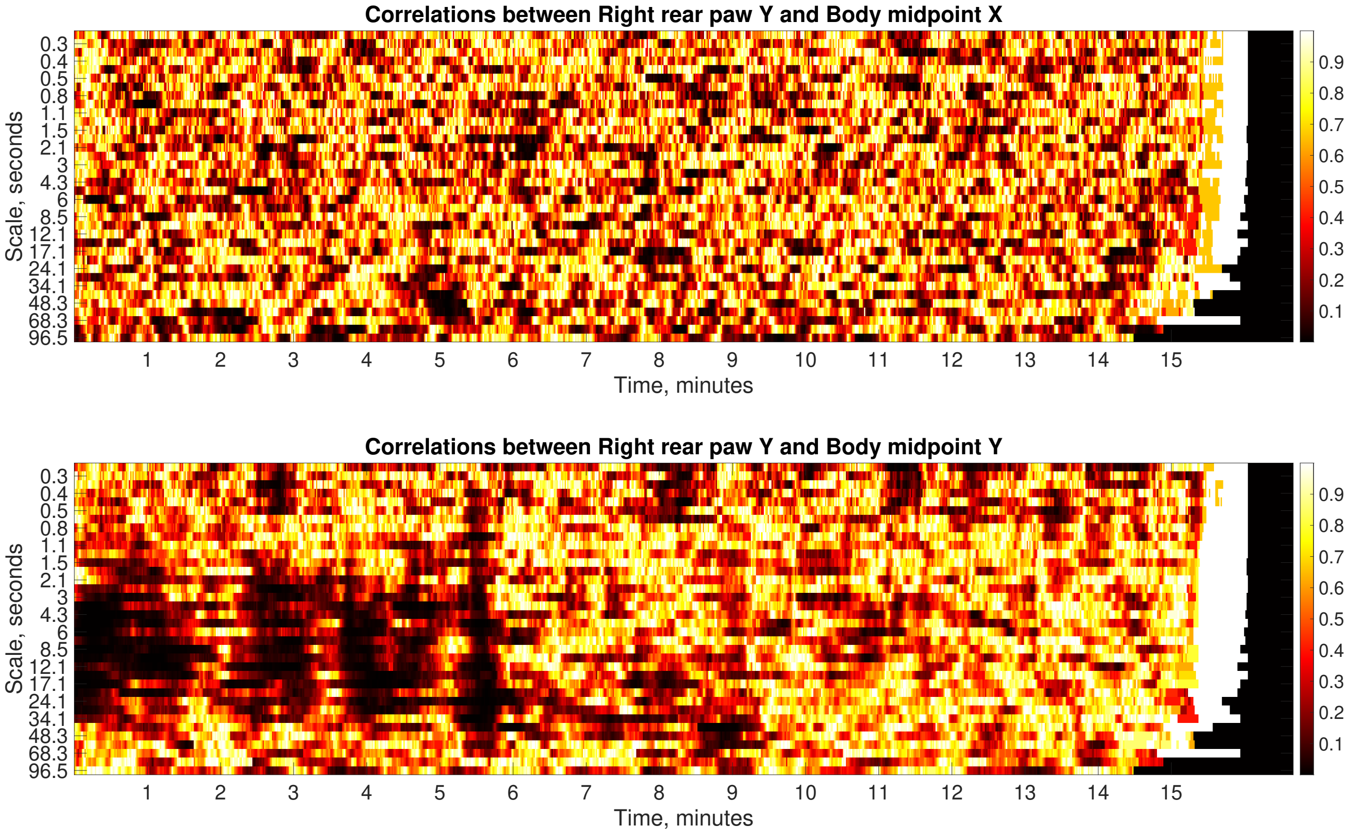


**Figure S36.** Time dependencies of p-values indicating statistical significance of discrepancies between correlation dynamics of the right hind paw along the Y-axis *relative to the animal body midpoint* as a function of scale $S$. Movements along the same Cartesian coordinate axis denote specific correlations, while movements along the other axis indicate the level of unspecific correlations.

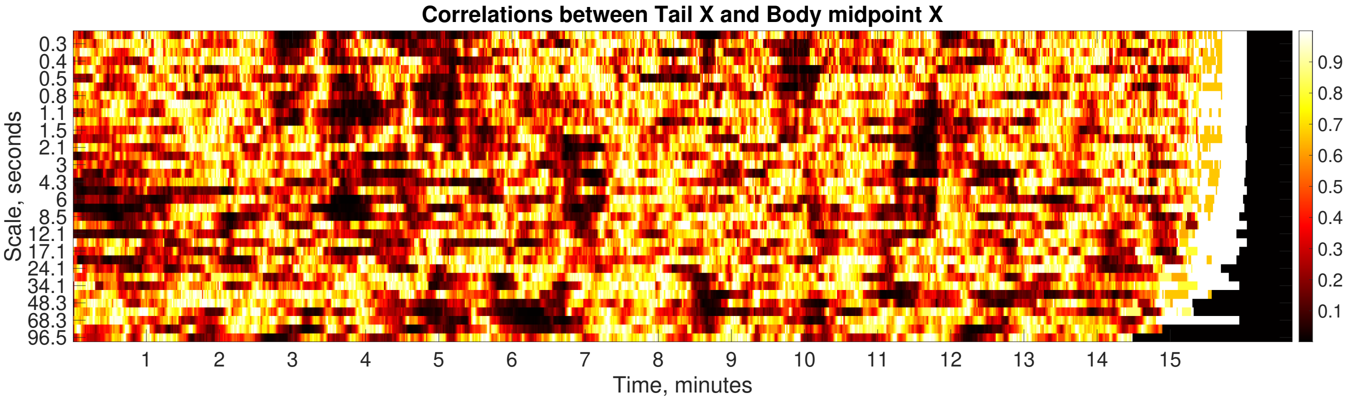


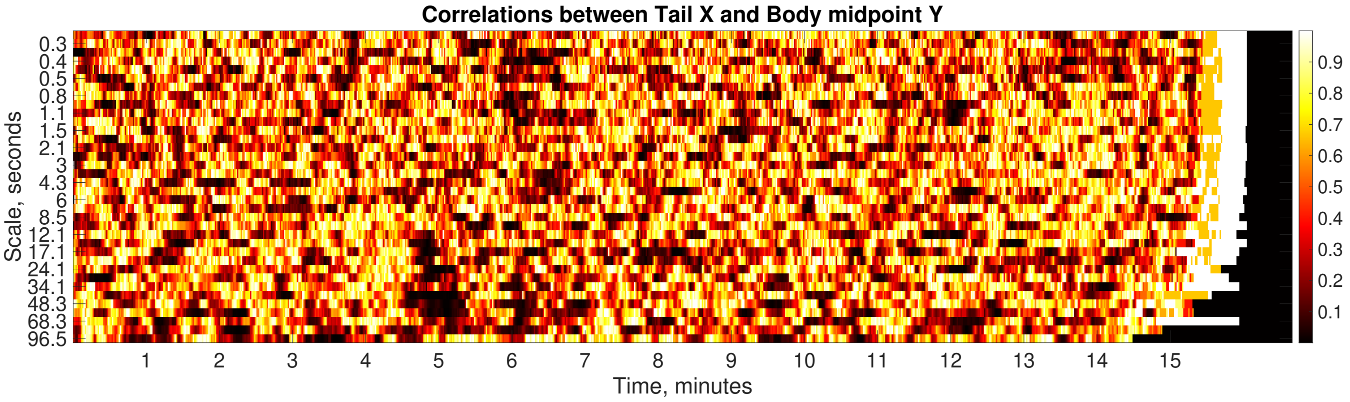


**Figure S37.** Time dependencies of p-values indicating statistical significance of discrepancies between correlation dynamics of the tail along the X-axis *relative to the animal body midpoint* as a function of scale $S$. Movements along the same Cartesian coordinate axis denote specific correlations, while movements along the other axis indicate the level of unspecific correlations.

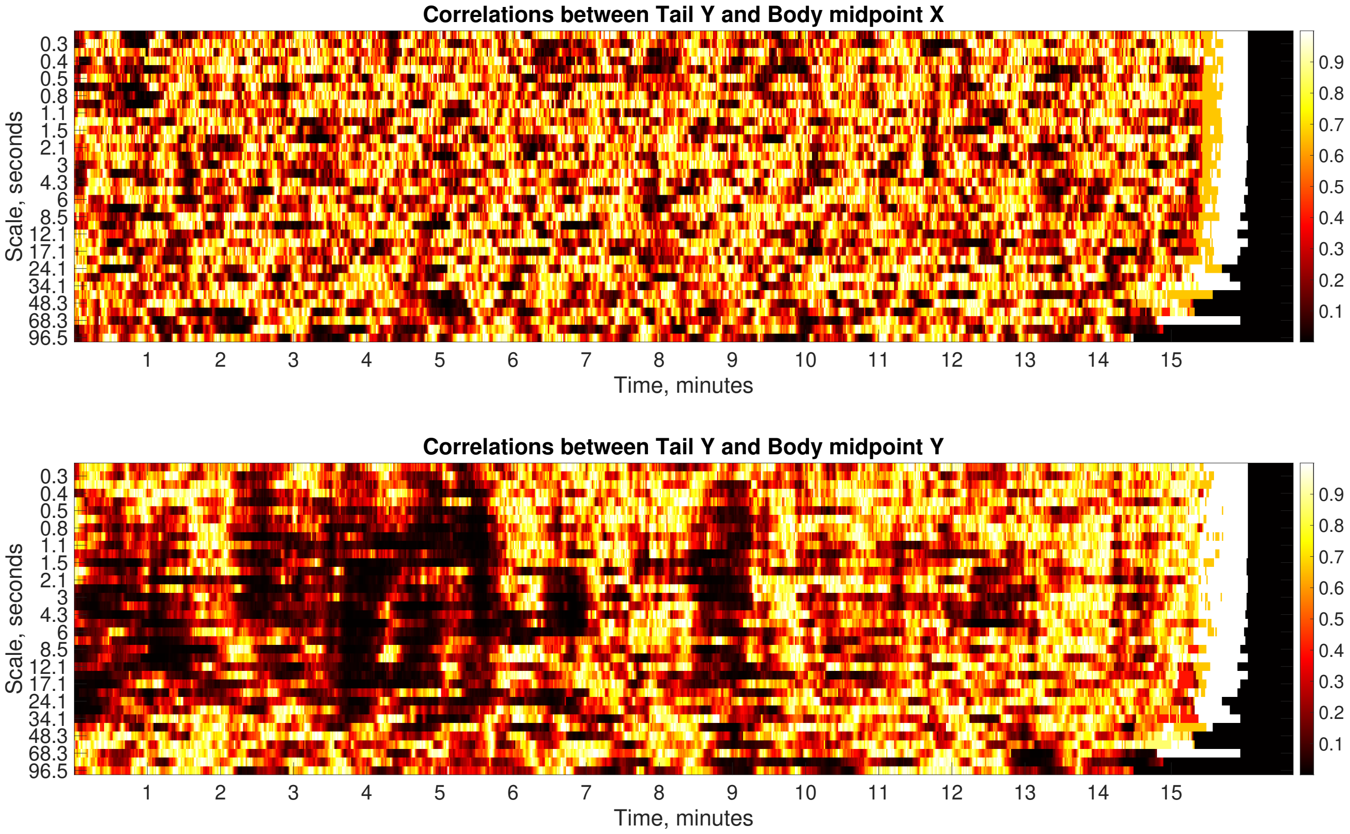


**Figure S38.** Time dependencies of p-values indicating statistical significance of discrepancies between correlation dynamics of the tail along the Y-axis *relative to the animal body midpoint* as a function of scale $S$. Movements along the same Cartesian coordinate axis denote specific correlations, while movements along the other axis indicate the level of unspecific correlations.

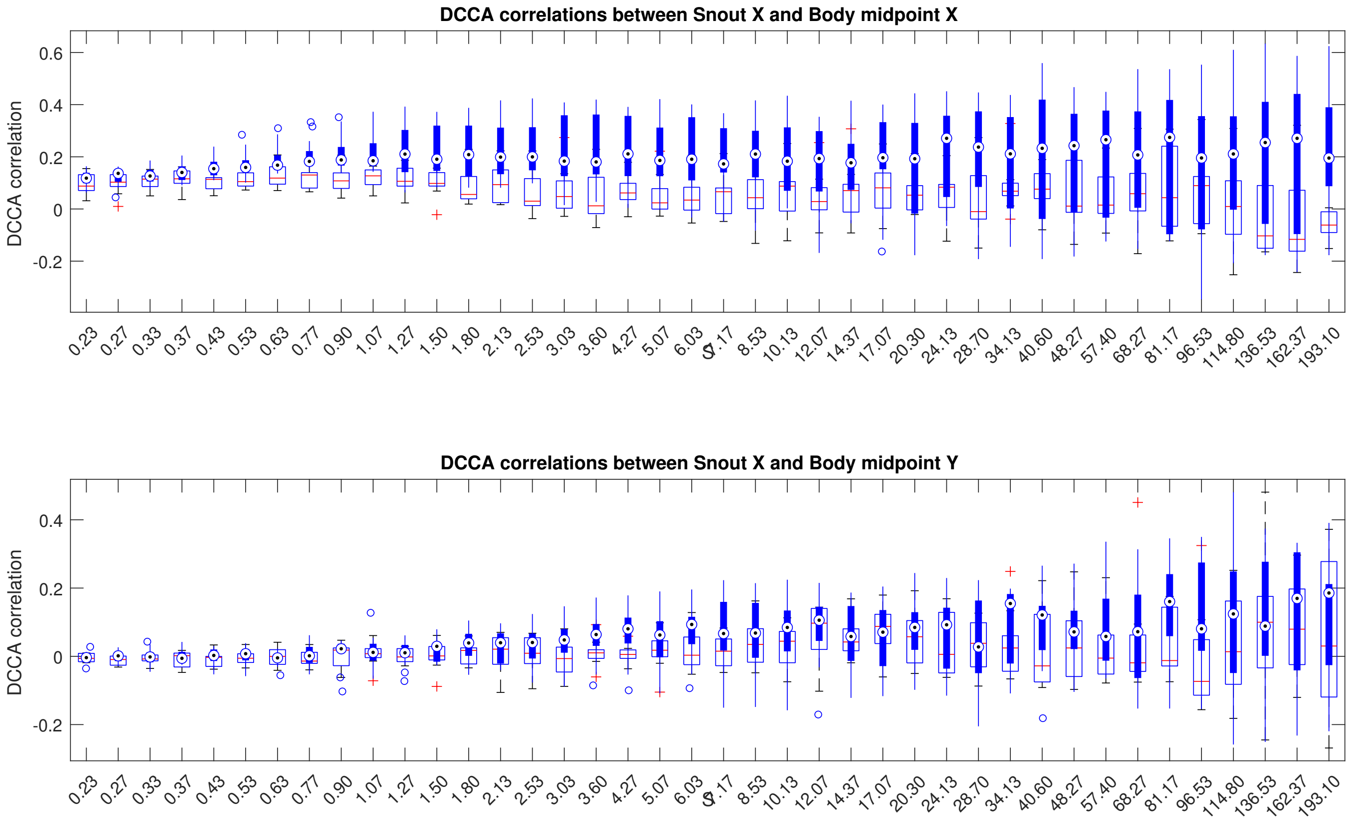


**Figure S39.** Boxplots indicating correlation dynamics of the snout along the X-axis *relative to the animal body midpoint* as a function of scale $S$. Movements along the same Cartesian coordinate axis denote specific correlations, while movements along the other axis indicate the level of unspecific correlations. Results for animals with Alzheimer's disease are provided by filled boxes and open circles for outliers; results for wild type animals (control group) are provided by open boxes with red plus signs for outliers.

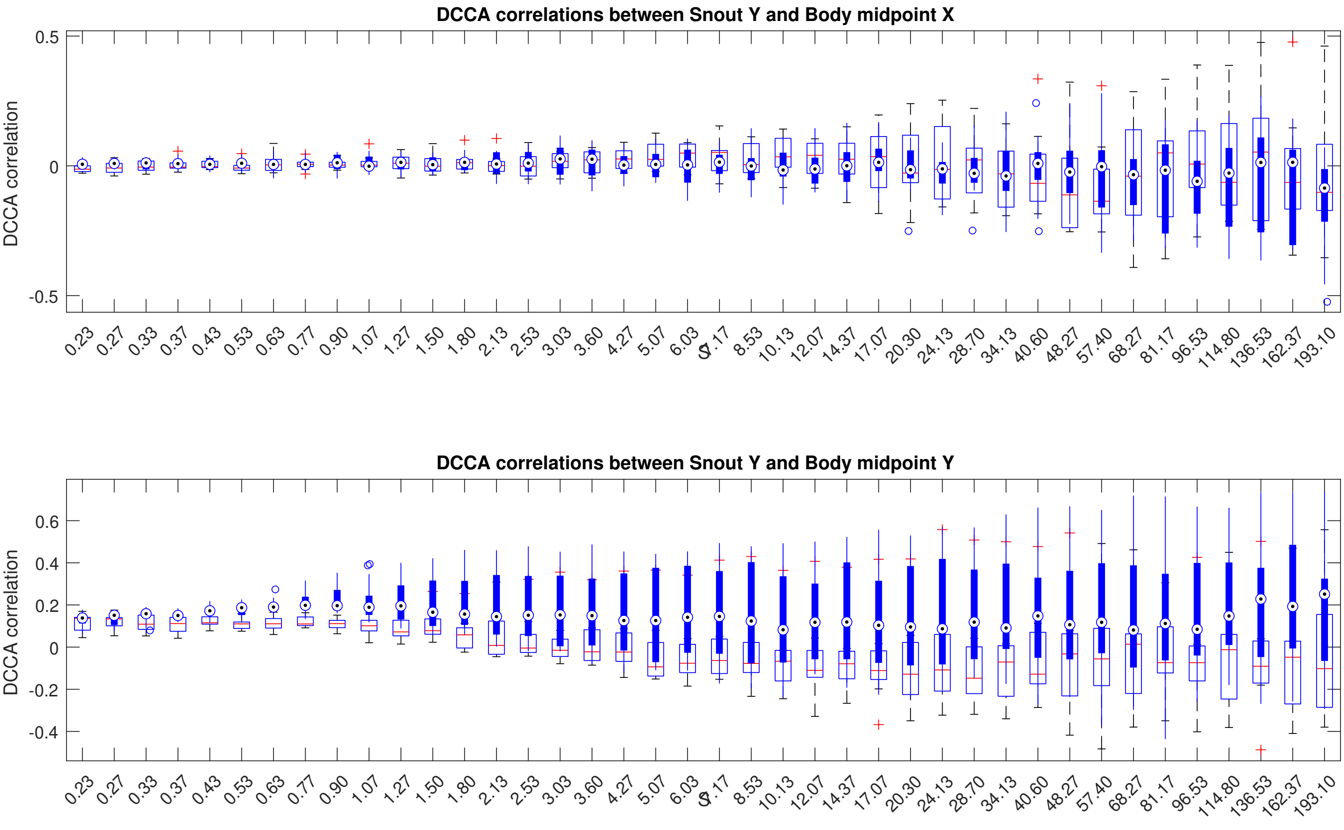


**Figure S40.** Boxplots indicating correlation dynamics of the snout along the Y-axis *relative to the animal body midpoint* as a function of scale $S$. Movements along the same Cartesian coordinate axis denote specific correlations, while movements along the other axis indicate the level of unspecific correlations. Results for animals with Alzheimer's disease are provided by filled boxes and open circles for outliers; results for wild type animals (control group) are provided by open boxes with red plus signs for outliers.

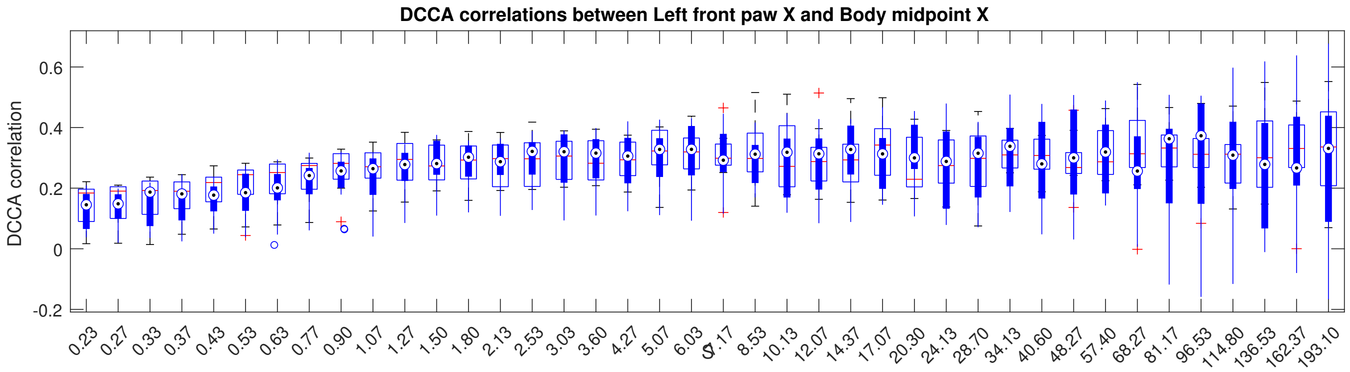


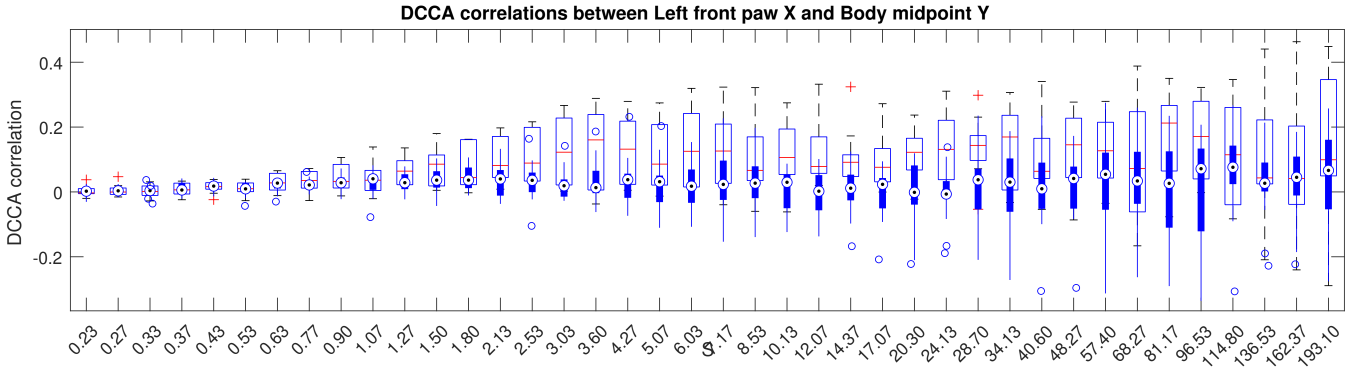


**Figure S41.** Boxplots indicating correlation dynamics of the left front paw along the X-axis *relative to the animal body midpoint* as a function of scale $S$. Movements along the same Cartesian coordinate axis denote specific correlations, while movements along the other axis indicate the level of unspecific correlations. Results for animals with Alzheimer's disease are provided by filled boxes and open circles for outliers; results for wild type animals (control group) are provided by open boxes with red plus signs for outliers.

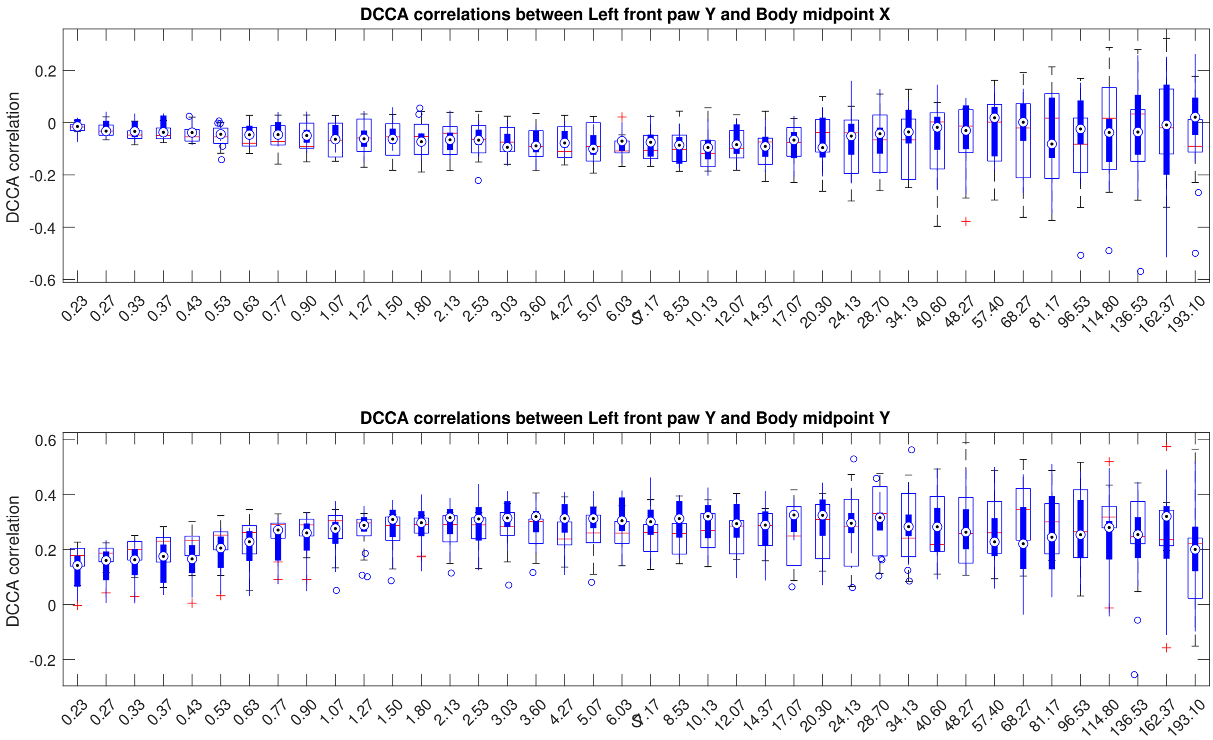


**Figure S42.** Boxplots indicating correlation dynamics of the left front paw along the Y-axis *relative to the animal body midpoint* as a function of scale $S$. Movements along the same Cartesian coordinate axis denote specific correlations, while movements along the other axis indicate the level of unspecific correlations. Results for animals with Alzheimer's disease are provided by filled boxes and open circles for outliers; results for wild type animals (control group) are provided by open boxes with red plus signs for outliers.

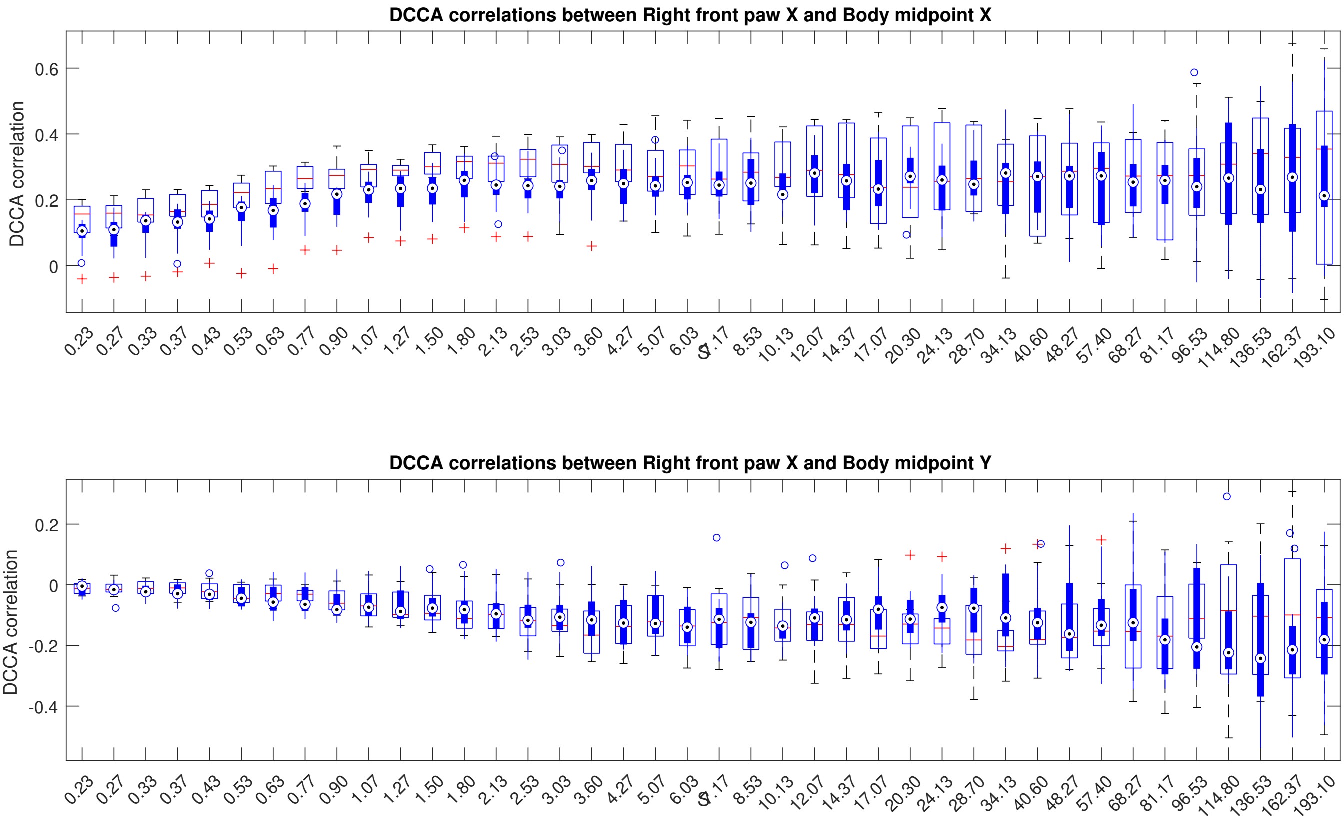


**Figure S43.** Boxplots indicating correlation dynamics of the right front paw along the X-axis *relative to the animal body midpoint* as a function of scale $S$. Movements along the same Cartesian coordinate axis denote specific correlations, while movements along the other axis indicate the level of unspecific correlations. Results for animals with Alzheimer's disease are provided by filled boxes and open circles for outliers; results for wild type animals (control group) are provided by open boxes with red plus signs for outliers.

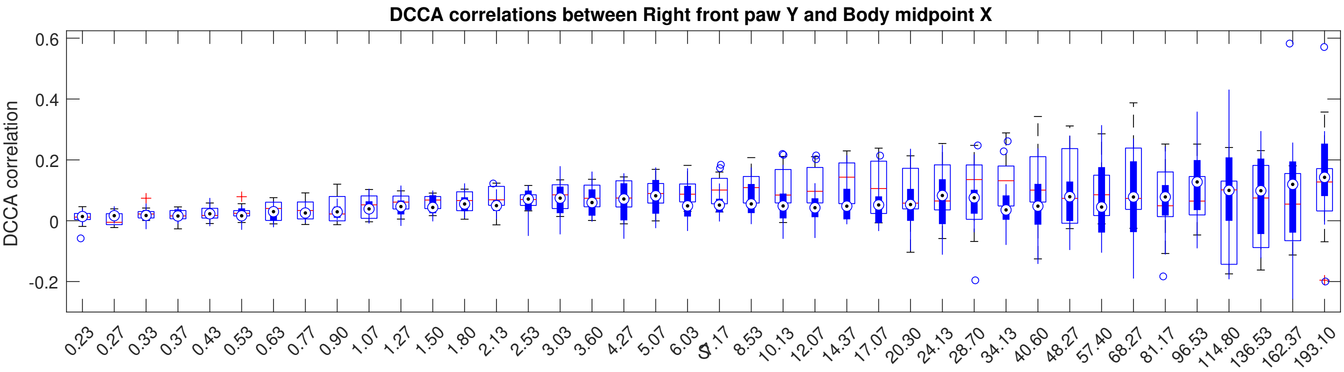


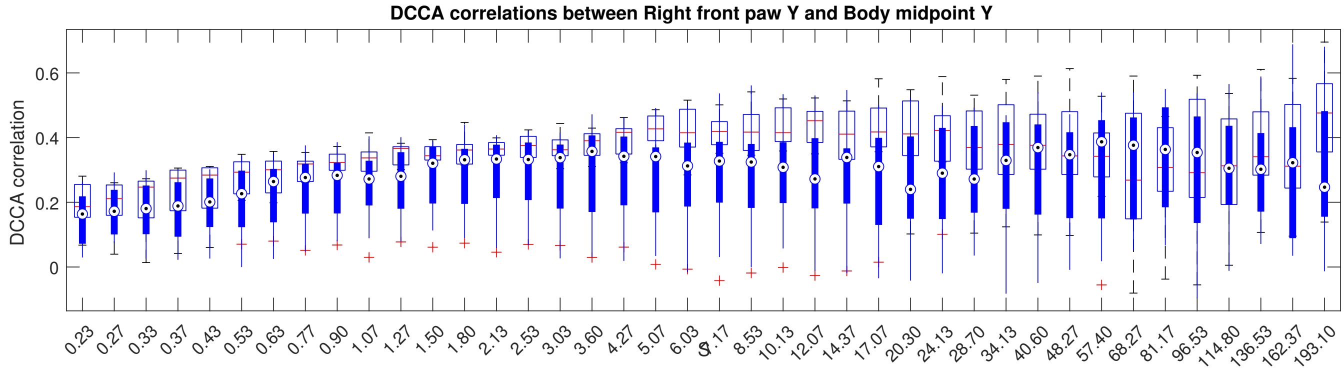


**Figure S44.** Boxplots indicating correlation dynamics of the right front paw along the Y-axis *relative to the animal body midpoint* as a function of scale $S$. Movements along the same Cartesian coordinate axis denote specific correlations, while movements along the other axis indicate the level of unspecific correlations. Results for animals with Alzheimer's disease are provided by filled boxes and open circles for outliers; results for wild type animals (control group) are provided by open boxes with red plus signs for outliers.

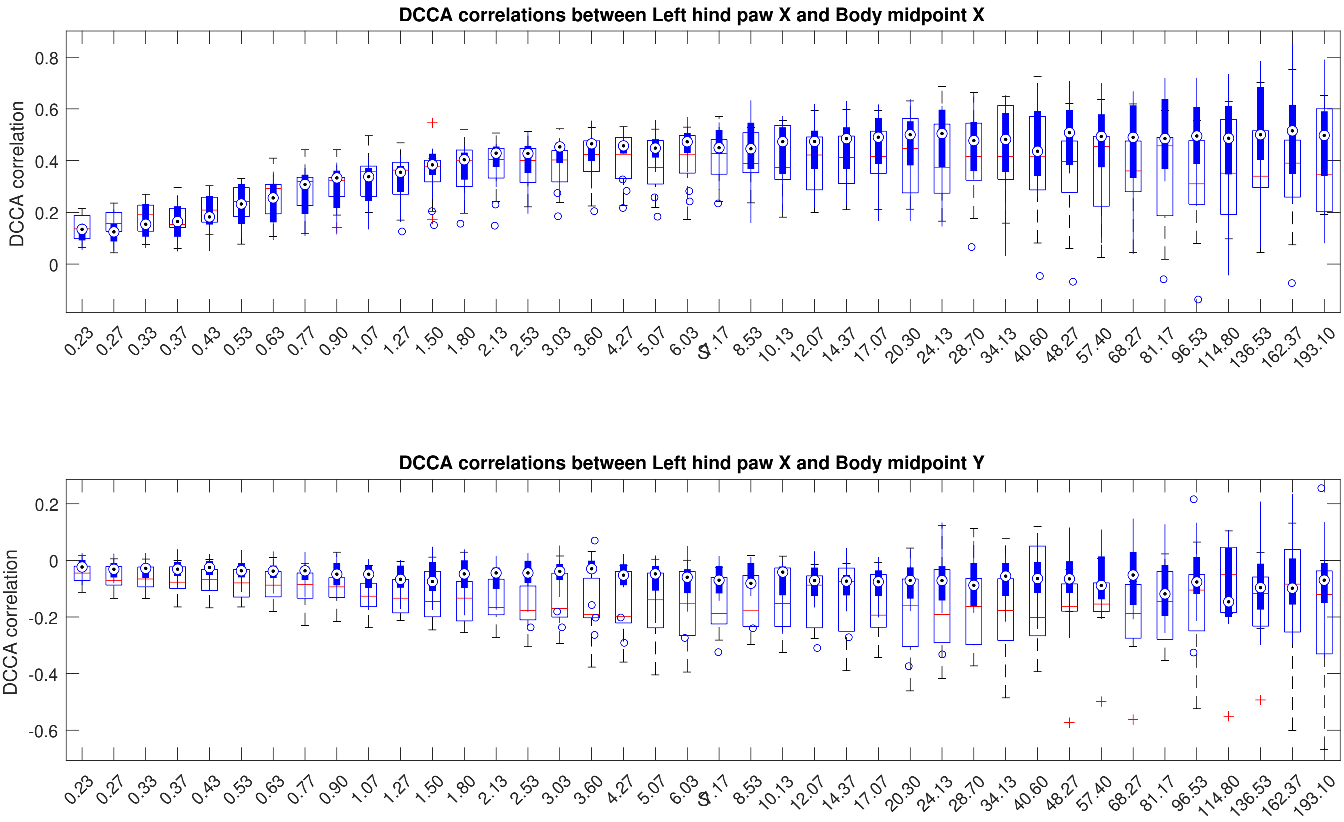


**Figure S45.** Boxplots indicating correlation dynamics of the left hind paw along the X-axis *relative to the animal body midpoint* as a function of scale $S$. Movements along the same Cartesian coordinate axis denote specific correlations, while movements along the other axis indicate the level of unspecific correlations. Results for animals with Alzheimer's disease are provided by filled boxes and open circles for outliers; results for wild type animals (control group) are provided by open boxes with red plus signs for outliers.

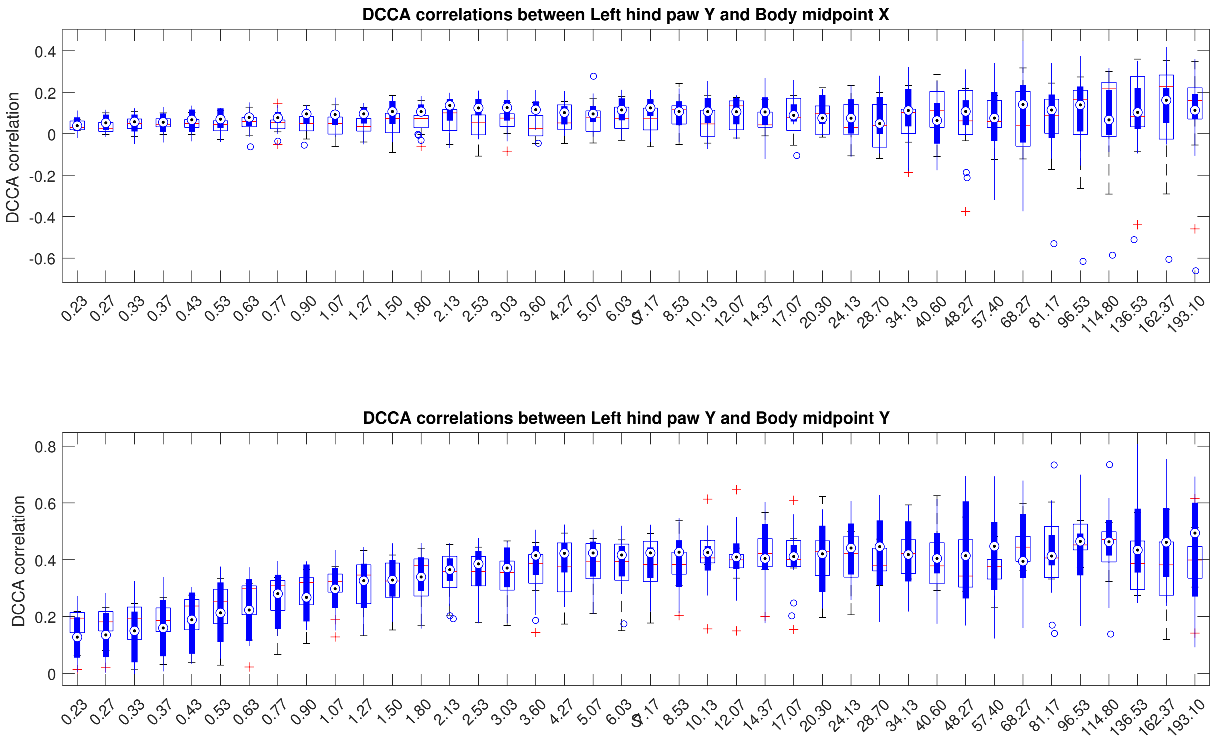


**Figure S46.** Boxplots indicating correlation dynamics of the left hind paw along the Y-axis *relative to the animal body midpoint* as a function of scale $S$. Movements along the same Cartesian coordinate axis denote specific correlations, while movements along the other axis indicate the level of unspecific correlations. Results for animals with Alzheimer's disease are provided by filled boxes and open circles for outliers; results for wild type animals (control group) are provided by open boxes with red plus signs for outliers.

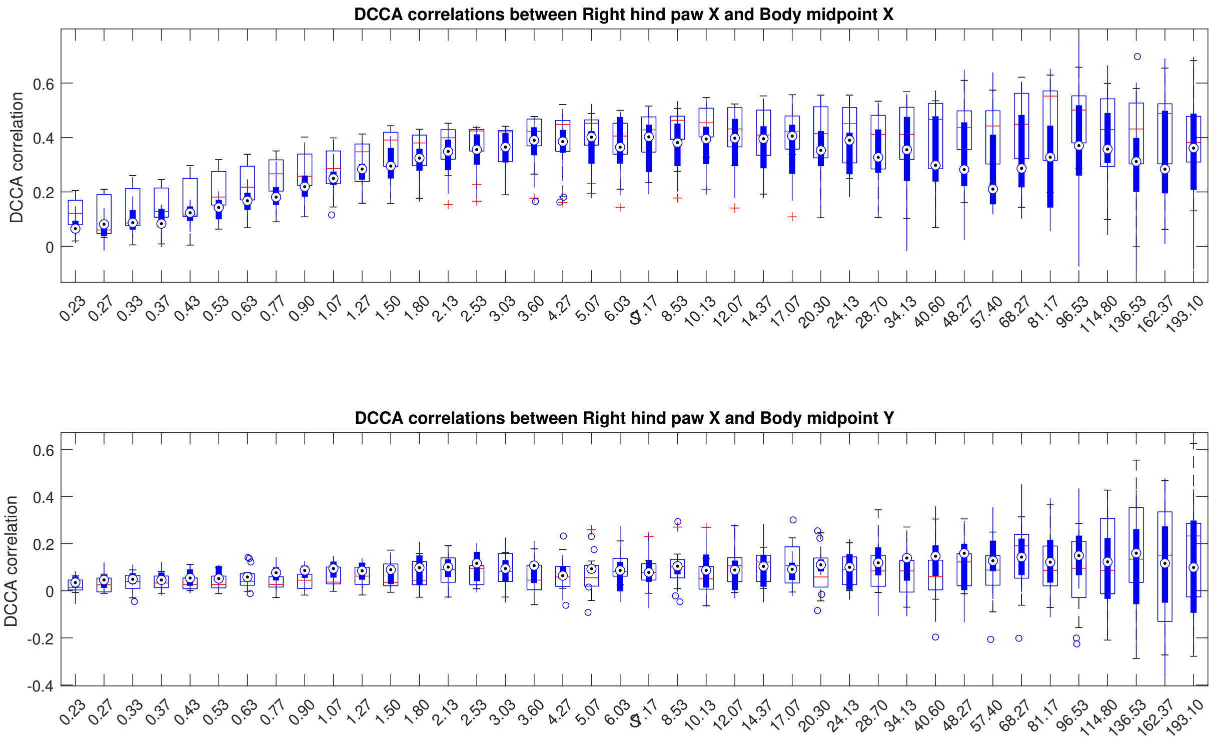


**Figure S47.** Boxplots indicating correlation dynamics of the right hind paw along the X-axis *relative to the animal body midpoint* as a function of scale $S$. Movements along the same Cartesian coordinate axis denote specific correlations, while movements along the other axis indicate the level of unspecific correlations. Results for animals with Alzheimer's disease are provided by filled boxes and open circles for outliers; results for wild type animals (control group) are provided by open boxes with red plus signs for outliers.

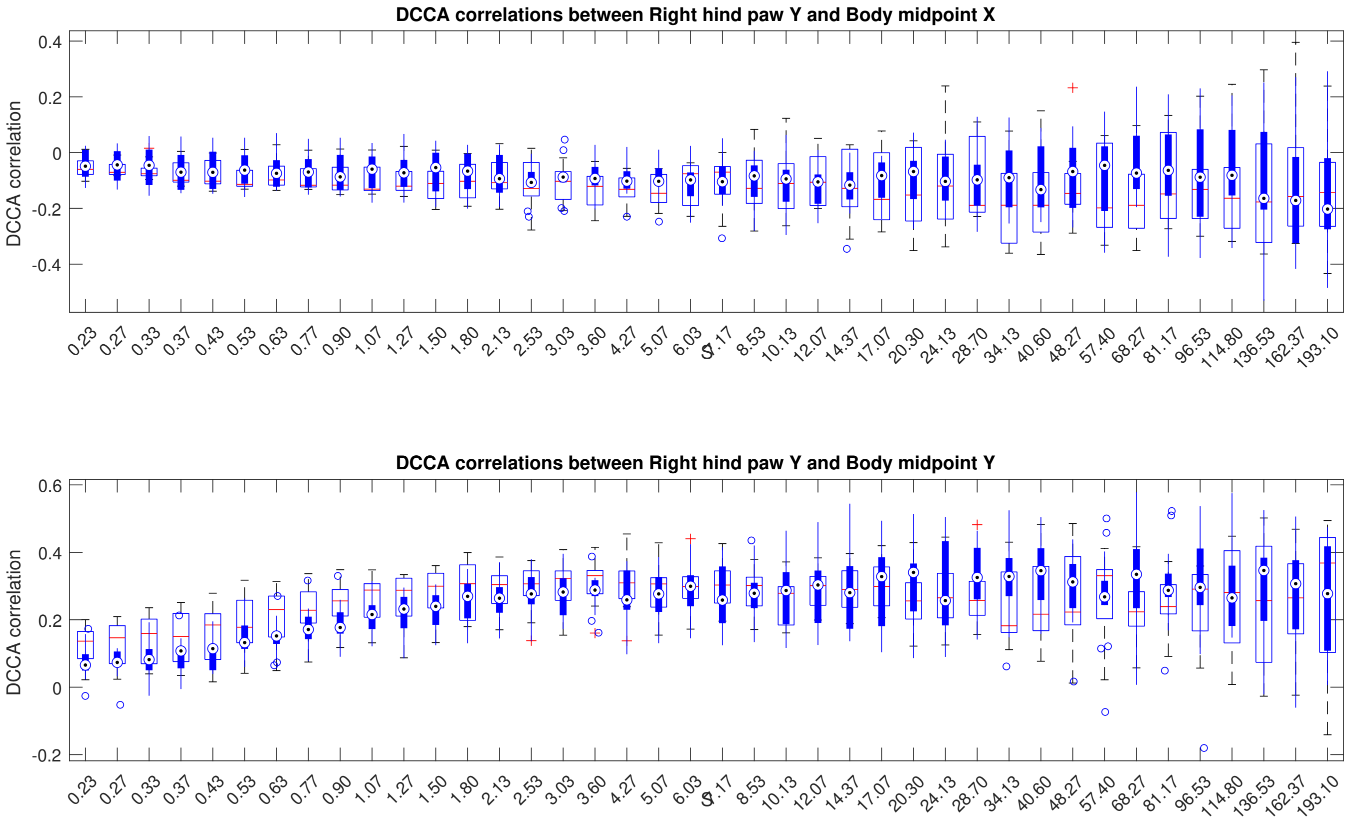


**Figure S48.** Boxplots indicating correlation dynamics of the right hind paw along the Y-axis *relative to the animal body midpoint* as a function of scale $S$. Movements along the same Cartesian coordinate axis denote specific correlations, while movements along the other axis indicate the level of unspecific correlations. Results for animals with Alzheimer's disease are provided by filled boxes and open circles for outliers; results for wild type animals (control group) are provided by open boxes with red plus signs for outliers.

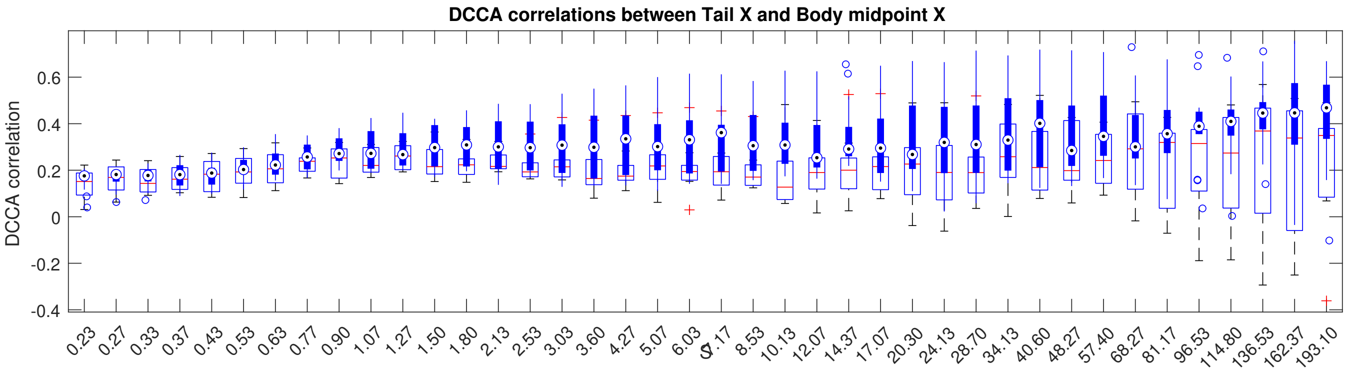


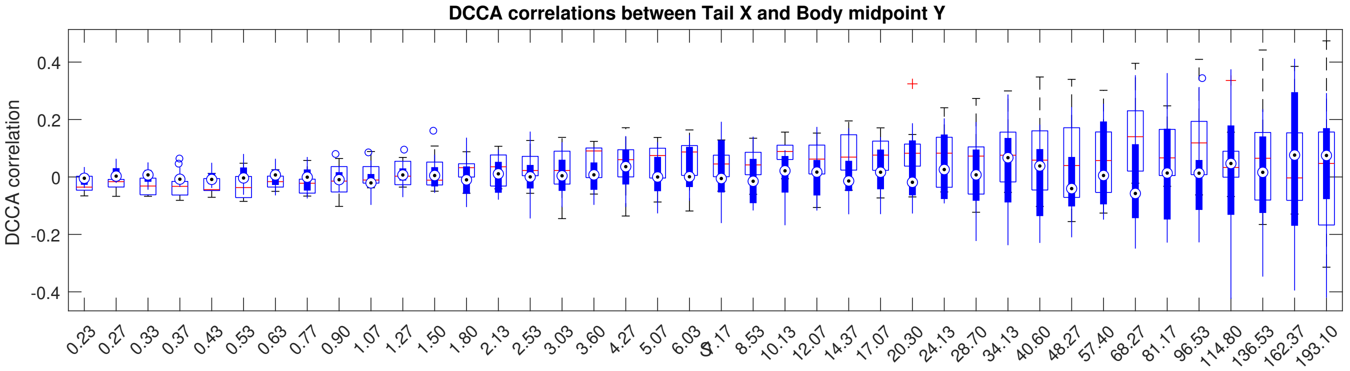


**Figure S49.** Boxplots indicating correlation dynamics of the tail along the X-axis *relative to the animal body midpoint* as a function of scale $S$. Movements along the same Cartesian coordinate axis denote specific correlations, while movements along the other axis indicate the level of unspecific correlations. Results for animals with Alzheimer's disease are provided by filled boxes and open circles for outliers; results for wild type animals (control group) are provided by open boxes with red plus signs for outliers.

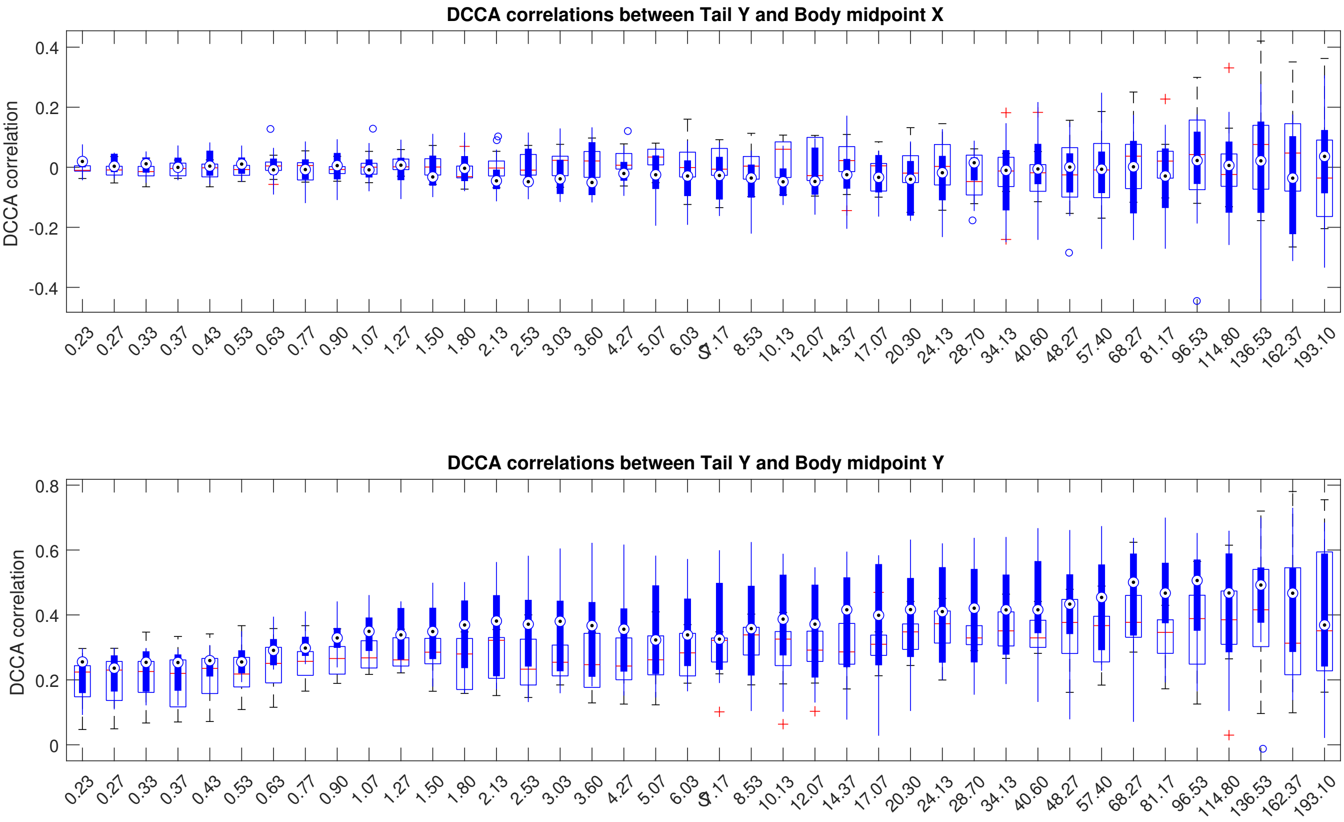


**Figure S50.** Boxplots indicating correlation dynamics of the tail along the Y-axis *relative to the animal body midpoint* as a function of scale $S$. Movements along the same Cartesian coordinate axis denote specific correlations, while movements along the other axis indicate the level of unspecific correlations. Results for animals with Alzheimer's disease are provided by filled boxes and open circles for outliers; results for wild type animals (control group) are provided by open boxes with red plus signs for outliers.
